# XPO1 blockade with KPT-330 promotes apoptosis in cutaneous T-cell lymphoma by activating the p53–p21 and p27 pathways

**DOI:** 10.1038/s41598-024-59994-5

**Published:** 2024-04-23

**Authors:** Nitin Chakravarti, Amy Boles, Rachel Burzinski, Paola Sindaco, Colleen Isabelle, Kathleen McConnell, Anjali Mishra, Pierluigi Porcu

**Affiliations:** 1 Division of Hematologic Malignancies, Sidney Kimmel Cancer Center, Thomas Jefferson University, Philadelphia, PA 19107 USA; 2 Division of Hematologic Malignancies, Sidney Kimmel Cancer Center, Thomas Jefferson University, 834 Chestnut Street, Suite 320, Philadelphia, PA 19107 USA; 3 Division of Hematologic Malignancies, Sidney Kimmel Cancer Center, Thomas Jefferson University, 233 South 10th Street, BLSB 328, Philadelphia, PA 19107 USA

**Keywords:** Haematological cancer, Molecular medicine

## Abstract

Dysregulated nuclear-cytoplasmic trafficking has been shown to play a role in oncogenesis in several types of solid tumors and hematological malignancies. Exportin 1 (XPO1) is responsible for the nuclear export of several proteins and RNA species, mainly tumor suppressors. KPT-330, a small molecule inhibitor of XPO1, is approved for treating relapsed multiple myeloma and diffuse large B-cell lymphoma. Cutaneous T-cell lymphoma (CTCL) is an extranodal non-Hodgkin lymphoma with an adverse prognosis and limited treatment options in advanced stages. The effect of therapeutically targeting XPO1 with KPT-330 in CTCL has not been established. We report that XPO1 expression is upregulated in CTCL cells. KPT-330 reduces cell proliferation, induces G1 cell cycle arrest and apoptosis. RNA-sequencing was used to explore the underlying mechanisms. Genes associated with the cell cycle and the p53 pathway were significantly enriched with KPT-330 treatment. KPT-330 suppressed XPO1 expression, upregulated p53, p21^WAF1/Cip1^, and p27^Kip1^ and their nuclear localization, and downregulated anti-apoptotic protein (Survivin). The in vivo efficacy of KPT-330 was investigated using a bioluminescent xenograft mouse model of CTCL. KPT-330 blocked tumor growth and prolonged survival (*p* < 0.0002) compared to controls. These findings support investigating the use of KPT-330 and next-generation XPO1 inhibitors in CTCL.

## Introduction

In mammalian cells, the transport of proteins between the nucleus and the cytoplasm plays a critical role in maintaining cellular homeostasis and regulating cell survival and death^1^. Aberrant intracellular localization of growth-regulatory and tumor-suppressor proteins (TSPs) is a hallmark of cancer^2^. Cancer cells acquire contrivances to sequester TSPs in the cytoplasm, resulting in cancer progression and resistance to chemotherapy. Karyopherins, nucleocytoplasmic transport receptors, are a group of proteins regulating molecules' transport between the nucleus and the cytoplasm^3^. Exportin 1 (XPO1), a nuclear export protein also known as CRM1 (chromosome region maintenance 1 protein), regulates the export of a range of cargo proteins, including TSPs and cell cycle regulatory proteins, from the nucleus to the cytoplasm^4^. Due to their higher cellular proliferation rates and metabolism, tumor cells depend heavily on nuclear transport compared to normal cells. Multiple studies have shown the upregulated expression of XPO1 protein in various solid and hematological malignancies^5,6^.

Cutaneous T-cell lymphoma (CTCL) is an incurable, chemo-resistant extranodal lymphoma of mature CD4+ T-cells that presents in the skin and, in many patients, progresses to involve lymph nodes, blood, and viscera. Patients with extra-cutaneous involvement have a survival of less than two years. Novel therapeutic approaches are needed. Studies have revealed several molecular drivers in CTCL, including deregulation of p53 and NFkB signaling^7^, both of which are regulated by XPO1-mediated nucleocytoplasmic transport. Recently, XPO1 overexpression was shown to be associated with decreased survival in other T-cell lymphomas^8^. Inhibition of XPO1 expression results in tumor cell growth inhibition in T-cell lymphoma cell lines in vitro^8,9^. Thus, the ability to alter the nucleocytoplasmic interchange of essential cargo proteins like p53, p21^WAF1/Cip1^, cyclin B1/D1, and IκB by inhibiting XPO1 creates opportunities to target diverse pathways implicated in carcinogenesis, making XPO1 an attractive target for novel cancer therapies.

Aberrant nuclear export has been explored as a target of cancer therapy. The initial attempts to block the XPO1 pathway in pre-clinical studies were successful, but the agents that were tested in vivo were also toxic^10^. This led to the development of the SINE (selective inhibitor of nuclear export) compound KPT-330, which specifically targets XPO1 by irreversibly binding to the nuclear export groove of XPO1 and causing its degradation^11,12^. In pre-clinical studies, KPT-330 shows antitumor activity at sub-micromolar concentrations in several solid and hematological malignancies^4,13,14^ and is FDA-approved (Selinexor) for the treatment of refractory and relapsed multiple myeloma and diffuse large B-cell lymphoma^15^.

Our study explores the antitumor effects of XPO1 inhibition with KPT-330 in CTCL. We describe the mechanisms of XPO1 inhibitor-mediated inhibition of proliferation and induction of apoptosis in CTCL cell lines and the in vivo antitumor efficacy of KPT-330 in a murine xenograft model of CTCL.

## Results

### KPT-330 treatment reduces XPO1 levels and inhibits CTCL cell growth in vitro

**Figure 1:**
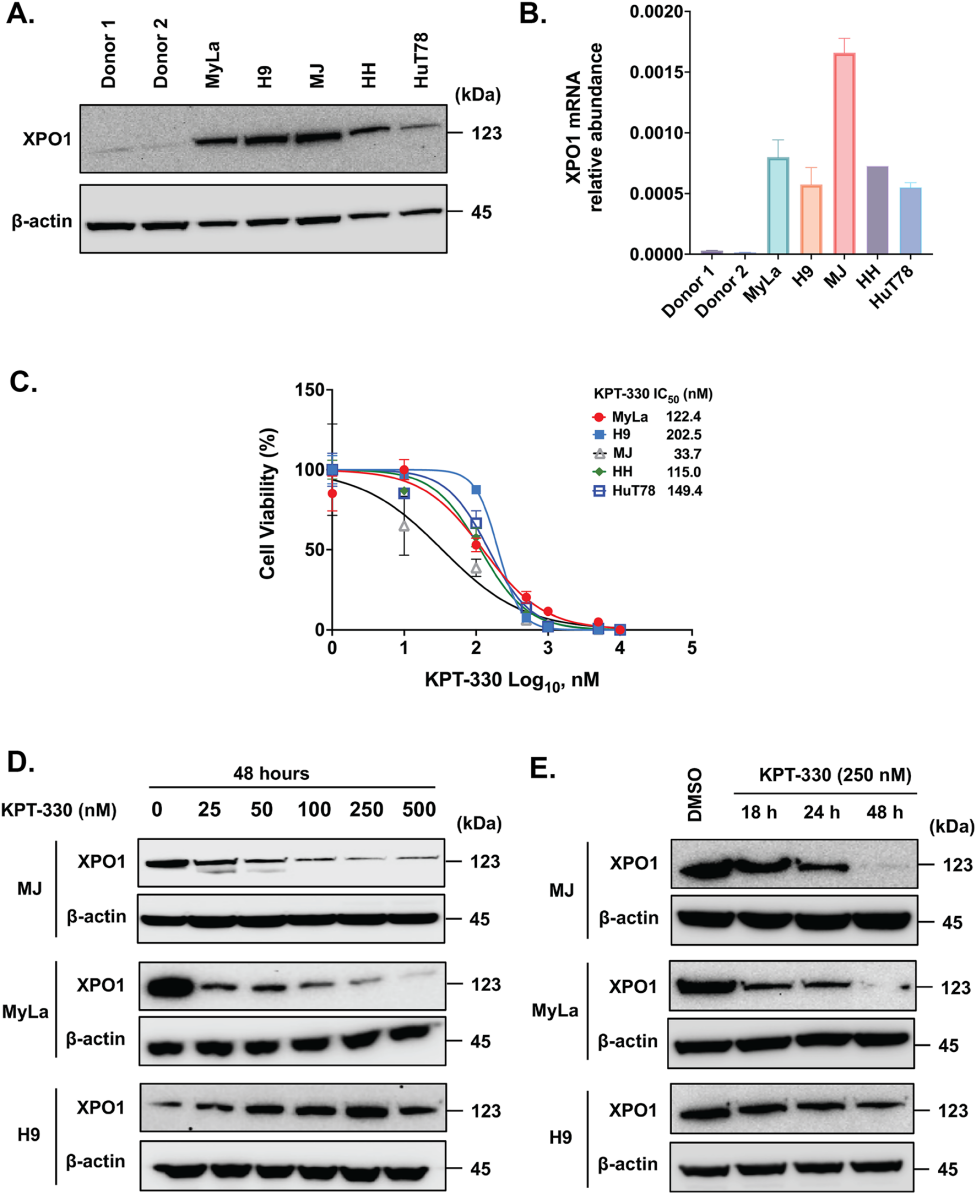
Expression of XPO1 in CTCL (**A**) XPO1 overexpression in CTCL cell lines compared to healthy donor CD4+ T-cells detected by immunoblot analysis. Healthy donor PBMCs were obtained from Leukocyte reduction filters supplied by the Thomas Jefferson University Hospital Blood Bank Laboratory under an Institutional Review Board approved protocol. β-actin serves as an internal control. (**B**) *XPO1* gene expression in CTCL cells detected by q–PCR. Relative gene expression is represented as the average relative quantity on the Y-axis. Selective inhibitor of nuclear export (SINE) compound reduces cell viability and downregulates XPO1 at the protein level—(**C**) Dose–response curves for 5 CTCL cell lines (MyLa, H9, MJ, HH, and HuT78) treated with SINE. The cells were treated with KPT-330 at different concentrations for 72 h. Cell viability was measured using the MTT, and IC_50_ values were then calculated; mean values and standard deviation of technical triplicates. Western blot for XPO1 protein expression in MJ, MyLa, and H9 cell lines (**D**) after treatment with KPT-330 at lower and higher doses, and (**E**) a time course at 18, 24, and 48 h after treatment with KPT-330 (250 nM).

To determine the expression of endogenous XPO1 protein in CTCL, we first performed a western blot analysis in CD4+ T cells from two healthy donors and five well-characterized CTCL cell lines (MyLa, MJ, H9, Hut78, and HH), originally obtained from patients with mycosis fungoides (MF) and Sézary syndrome (SS). An increased expression of XPO1 protein and mRNA was observed in all five CTCL cell lines, compared to CD4+ T-cells from healthy donors (Fig. 1A and B, respectively). The relative expression of the XPO1 transcript in various CTCL cell lines correlated to high XPO1 protein levels. These results demonstrated that XPO1 is significantly overexpressed in multiple CTCL cell lines and that mRNA levels parallel the expression of XPO1 protein.

To examine the biological role of XPO1 in CTCL cells, we assessed the dose-dependent effects of XPO1 inhibition with KPT-330 in the MyLa, MJ, H9, Hut78, and HH cell lines. We treated CTCL cells with varying doses of KPT-330 (0, 1, 10, 50, 100, 250, and 500 nM), assessed viability at 72 h using the MTS assay, and calculated the 50% growth-inhibitory effect (IC_50_). KPT-330 led to significant dose-dependent inhibition of cellular proliferation in all cell lines. Figure 1C shows the growth curves for MJ, MyLa, HH, HuT78, and H9 cells after exposure to sub-micromolar concentrations of KPT-330 for 72 h. The IC_50_ for MJ cells was 33.7 nM, whereas the IC_50_ for the other cell lines, including MyLa, HH, and HuT78, ranged from 115 to 149.4 nM, and H9 displayed an IC_50_ above 202.5 nM. These data suggest a correlation between the growth inhibition efficacy of KPT-330 and XPO-1 expression, with higher XPO-1 levels correlating with lower IC_50_ values. Therefore, three cell lines with high (MJ), medium (MyLa), and low (H9) sensitivity to KPT-330 were selected for further molecular analyses.

To demonstrate that the growth inhibitory effects of KPT-330 are mediated through XPO1 degradation, XPO1 expression was measured in MJ, MyLa, and H9 cells after treating them with increasing concentrations of KPT-330 (0–500 nM, 48 h). As expected, treatment with KPT-330 led to a dose-dependent (Fig. 1D) and time-dependent (Fig. 1E) decrease in XPO1 protein expression levels in all three CTCL cell lines as early as 24 h. This shows that the growth inhibitor effect of KPT-330 correlates with a decrease in XPO1 levels.

### RNA-seq analysis identifies differentially expressed genes in KPT-330 treated CTCL cells

**Figure 2:**
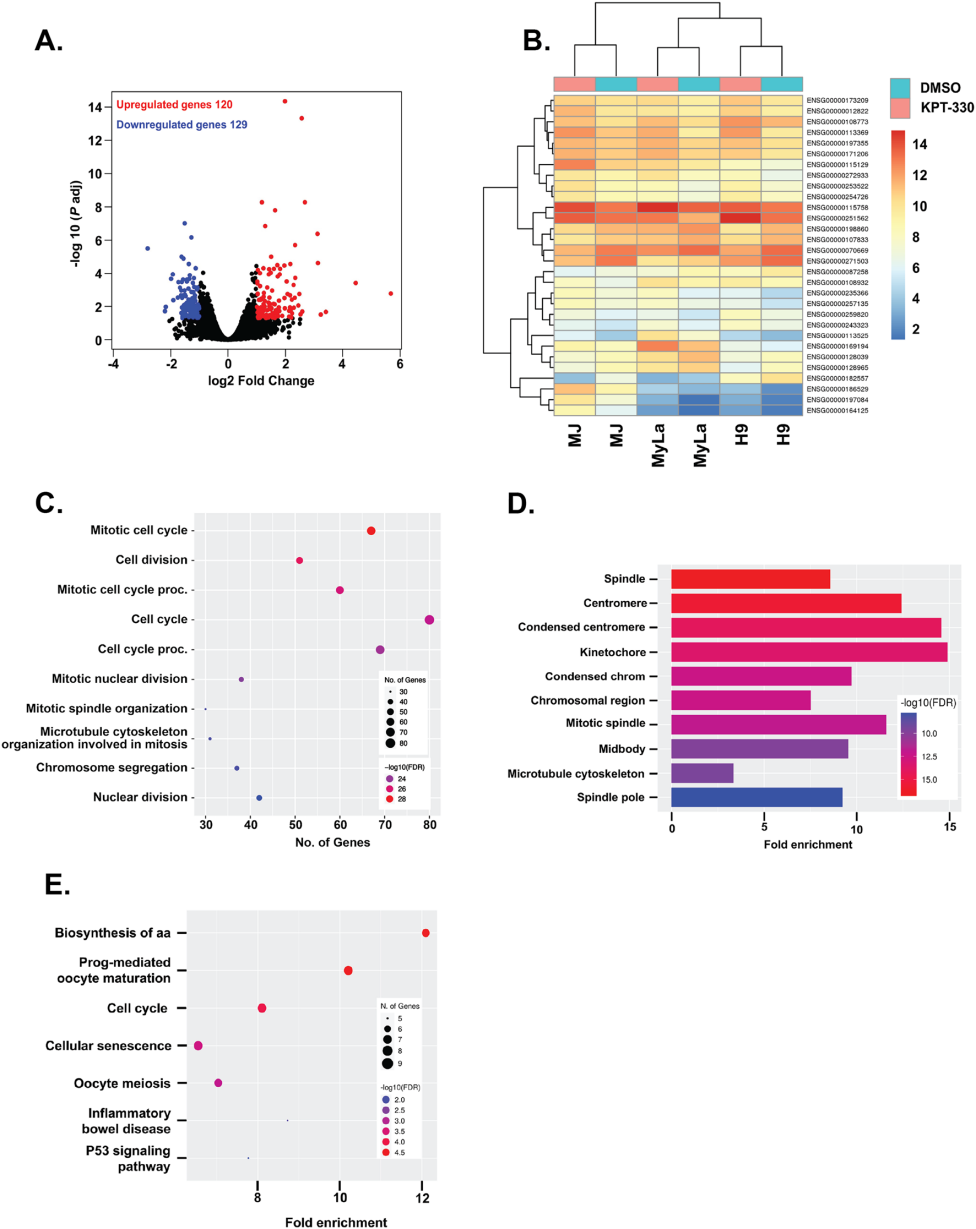
RNA-seq analysis of KPT-330-treated CTCL cells (**A**) Visualization of differentially expressed genes (DEGs) volcano plots using R studio. The plot compared the DEGs between KPT-330-treated cells and controls. Each data point in the scatter plot represents a gene. The log2 fold change of each gene is represented on the x-axis, and the log10 of its adjusted *p*-value is on the y-axis. Genes with an adjusted *p*-value less than 0.05 and a log2 fold change greater than 1 are indicated by red dots. These represent upregulated genes. Genes with an adjusted *p-*value less than 0.05 and a log2 fold change less than − 1 are indicated by blue dots. These represent down-regulated genes. Black dots indicate the remaining genes present in the datasets that were not significantly changed. (**B**) DEGs heat map and clustering based on hierarchy for the top 30 gene expression data from the datasets. n = 30 DEGs. Upregulated genes are represented in red, and downregulated genes are represented in blue. Significantly DEGs were clustered by their gene ontology, and the enrichment of gene ontology terms was tested using Fisher exact test (GeneSCF v1.1-p2). The figures **C**–**E** show gene ontology terms, if any, that are significantly enriched with an adjusted *P*-value less than 0.05 in the differentially expressed gene sets (up to 10 terms).

To analyze global transcriptional alterations in CTCL due to KPT-330-induced XPO1 inhibition, we performed a Poly(A) RNA-sequencing of MJ, MyLa, and H9 cells treated with either vehicle (DMSO) or KPT-330. We extracted RNA from treated cells at 48 h and compared the transcriptomes of cells treated with KPT-330 or DMSO. We examined differentially expressed genes by comparing KPT-330-treated cells with their DMSO controls and identified differentially expressed genes in KPT-330-treated CTCL cells. The results showed that 120 transcripts were upregulated and 129 were downregulated in KPT-330 treated cells (Fig. 2A). The heat map in Fig. 2B shows the top 30 differentially expressed genes in KPT-330 treated cell lines. According to the Gene Ontology (GO) analysis, the differentially expressed genes were closely associated with “cell division”, “cell cycle”, “nuclear division”, and “cytoskeleton” (Fig. 2C). The GO cellular component showed that the differentially expressed genes were related to the “spindle”, “nucleus”, and “cytoskeleton” (Fig. 2D). Thus, GO analysis results revealed that changes were closely related to the role of XPO1 in nucleocytoplasmic transport. The KEGG pathway^16,17^ enrichment analysis results suggested important changes in the “p53 signaling pathway”, “senescence”, “cell cycle”, and “meiosis” genes according to the fold enrichment (Fig. 2E). Overall, the results of this bioinformatics analysis indicated that XPO1 inhibitor KPT-330 induces changes in critical cancer pathways in CTCL cells.

### KPT-330 induces apoptosis and cell cycle arrest in CTCL cells

**Figure 3:**
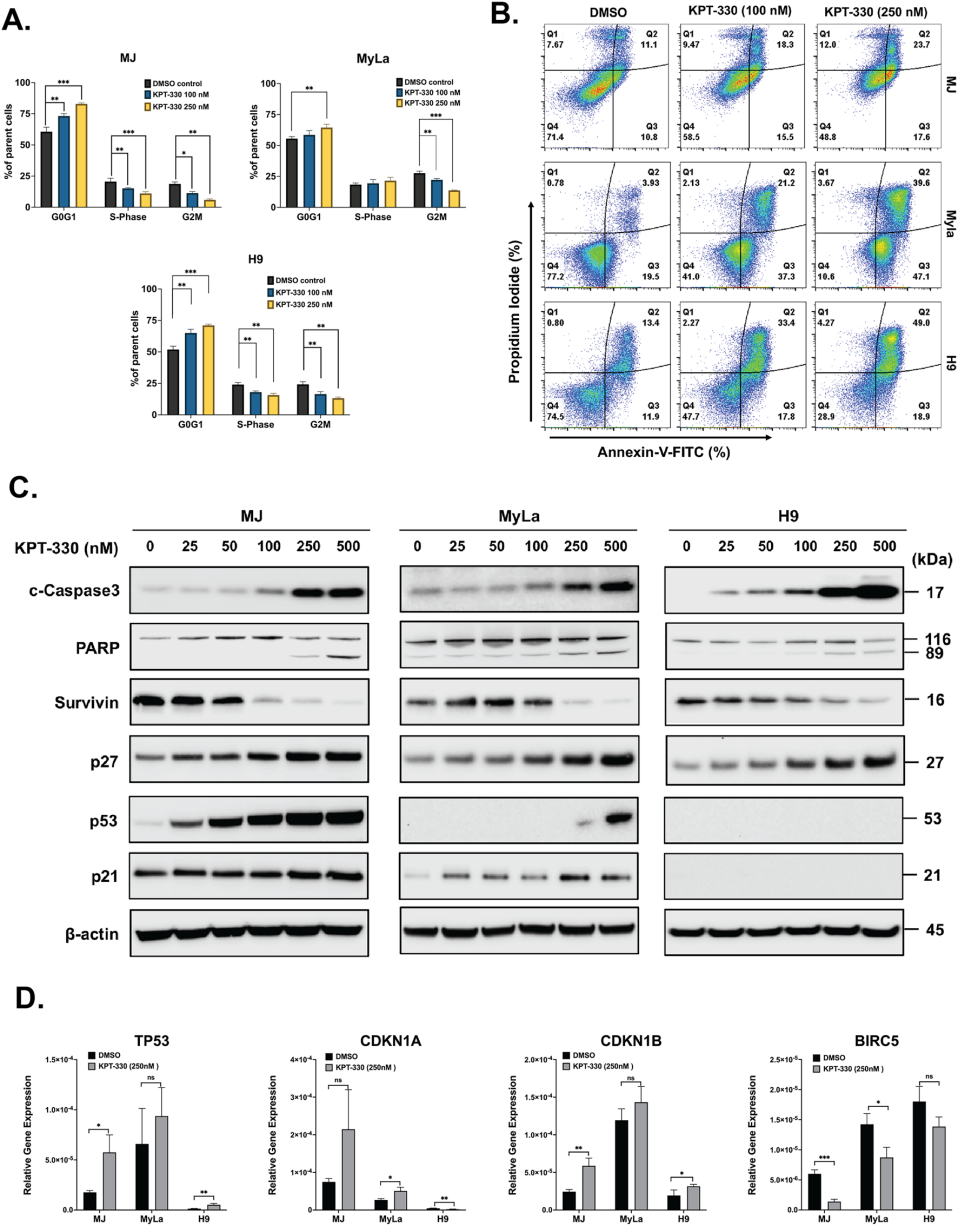
Effects of treatment with KPT-330 on cell cycle and apoptosis of various CTCL cell lines (MJ, MyLa, and H9) (**A**) Cell cycle analysis of MJ, MyLA and H9 cells after exposure to KPT-330 for 48 h. Cells were stained with PI and analyzed by flow cytometry (representative results of 3 experiments). (**B**) Apoptosis of CTCL cells after exposure to KPT-330 for 48 h. Cells were stained with PI and AnnexinV-FITC and analyzed by flow cytometry (representative results of 3 experiments). (**C**) CTCL cells were treated with indicated doses of KPT-330 for 48 h and subjected to western blot. (**D**) *TP53*, *CDKN1A*, *CDKN1B*, and *BIRC5* gene expression in CTCL cells detected by q–PCR. The data were analyzed using 18S as the reference gene and represent the mean ± SD from 3 independent experiments (*p* < 0.05: *, *p* < 0.01: **, *p* < 0.001: ***). Relative gene expression is represented as the average relative quantity on the Y-axis.

CTCL cell lines were treated with KPT-330 (100 & 250 nM) or DMSO control for 48 h, and cell cycle distributions were determined by staining with propidium iodide (PI). Treatment with KPT-330 led to the accumulation of CTCL cells in the G1 phase and reduced the number of cells in the S and G2/M phases (Fig. 3A). Flow cytometric analysis demonstrated a dose-dependent increase in the percentage of apoptotic cells (PI^high^/AnV^high^) and a parallel decrease in viability in all three CTCL cell lines (PI^low^/AnV^low^) after treatment with KPT-330 (100 & 250 nM, 48 h) (Fig. 3B).

Next, to investigate the mechanism of the KPT-330 induced apoptosis, we examined the expression profiles of apoptotic proteins in drug-treated CTCL cells (Fig. 3C). Western blot analysis showed that KPT-330 increased cleaved caspase-3 as well as PARP cleavage compared to vehicle control in a dose-dependent manner. Conversely, a decrease in anti-apoptotic survivin expression was also observed, suggesting mitochondrial apoptosis in KPT-330 treated cells. In addition, KPT-330 increased the levels of p27^Kip1^ in a dose-dependent manner across all three cell lines. p53 is the cargo protein of XPO1 and plays a vital role in cell apoptosis. Concomitantly, KPT-330 (0–500 nM, 48 h) prominently increased protein levels of p53 and p21^WAF1/Cip1^ (Fig. 3C) in MJ and MyLa cells. H9 cells showed no detectable levels of p53 and p21^WAF1/Cip1^ proteins. To further understand how KPT-330 modulated p53, p21^WAF1/Cip1^, p27^Kip1^, and survivin protein expression levels in CTCL cells, we checked the transcript levels of these target proteins in cells treated with KPT-330 (250 nM) for 48 h. KPT-330 increased the mRNA expression levels of *TP53*, *CDKN1A*, and *CDKN1B* and decreased the expression of *BIRC5* mRNA in CTCL cell lines (Fig. 3D). These data indicated that KPT-330 induces cell cycle arrest and cell apoptosis in vitro by influencing several tumor suppressor genes.

### p27^Kip1^, p53 and its target protein p21^WAF1/Cip1^ are retained in the nucleus following the inhibition of XPO1

**Figure 4:**
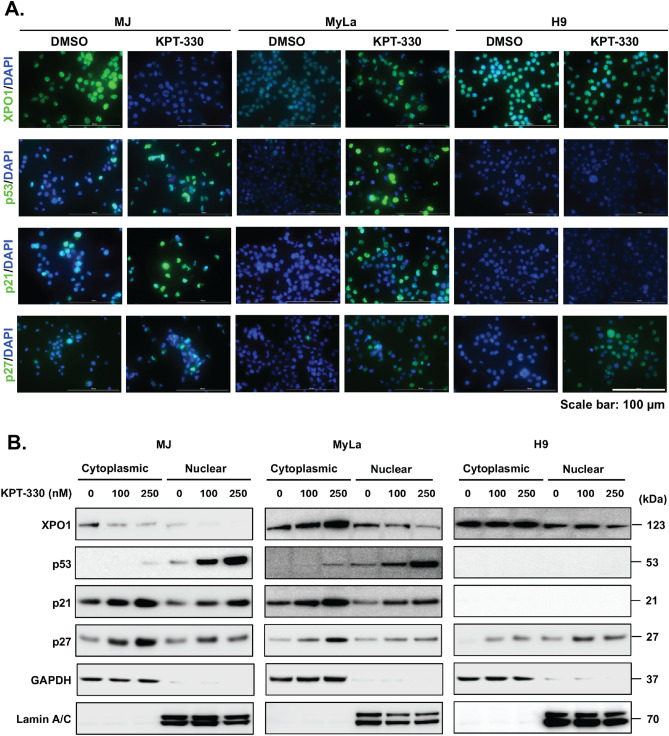
KPT-330 modulates cellular localization of XPO1, p53, p21, and p27 in CTCL cells (**A**) XPO1 and its cargo proteins cellular localization assessed by confocal microscopy in MJ, MyLA, and H9 cells after treatment with DMSO (control) or KPT-330 (250 nM) for 48 h. DAPI stains nuclei. Scale bars, 100 µm. (**B**) Immunoblot of protein expression in cytoplasmic and nuclear fractions of CTCL cells treated with KPT-330. Lamin A/C and GAPDH serve as loading controls for nuclear and cytoplasmic fractions, respectively.

To elucidate the antitumor activity of the KPT-330 in the CTCL cells, we examined the efficiency of nuclear export and spatial expression of XPO1 protein and its target cargo proteins in KPT-330-treated cells. Immunofluorescence analyses were performed to assess nuclear export function, evaluating the nuclear-cytoplasmic localization changes of different XPO1 cargo proteins post-KPT-330 treatment for 48 h. The results of Fig. 4A demonstrated that the KPT-330 treatment caused substantial nuclear retention of the p53, p21^WAF1/Cip1^, and p27^Kip1^ proteins compared to the control.

Next, we looked at the potential target proteins of KPT-330 by immunoblotting cytosolic and nuclear protein fractions in MJ, MyLa, and H9 cells (Fig. 4B). Our results show KPT-330 treatment elevates p53 (primarily nuclear) and p21^WAF1/Cip1^ (cytoplasmic and nuclear) in all but H9 cells. Additionally, a dose-dependent accumulation of the p27^Kip1^ (cytoplasmic and nuclear) in all cell lines was observed after 48 h of exposure to KPT-330. Our results suggest that inhibiting XPO1 increases p53, p21^WAF1/Cip1^, and p27^Kip1^ proteins, predominantly in the nucleus.

### KPT-330 blocks tumor growth and prolongs survival in a bioluminescent xenograft mouse model of CTCL

**Figure 5:**
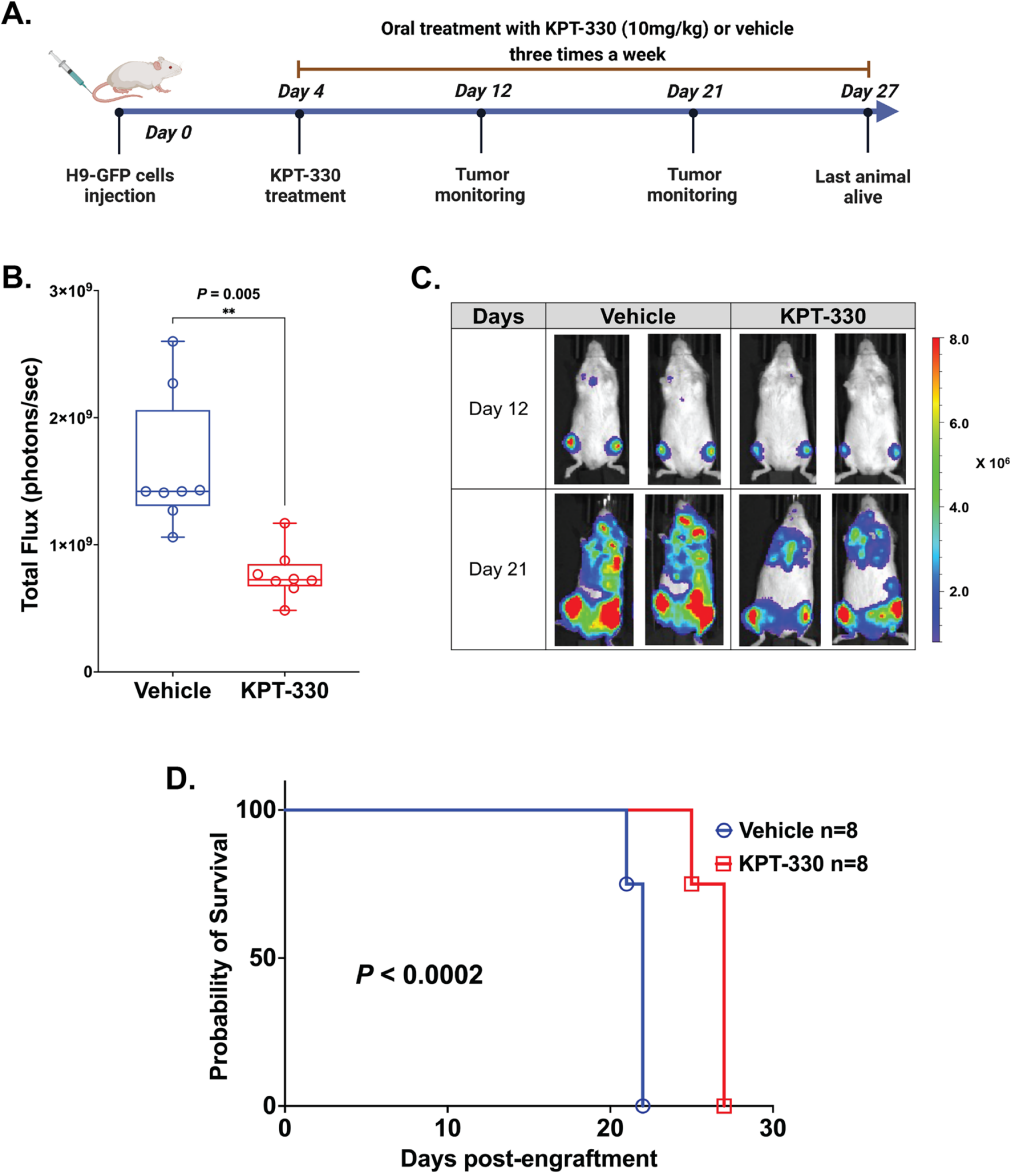
Pre-clinical efficacy of KPT-330 in mice xenograft models (**A**) Schematic diagram showing experiment design: H9 cells were transduced with the lentivirus expressing firefly luciferase-GFP (H9-Luc-GFP), sorted to > 99% purity, and transplanted into mice with 2 million cells. Mice were evaluated for leukemia cell engraftment on an IVIS imager (Perkins-Elmer). After confirming the engraftment of lymphoma cells in vivo (7 days), cohorts of mice were randomly assigned to treatment with vehicle (DMSO, PEG 300, Tween 80, ddH20) or 10 mg/kg KPT-330 (via oral-gavage) three times a week for four weeks and imaged weekly. Figure made with biorender.com. (**B**) Luciferase bioluminescence was quantified by measuring total flux (photons/second) in mice using the IVIS-100 and Living Image software at day 21 (vehicle n = 8, KPT-330 treated n = 8, *p* = 0.005 by student t-test). (**C**) Representative dorsal bioluminescence images of mice demonstrating lymphoma burden at days 12 and 21 in the vehicle and KPT-330 treated cohorts. (**D**) Kaplan–Meier curve shows effect of KPT-330 treatment (10 mg/kg, 3 times weekly) on survival of NRG mice injected with H9-Luc-GFP cells (n = 8, red line). Untreated mice injected with cells (n = 8, blue line) were used as controls. Survival was calculated by the Kaplan–Meier method, and the log-rank test evaluated the differences among survival distributions: *p* < 0.0002 (untreated vs KPT-330–treated mice).

We next sought to elucidate whether the KPT-330 antitumor efficacy observed in vitro could be translated in vivo. To determine the anti-lymphoma activity of KPT-330 in CTCL, we used a murine xenograft model established by intravenous injection of the luciferase-expressing H9 cell line into the tail vein of the NOD-rag-gamma (NRG) immunodeficient mice. Despite its lower sensitivity to KPT-330, compared to MJ and MyLa, the H9 cell line was selected because it has successfully been used before in a xenograft model in NRG mice for pre-clinical studies^18^. Lymphoma cell engraftment was monitored using IVIS-Spectrum bioluminescence measurement. Four days after the injection of cells, all animals had detectable lymphoma and were randomly distributed into treatment (n = 8) or vehicle (n = 8) experimental groups. Mice were dosed with either 10 mg/kg of KPT-330 or vehicle via oral gavage three times a week for four weeks, and subsequently, to monitor the tumor growth non-invasively, bioluminescence was assessed on days 12 and 21 post-engraftment (Fig. 5A). The dose was selected based on previous pre-clinical data in mouse models of different malignancies^19,20^. Treated mice showed a significantly slower increase in bioluminescence signal over time (paired t-test: *p* = 0.005; Fig. 5B), indicating that the treatment with KPT-330 notably reduced tumor growth (representative cases in Fig. 5C). KPT-330 was well tolerated at the tested dose with no significant body weight loss (Supplementary Figure S1). The inhibition of lymphoma growth induced by KPT-330 translated into a significantly increased survival, with a median survival of 27 days in the treatment group compared to 22 days in the vehicle group (*p* < 0.0002; Fig. 5D). These results indicate that KPT-330 effectively kills the bulk human lymphoma cells in the xenograft model of CTCL.

## Discussion

In this study, we exhibited the pre-clinical therapeutic effectiveness of KPT-330, a selective inhibitor of XPO1, in cutaneous T-cell lymphoma (CTCL) cells. Additionally, we employed high-throughput methodologies to pinpoint pathways linked to its mode of action.

XPO1 serves as a crucial nuclear export carrier for numerous TSPs^4^, offering the potential to target distinct pathways involved in carcinogenesis by modulating the nucleocytoplasmic localization of cargo proteins^13^. Our investigation revealed an upregulation of XPO1 in CTCL cells, potentially leading to the aberrant translocation of vital TSPs to the cytoplasm. Dysregulation of apoptosis is a hallmark of cancer, and triggering apoptosis in cancer cells is a potent strategy for treatment with cytotoxic anticancer agents. Prior research indicates that XPO1 inhibitors exert their anticancer effects by inducing apoptosis in cancer cells^6,12,21^. In our study, we observed that the XPO1 inhibitor KPT-330 prompted caspase-dependent apoptosis in CTCL cell lines.

The tumor suppressor p27^Kip1^ regulates cell cycle and apoptosis by modulating CDK/cyclins. Forkhead Class box O3a (FoxO3a), a protein transported by XPO1, suppresses *CDKN1B* expression when it moves from the nucleus to the cytoplasm^22^. However, XPO1 inhibition by KPT-330 sequesters FoxO3a in the nucleus, potentially increasing p27^Kip1^ expression levels^9^. Treatment with KPT-330 in this study increased p27^Kip1^ levels in three CTCL cell lines, leading to cell growth inhibition in a dose-dependent manner. Our data supports earlier reports, highlighting that the cell cycle inhibitor p27^Kip1^ plays a role in SINE compounds-mediated regulation of cell growth and death in hematological neoplasms, including T-cell lymphoma^9,23^. Additionally, CTCL is characterized by high levels of the anti-apoptotic protein survivin^24,25^, which shuttles between the nucleus and the cytoplasm, impacting apoptosis and cell cycle progression^26^. Notably, the KPT-330 treatment also led to a decrease in survivin expression levels. These results support previous studies proposing that downregulating survivin could be a viable therapeutic strategy for CTCL^25,27–30^. Future research could investigate if XPO1 inhibition induces a shift in CTCL cells from a hyperproliferative state to an apoptotic state, potentially enhanced by targeting other critical cellular processes.

Studies have demonstrated that the loss of p53 function in cutaneous T-cell lymphoma (CTCL) can impact various cellular processes, including cell cycle control and DNA repair, leading to the resistance of CTCL cells to chemotherapy^31^. Ongoing research is exploring the role of p53 in CTCL, with the development of new therapies targeting p53-defective cells in hematologic malignancies^32–35^. The H9 cell line, derived from Hut78 and representing Sézary Syndrome, carries a nonsense mutation of p53 in exon 6, while MyLa has a *TP53* gene duplication, and MJ has a wild-type *TP53*^36^. Our bioinformatic analyses confirmed that KPT-330 induces significant changes in the p53 pathway. In H9 cells, KPT-330 resulted in a notable increase in *TP53* transcript but no detectable p53 protein. However, in MJ and MyLa cell lines, the upregulation of p21^WAF1/Cip1^, a transcriptional target of p53, suggests the restoration or reactivation of p53 tumor suppressor function. The molecular function of p21^WAF1/Cip1^ depends on its subcellular localization, with nuclear p21^WAF1/Cip1^ inducing cell cycle arrest, while cytoplasmic expression corresponds to an anti-apoptotic role ^37^. Enhanced p21^WAF1/Cip1^ nuclear expression upon XPO1 inhibition indicates that XPO1 inhibition restores p53 nuclear function in the MJ and MyLa cell lines, potentially leading to cell cycle arrest and apoptosis, supporting previous findings in adult T-cell leukemia^38^.

The in vivo study demonstrated the strong anti-lymphoma efficacy of KPT-330. A phase II clinical trial of KPT-330 in relapsed or refractory peripheral T-cell lymphoma (PTCL) or CTCL was terminated due to enrollment challenges (https://clinicaltrials.gov/study/NCT02314247). However, a recent phase II trial in China showed promising response rates with Selinexor in previously untreated PTCL patients^39^. In a CTCL xenograft mouse model, we observed a 5-day increase in survival time in the KPT-330-treated group, reflecting a 23% improvement in survival compared to the untreated group. These results indicate the potential effectiveness of XPO1 inhibitors in CTCL treatment, highlighting the necessity for additional research to explore the in vivo impact of KPT-330 therapy in CTCL.

In summary, our study underscores the therapeutic promise of KPT-330, an XPO1 inhibitor, for treating cutaneous T-cell lymphoma (CTCL). Through its activation of the p27^Kip1^ and p53–p21^WAF1/Cip1^ pathways, KPT-330 demonstrates its capability to induce cell death and halt cell proliferation in CTCL cells. Further investigations, including clinical trials, are warranted to validate these findings and explore the full potential of XPO1 inhibitors in CTCL treatment.

## Methods

### Reagents, antibodies, cell lines, and human peripheral blood mononuclear cells (PBMCs)

For in vitro studies, we used five CTCL cell lines—MyLa (Millipore Sigma), MJ, Hut78, HH, and H9 (American Type Culture Collection [ATCC]). Cells were cultured in RPMI-1640 medium (Life Technologies) supplemented with 10% fetal calf serum (FCS), 1% GlutaMAX, Antibiotic–Antimycotic, and MJ cells were cultured in Iscove modified Dulbecco medium (IMDM) (Life Technologies) supplemented with 20% FCS, 1% GlutaMAX, and Antibiotic–Antimycotic. Cells were grown at 37 °C in 5% CO_2_. Healthy donor PBMCs were obtained from Leukocyte reduction filters supplied by the Thomas Jefferson University Hospital Blood Bank Laboratory following their standard collection protocols. All the experimental protocols were approved by the Institutional Review Board (17D.083) (Thomas Jefferson University Institutional Review Board). All experiments were performed in accordance with the Declaration of Helsinki. T cells were enriched from PBMCs using the RosetteSep™ Human CD4+ T Cell Enrichment Cocktail according to the manufacturer’s instructions (STEMCELL Technologies).

KPT-330 was purchased from Selleckchem (Selleckchem; TX, USA) and dissolved in DMSO for in vitro studies. KPT-330 was diluted in 0.6% Pluronic® F68 and PVP K-29/32 solution for the in vivo study. Primary antibodies against XPO1, PARP, cleaved-caspase-3, Survivin, p53, p21, p27, GAPDH, Lamin A/C, and β-actin were purchased from Cell Signaling Technology (Cell Signaling; MA, USA). Secondary antibodies (anti-rabbit IgG, HRP-Linked, and anti-mouse IgG, HRP-Linked) were purchased from Cell Signaling Technology (Cell Signaling; MA, USA).

### Quantitative PCR (qPCR) analysis

Total RNA was extracted from cultured cells with Total RNA Purification Plus kit (Norgen Biotek, ON, Canada) as per the manufacturer's protocol. 2 µg of the total RNA was reversely transcribed using SuperScript IV VILO Master Mix (ThermoFisher Scientific, CA, USA). The cDNA obtained was analyzed quantitatively using TaqMan Assay (ThermoFisher Scientific, CA, USA). Ct values were generated using default analysis settings. Relative quantification (RQ) was calculated using the 2^−ΔCT^ method.

### Cell proliferation assay

To assess the chemosensitivity of tumor cells to KPT-330, cell viability was measured by CellTiter 96® Aqueous Non-Radioactive Cell Proliferation Assay (Promega; WI, USA). The cell suspension was cultured in 96-well flat-bottomed microtiter plates at a seeding density of 2 × 10^4^ cells/well. KPT-330 was tested at concentrations ranging from 0.01 to 10.0 µM. Micro titer wells containing lymphoma cells without drug treatment served as controls, and wells containing complete medium served as blank controls. Each dosage was tested in triplicate. Cells were incubated for 72 h before the addition of the assay reagent (1 mg/mL per well), and absorbance was read at 490 nm using a spectrophotometric microplate reader. The percentage cell viability to different drug concentrations was calculated as the inhibition rate of (mean absorbance of treated wells/mean absorbance of control wells) × 100%. IC_50_ was calculated by GraphPad Prism v10.0 (GraphPad Software, Inc; CA, USA).

### Western blot and protein analysis

Cells treated with KPT-330 were washed with ice-cold PBS, resuspended in RIPA lysis buffer (ThermoFisher Scientific; CA, USA) containing protease inhibitor cocktail (ThermoFisher Scientific; CA, USA) and were sonicated for 30 s with 50% pulse. Cell lysates were centrifuged at ∼14,000 × g for 15 min at 4 °C. Protein concentrations were assesed using Bradford assay (Bio-Rad, CA, USA). Nuclear and cytoplasmic fractions were isolated using the NE-PER Kit (ThermoFisher Scientific; CA, USA), followed by separation of protein samples via SDS-PAGE and their subsequent transfer to a PVDF membrane (ThermoFisher Scientific; CA, USA). The membranes were blocked at room temperature for one hour using 5% non-fat dry milk (Bio-Rad; CA, USA) and then incubated overnight at 4 °C with primary antibodies. Following three washes with TBS/Tween 20 (TBST), the membranes were subsequently incubated at room temperature for 1 h with the corresponding anti-rabbit and anti-mouse secondary antibodies, which were conjugated to horseradish peroxidase. The signals were visualized by adding chemiluminescent substrate (ThermoFisher Scientific; CA, USA) followed by quantification by FluroChem auto image system with AlphaView software (Protein Simple; CA, USA).

### Immunofluorescence

Cells were cultured in Nunc™ Lab-Tek™ four well Chamber Slide System (ThermoFisher Scientific; CA, USA) and were exposed to KPT-330 (250 nM) or DMSO (control) for 48 h. Cells were washed in PBS, fixed in 4% paraformaldehyde for 15 min, and permeabilized with 0.1% Triton X-100 for 5 min. Samples were blocked in 5% bovine serum albumin and incubated with primary antibodies overnight at 4 °C. The stained cells were washed thrice with PBS and incubated with Alexa Fluor-conjugated secondary antibodies (Cell Signaling; MA, USA) at room temperature for 1 h in darkness. Following immunostaining, the samples were covered with ProLong® Gold Antifade Reagent with DAPI (Cell Signaling; MA, USA) and coverslips. Images were captured with a BioTek Cytation 5 confocal imaging reader (Agilent Technologies; CA, USA).

### Apoptosis assay

Apoptosis was detected by Annexin V-FITC (fluorescein isothiocyanate) kit (ThermoFisher Scientific; CA, USA) according to the manufacturer's instructions. Briefly, the cells (2 × 10^5^ cells/ml) were cultured in the 6-wells plate in RPMI supplemented with 10% FCS or IMDM supplemented with 20% FCS with either diluents control (DMSO) or varying concentrations of KPT-330 for 48 h. Cells were collected, washed three times with cold PBS, and then suspended in 1× binding buffer. A 100 µl aliquot of the cell suspension was transferred to a microfuge tube and combined with 5 µl of Annexin V-FITC, and then incubated in the dark at room temperature for 15 min. Following this, 200 µl of 1X binding buffer and 5 µl of Propidium Iodide (PI) solution were added to each tube. The cell samples were subsequently examined using a flow cytometer (BD Biosciences, CA, USA) paired with FlowJo software (version 10.8).

### Cell cycle analysis

Cell cycle distribution of cells was analyzed using flow cytometry. To determine the effect of KPT-330 on the cell cycle, lymphoma cells were cultured in the 6-well plate in RPMI supplemented with 10% FCS or IMDM supplemented with 20% FCS with either diluent control (DMSO) or various concentrations of KPT-330 for 48 h. Cells were washed with ice-cold PBS, fixed with ice-cold 70% ethanol at − 20 °C for 2 h, and washed in PBS. Cells were then stained with propidium iodide (PI)/RNase staining buffer (BD Pharmingen™; CA, USA) for 15 min at room temperature in the dark. Cell cycle analysis was performed using a flow cytometer and FlowJo software (version 10.8). A total of 10,000 cells were analyzed for each sample.

### RNA-Seq analysis

The cells (1 × 10^6^ cells/ml) were cultured in a 6-well plate in RPMI supplemented with 10% FCS or IMDM supplemented with 20% FCS. Total RNA was extracted after 48-h treatment with either vehicle (DMSO) or KPT-330 using the Total RNA Purification Plus kit (Norgen Biotek, ON, Canada) as per the manufacturer's protocol. RNA-seq and bioinformatics analysis were conducted at Azenta Life Sciences (New Jersey, USA).

### CTCL xenograft murine model

All in vivo animal experiments were performed in accordance with ARRIVE guidelines. All animal experiments were approved by Thomas Jefferson University Institutional Animal Care and Use Committee (IACUC)(IACUC no. 02234). All experiments were performed in accordance with relevant guidelines and regulations. Female NOD-rag-gamma (NRG) mice (n = 16) 6–8 weeks (The Jackson Laboratory, Bar Harbor, ME) were used in this study. An inspection was performed to ensure their suitability for the study before cancer-cell implantation. As described previously, H9 cells were transfected with PLV-10172[luc/GFP/CMV/Puro] (Cellomics Technology, Halethorpe, MD), and after selection for GFP and confirmation of uniform fluorescence expression^40^, 2 × 10^6^ cells were injected by tail vein into the NRG mice. Mice were then divided into two groups of eight each, with the control group receiving vehicle (DMSO, PEG 300, Tween 80, ddH2O) and the drug group receiving 10 mg/kg KPT-330 via oral gavage starting on Day 4 post-injection three times a week for four weeks. At day 12 and day 21, mice were injected intra-peritoneally with 150 mg/kg D-luciferin (GoldBio, St Louis, MO), and tumor burden was measured via luciferase imaging (IVIS Lumina Series III, PerkinElmer). Images were processed using Living Image software (ver 4.7.3) to determine total flux (photons/second). Mice were euthanized by chemical method using Carbon dioxide (CO_2_) if they had hind limb paralysis or became moribund. The study is reported in accordance with ARRIVE guidelines.

### Statistical analysis

Statistical analyses were performed with GraphPad Prism v10.0 (GraphPad Software, Inc; CA, USA). Student's t test was performed between two groups; data are recorded in the form of mean ± SD. *P* values of < 0.05 were considered statistically significant (*p* < 0.05: *, *p* < 0.01: **, *p* < 0.001: ***). We also use the Kaplan–Meier test for the univariate survival analysis.

### Supplementary Information

Supplementary Figure S1.

Supplementary Information.

## Data Availability

The datasets generated and analysed during the current study are available in the Gene Expression Omnibus (GEO) database under the accession number GSE262491.

## References

[1] Gorlich D, Mattaj IW (1996). Nucleocytoplasmic transport. Science.

[2] Hanahan D (2022). Hallmarks of cancer: New dimensions. Cancer Discov..

[3] Cagatay T, Chook YM (2018). Karyopherins in cancer. Curr. Opin. Cell Biol..

[4] Azizian NG, Li Y (2020). XPO1-dependent nuclear export as a target for cancer therapy. J. Hematol. Oncol..

[5] Azmi AS (2017). Exportin 1 (XPO1) inhibition leads to restoration of tumor suppressor miR-145 and consequent suppression of pancreatic cancer cell proliferation and migration. Oncotarget.

[6] Nachmias B, Schimmer AD (2020). Targeting nuclear import and export in hematological malignancies. Leukemia.

[7] Dummer R (2021). Cutaneous T cell lymphoma. Nat. Rev. Dis. Primers.

[8] Ishikawa C, Mori N (2022). Exportin-1 is critical for cell proliferation and survival in adult T cell leukemia. Invest. New Drugs.

[9] Nie D (2022). Prognostic and therapeutic significance of XPO1 in T-cell lymphoma. Exp. Cell Res..

[10] Turner JG, Dawson J, Sullivan DM (2012). Nuclear export of proteins and drug resistance in cancer. Biochem. Pharmacol..

[11] Wang AY, Liu H (2019). The past, present, and future of CRM1/XPO1 inhibitors. Stem Cell Investig.

[12] Sun Q (2016). Inhibiting cancer cell hallmark features through nuclear export inhibition. Signal Transduct. Target Ther.

[13] Azmi AS, Uddin MH, Mohammad RM (2021). The nuclear export protein XPO1—From biology to targeted therapy. Nat. Rev. Cli.n Oncol..

[14] Landes JR (2022). The efficacy of selinexor (KPT-330), an XPO1 inhibitor, on non-hematologic cancers: A comprehensive review. J. Cancer Res. Clin. Oncol..

[15] Sochacka-Cwikla A, Maczynski M, Regiec A (2021). FDA-approved drugs for hematological malignancies-the last decade review. Cancers (Basel).

[16] Kanehisa M, Furumichi M, Sato Y, Kawashima M, Ishiguro-Watanabe M (2023). KEGG for taxonomy-based analysis of pathways and genomes. Nucleic Acids Res..

[17] Kanehisa M, Goto S (2000). KEGG: Kyoto encyclopedia of genes and genomes. Nucleic Acids Res..

[18] Jain S (2015). Preclinical pharmacologic evaluation of pralatrexate and romidepsin confirms potent synergy of the combination in a murine model of human T-cell lymphoma. Clin. Cancer Res..

[19] Handley KF (2021). Rational combination of CRM1 inhibitor selinexor and olaparib shows synergy in ovarian cancer cell lines and mouse models. Mol. Cancer Ther..

[20] Seymour EK (2021). Selinexor in combination with R-CHOP for frontline treatment of non-hodgkin lymphoma: Results of a phase I study. Clin. Cancer Res..

[21] Zhu ZC, Liu JW, Yang C, Zhao M, Xiong ZQ (2019). XPO1 inhibitor KPT-330 synergizes with Bcl-xL inhibitor to induce cancer cell apoptosis by perturbing rRNA processing and Mcl-1 protein synthesis. Cell Death Dis..

[22] Scheijen B, Ngo HT, Kang H, Griffin JD (2004). FLT3 receptors with internal tandem duplications promote cell viability and proliferation by signaling through Foxo proteins. Oncogene.

[23] Balasubramanian SK, Azmi AS, Maciejewski J (2022). Selective inhibition of nuclear export: A promising approach in the shifting treatment paradigms for hematological neoplasms. Leukemia.

[24] Talaat IM (2019). Potential role for microRNA-16 (miR-16) and microRNA-93 (miR-93) in diagnosis and prediction of disease progression in mycosis fungoides in Egyptian patients. PLoS One.

[25] Zhang C (2010). Curcumin selectively induces apoptosis in cutaneous T-cell lymphoma cell lines and patients' PBMCs: Potential role for STAT-3 and NF-kappaB signaling. J. Invest. Dermatol..

[26] Knauer SK, Bier C, Habtemichael N, Stauber RH (2006). The Survivin-Crm1 interaction is essential for chromosomal passenger complex localization and function. EMBO Rep..

[27] Altieri DC (2003). Validating survivin as a cancer therapeutic target. Nat. Rev. Cancer.

[28] Altieri DC (2008). Survivin, cancer networks and pathway-directed drug discovery. Nat. Rev. Cancer.

[29] Altieri DC (2013). Targeting survivin in cancer. Cancer Lett..

[30] Zhang C (2008). Avicin D selectively induces apoptosis and downregulates p-STAT-3, bcl-2, and survivin in cutaneous T-cell lymphoma cells. J. Invest. Dermatol..

[31] Li G (1998). Overexpression of p53 protein in cutaneous T cell lymphoma: Relationship to large cell transformation and disease progression. J. Invest. Dermatol.

[32] Hu J (2021). Targeting mutant p53 for cancer therapy: direct and indirect strategies. J. Hematol. Oncol..

[33] Baeten JT, Chan ICC, Link DC, Bolton KL (2021). Effects of PARP inhibitor therapy on p53-deficient hematopoietic stem and progenitor cell fitness. Blood.

[34] Jemaa M (2012). Selective killing of p53-deficient cancer cells by SP600125. EMBO Mol. Med..

[35] Li T (2012). Tumor suppression in the absence of p53-mediated cell-cycle arrest, apoptosis, and senescence. Cell.

[36] Netchiporouk E (2017). Analysis of CTCL cell lines reveals important differences between mycosis fungoides/Sezary syndrome versus HTLV-1(+) leukemic cell lines. Oncotarget.

[37] Cmielova J, Rezacova M (2011). p21Cip1/Waf1 protein and its function based on a subcellular localization [corrected]. J. Cell Biochem..

[38] Boons E (2021). XPO1 inhibitors represent a novel therapeutic option in Adult T-cell Leukemia, triggering p53-mediated caspase-dependent apoptosis. Blood Cancer J..

[39] Ding K (2023). High rates of remission with the initial treatment of oral selinexor plus CHOP for newly diagnosed peripheral T-cell lymphoma (PTCL): Clinical outcomes analysis of a multi-center phase II study in China. Blood.

[40] Isabelle C (2023). Preclinical evaluation of anti-CD38 therapy in mature T-cell neoplasms. Blood Adv..

