# Supplementary material for: XPO1 blockade with KPT-330 promotes apoptosis in cutaneous T-cell lymphoma by activating the p53–p21 and p27 pathways
Source: Sci Rep. 2024 Apr 23;14:9305. doi: 10.1038/s41598-024-59994-5 (PMC11039474; doi:10.1038/s41598-024-59994-5)
Supplement: Supplementary file 1 — Supplementary Figure S1. [file 41598_2024_59994_MOESM1_ESM.pdf]

XPO1 blockade with KPT-330 promotes apoptosis in Cutaneous T- cell Lymphoma by activating the p53-p21 and p27 pathways

Nitin Chakravarti*, Amy Boles, Rachel Burzinski, Paola Sindaco, Colleen Isabelle, Kathleen McConnell, Anjali Mishra, and Pierluigi Porcu*

Supplementary Figure S1

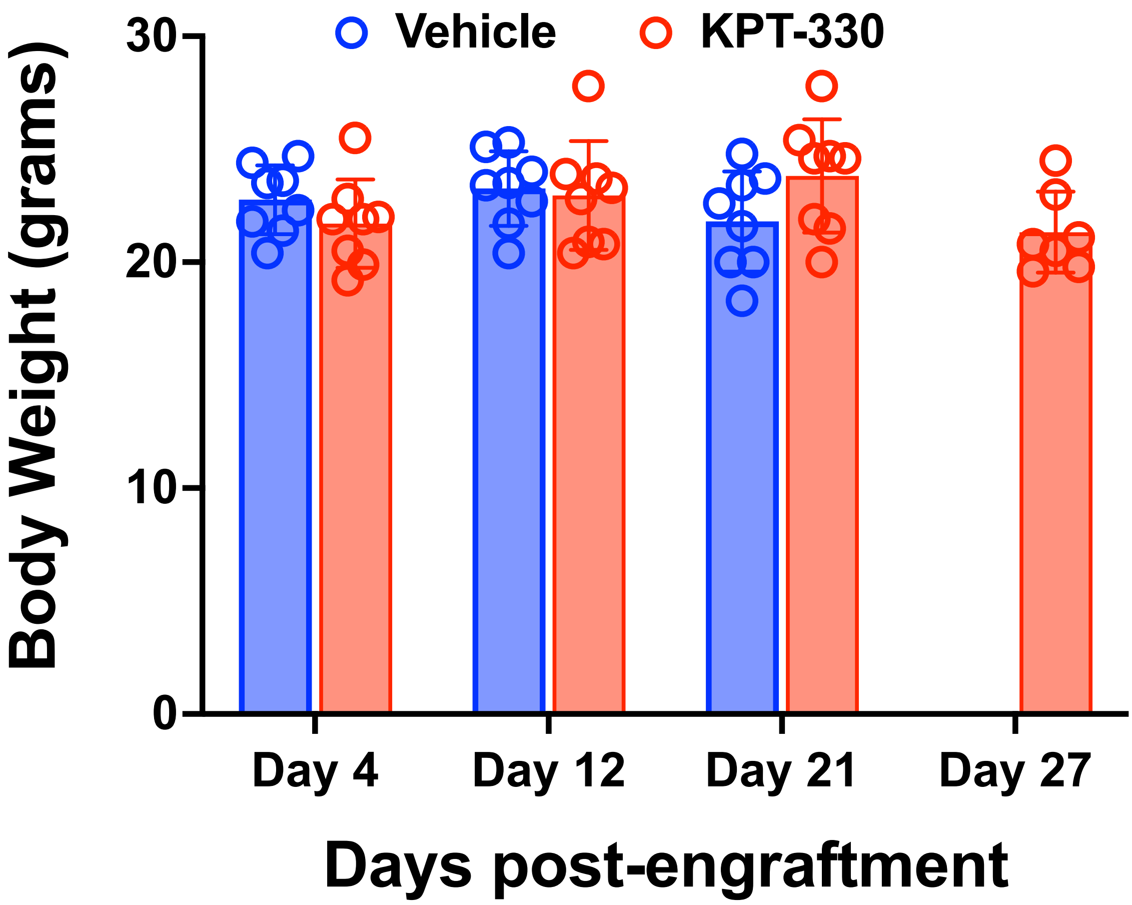

Supplementary Figure Legend

Supplementary Figure S1: Pre-clinical efficacy of KPT-330 in mice xenograft models – **(A)** Relative body weight curves of NSG mice injected with H9-Luc-GFP cells treated with 10mg/kg KPT-330 (mean ± SEM, n = 8, red bar). Untreated mice injected with H9-Luc-GFP cells (mean ± SEM, n = 8, blue bar) were used as controls.
