# Supplementary material for: XPO1 blockade with KPT-330 promotes apoptosis in cutaneous T-cell lymphoma by activating the p53–p21 and p27 pathways
Source: Sci Rep. 2024 Apr 23;14:9305. doi: 10.1038/s41598-024-59994-5 (PMC11039474; doi:10.1038/s41598-024-59994-5)

XPO1 blockade with KPT-330 promotes apoptosis in Cutaneous T- cell Lymphoma by activating the p53-p21 and p27 pathways

Nitin Chakravarti*, Amy Boles, Rachel Burzinski, Paola Sindaco, Colleen Isabelle, Kathleen McConnell, Anjali Mishra, and Pierluigi Porcu*

Figure 1A

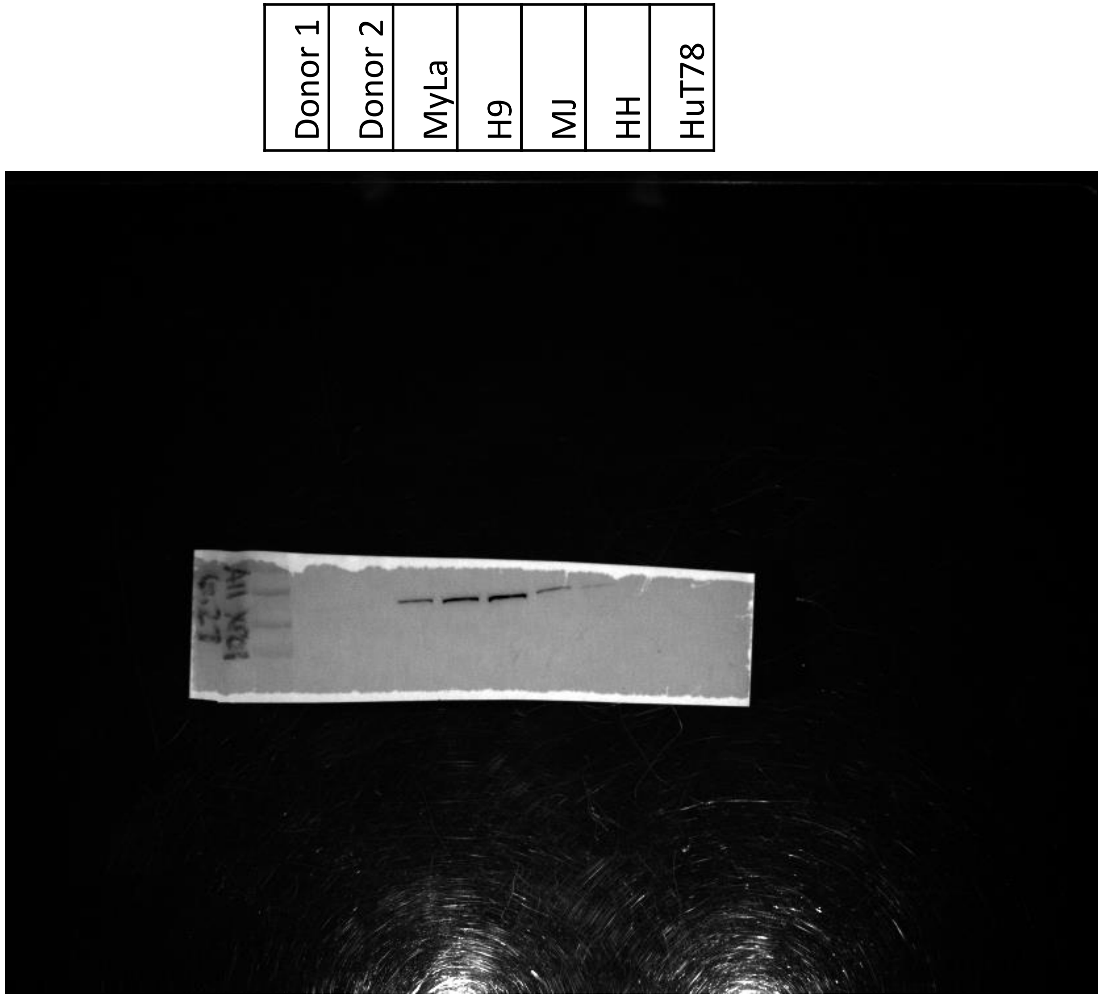

XPO1

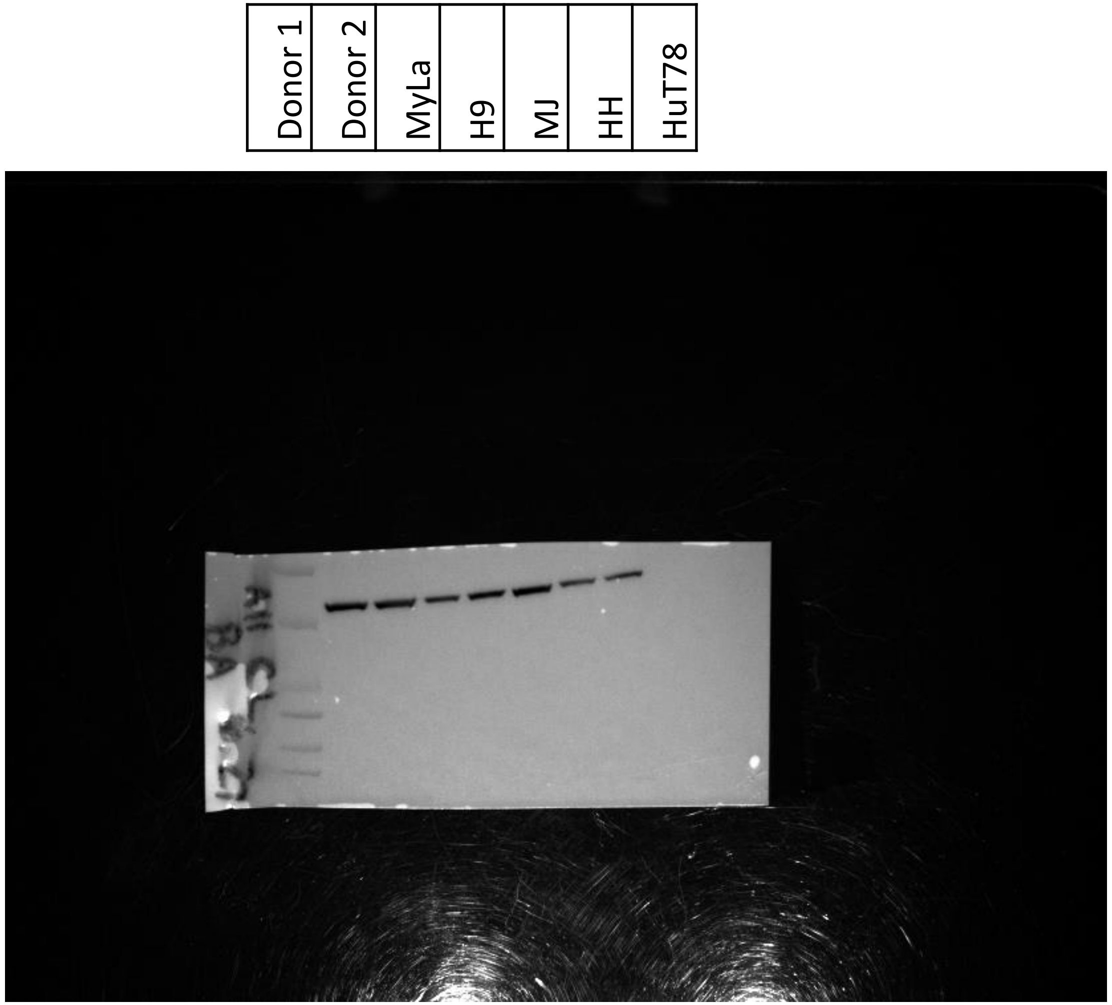

β-actin

Figure 1D –MJ XPO1

Ctrl	25 nM	50 nM	100 nM	250 nM	500 nM

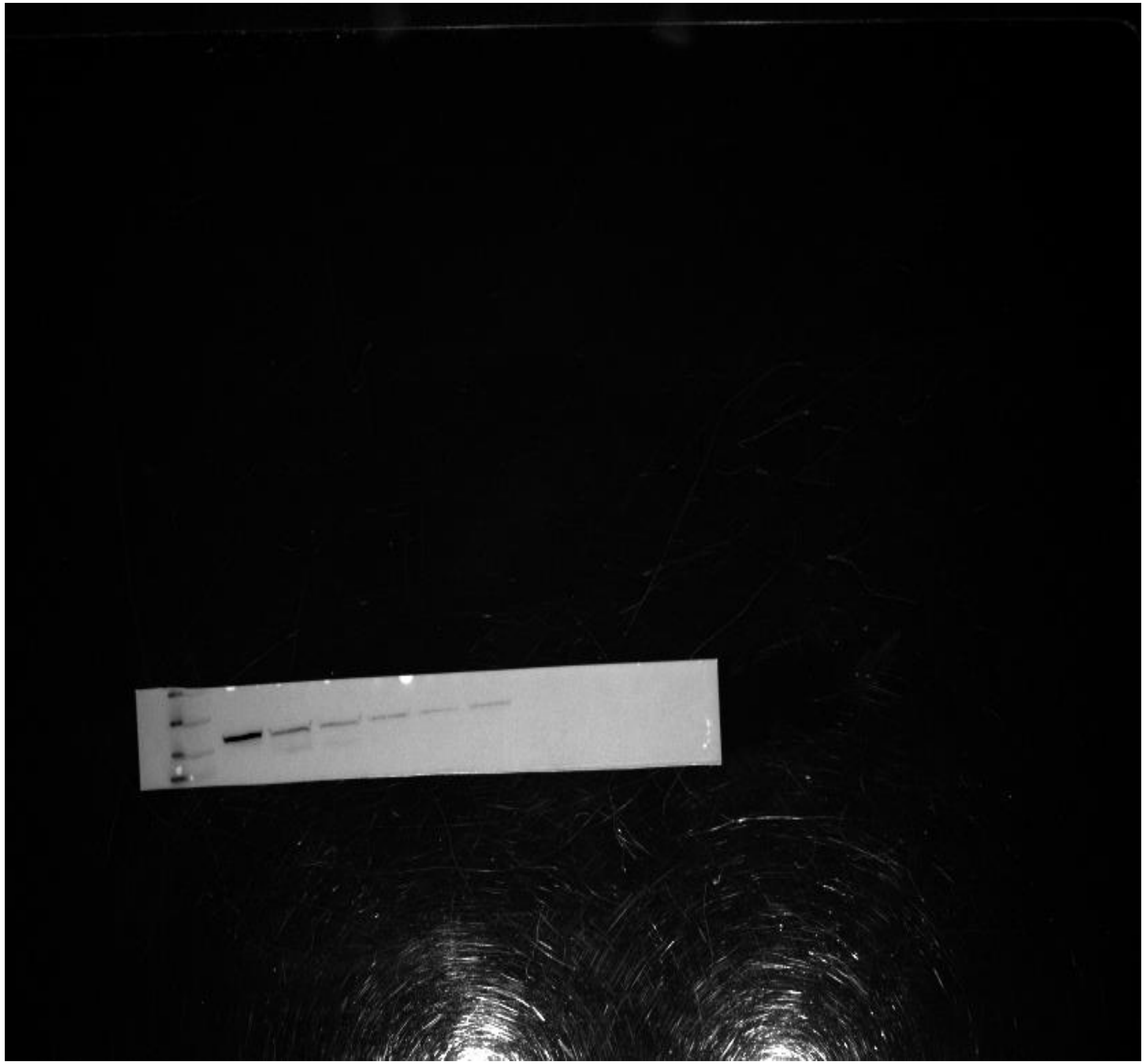

XPO1

Ctrl	25 nM	50 nM	100 nM	250 nM	500 nM

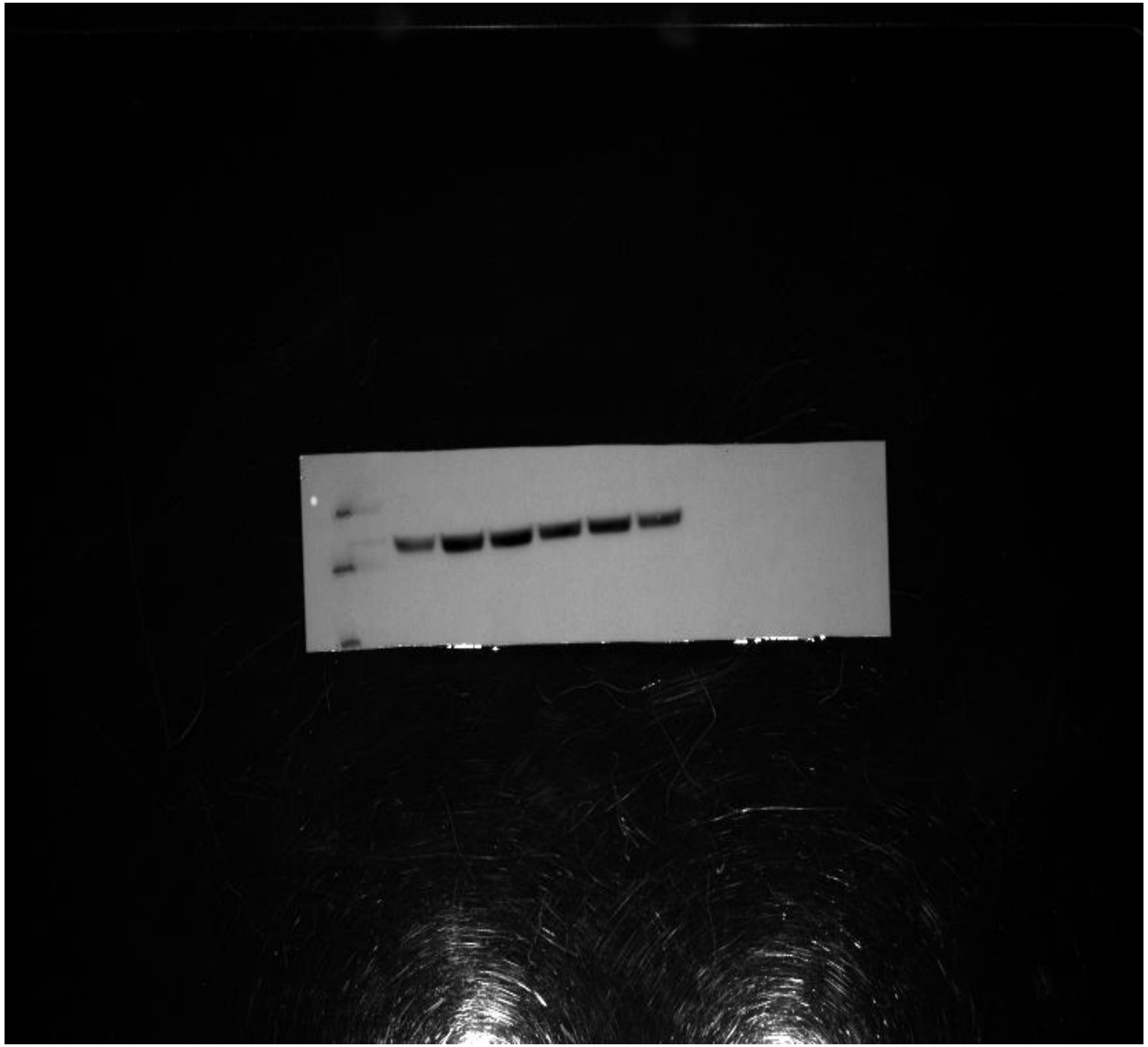

β-actin

Figure 1D –MyLa XPO1

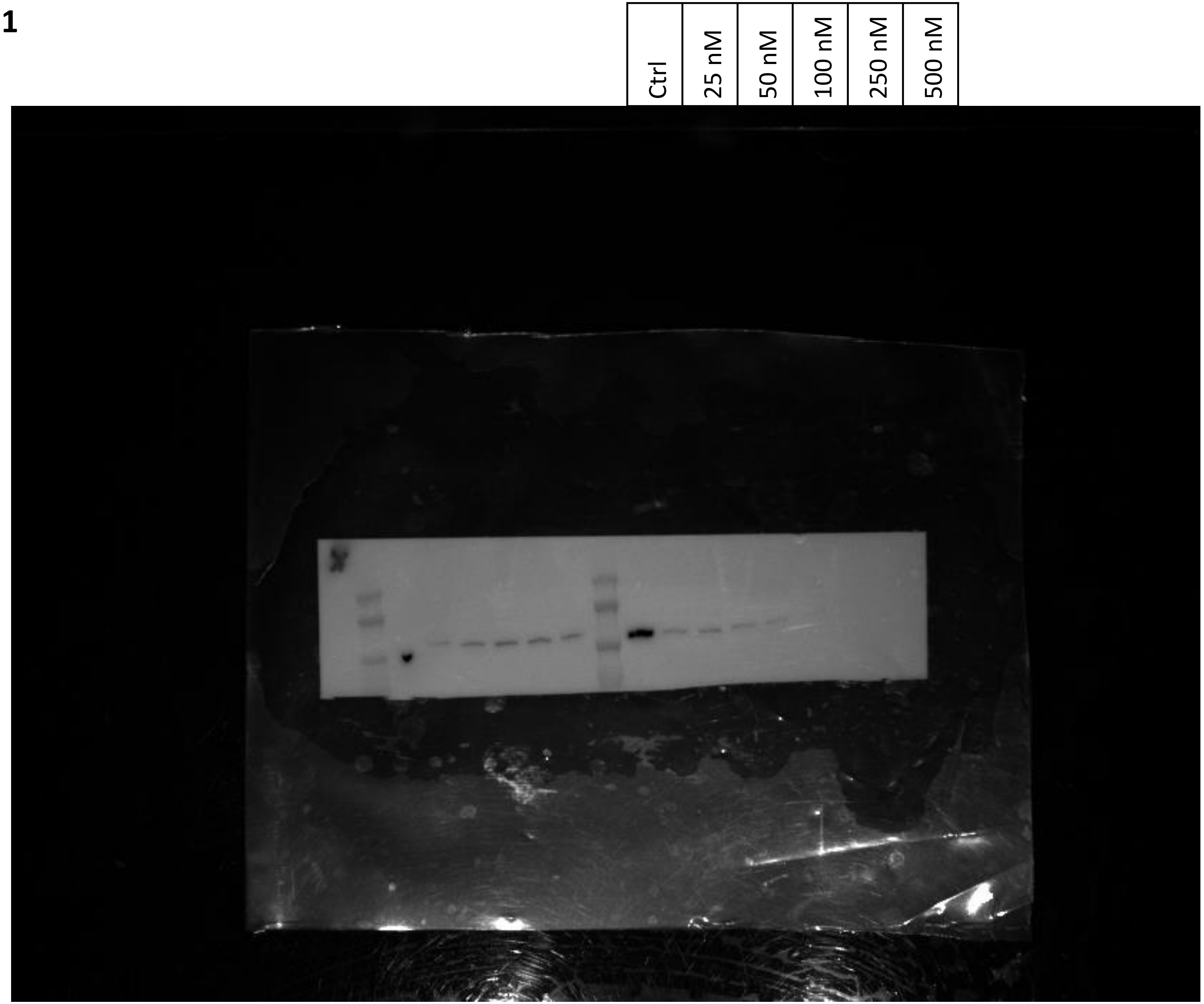

Figure 1D –MyLa β-actin

Ctrl	25 nM	50 nM	100 nM	250 nM	500 nM

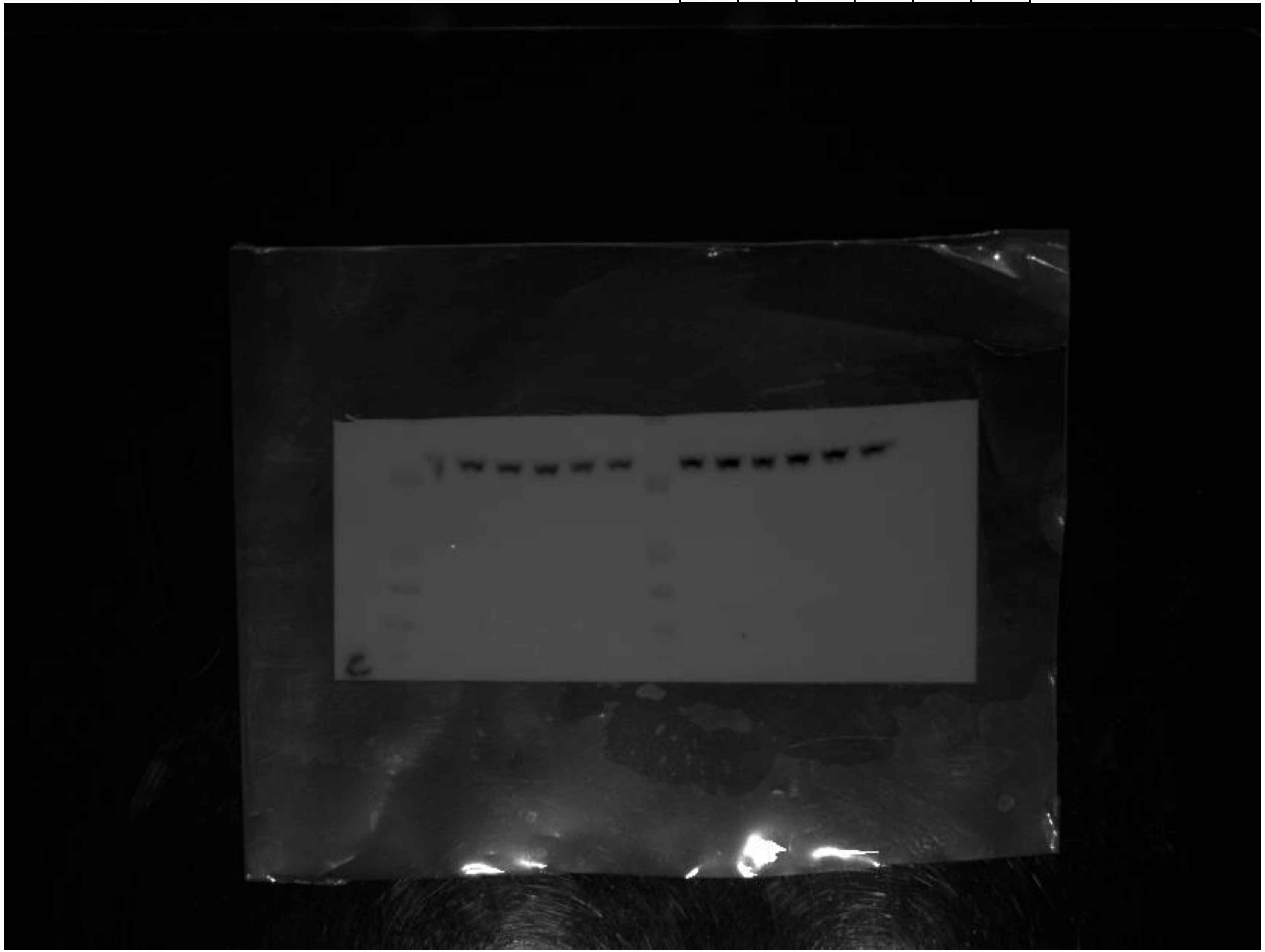

Figure 1D –H9 XPO1

Ctrl	25 nM	50 nM	100 nM	250 nM	500 nM

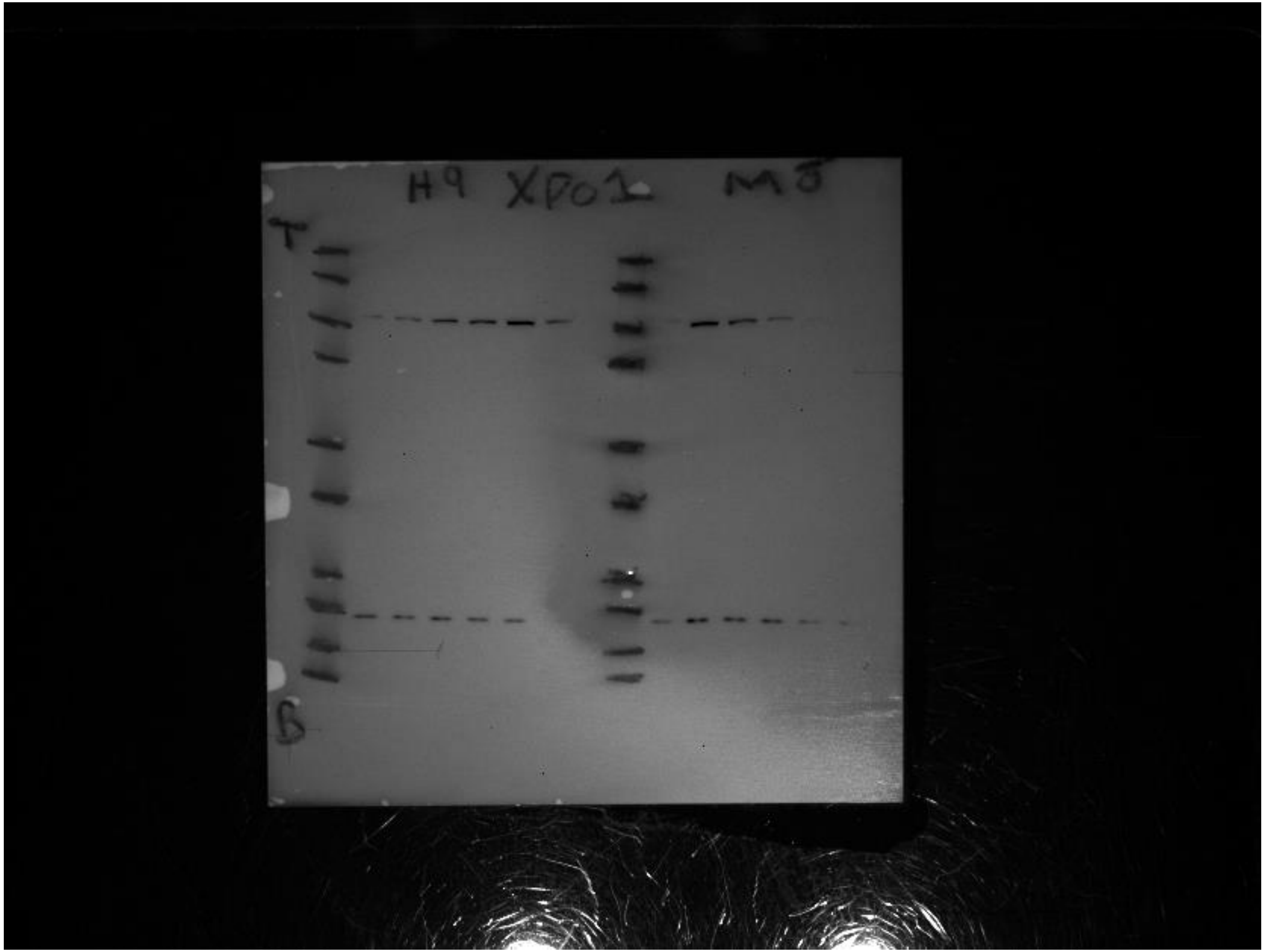

Figure 1D –H9 β-actin

Ctrl	25 nM	50 nM	100 nM	250 nM	500 nM

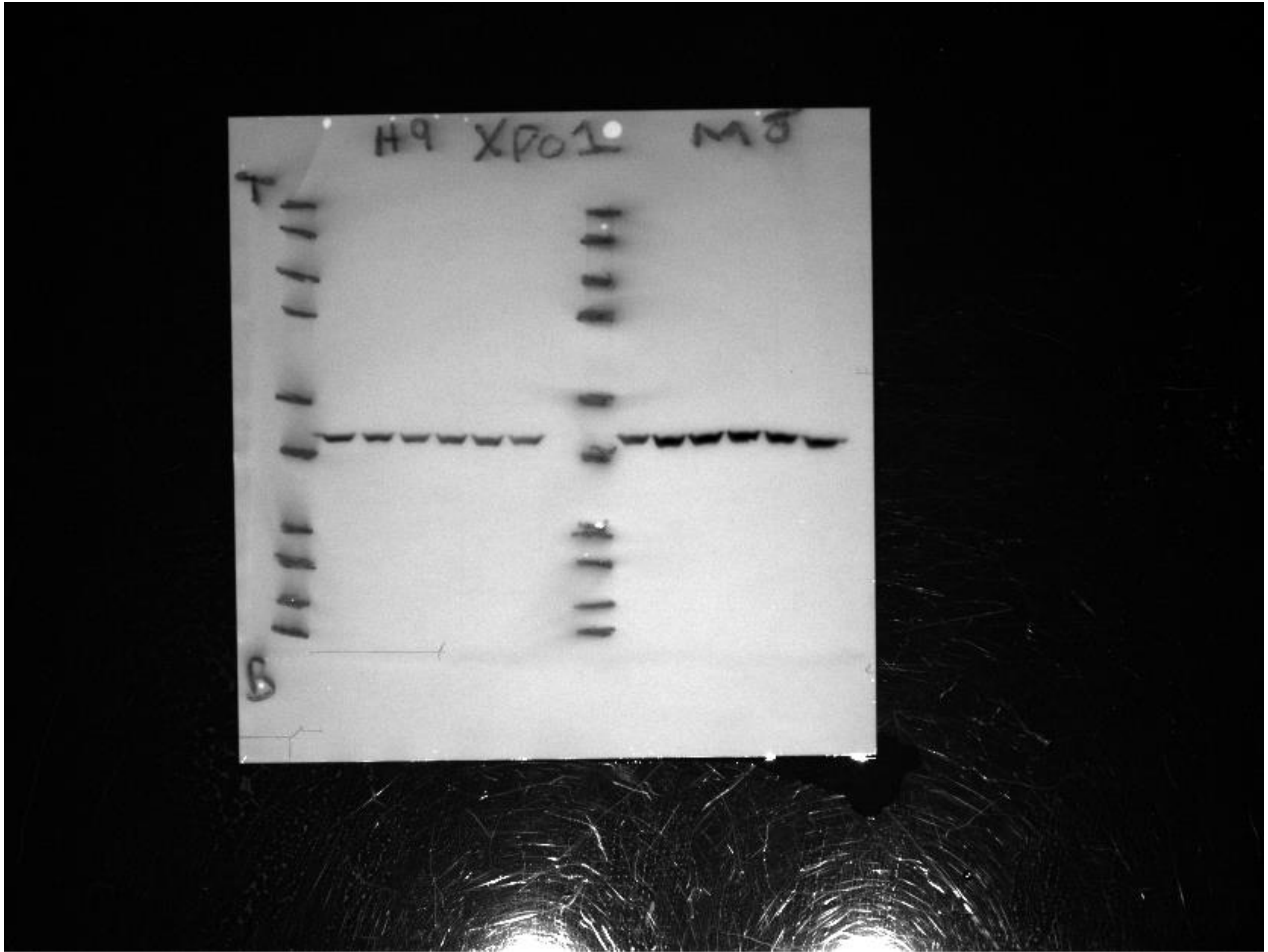

Figure 1E –MJ XPO1

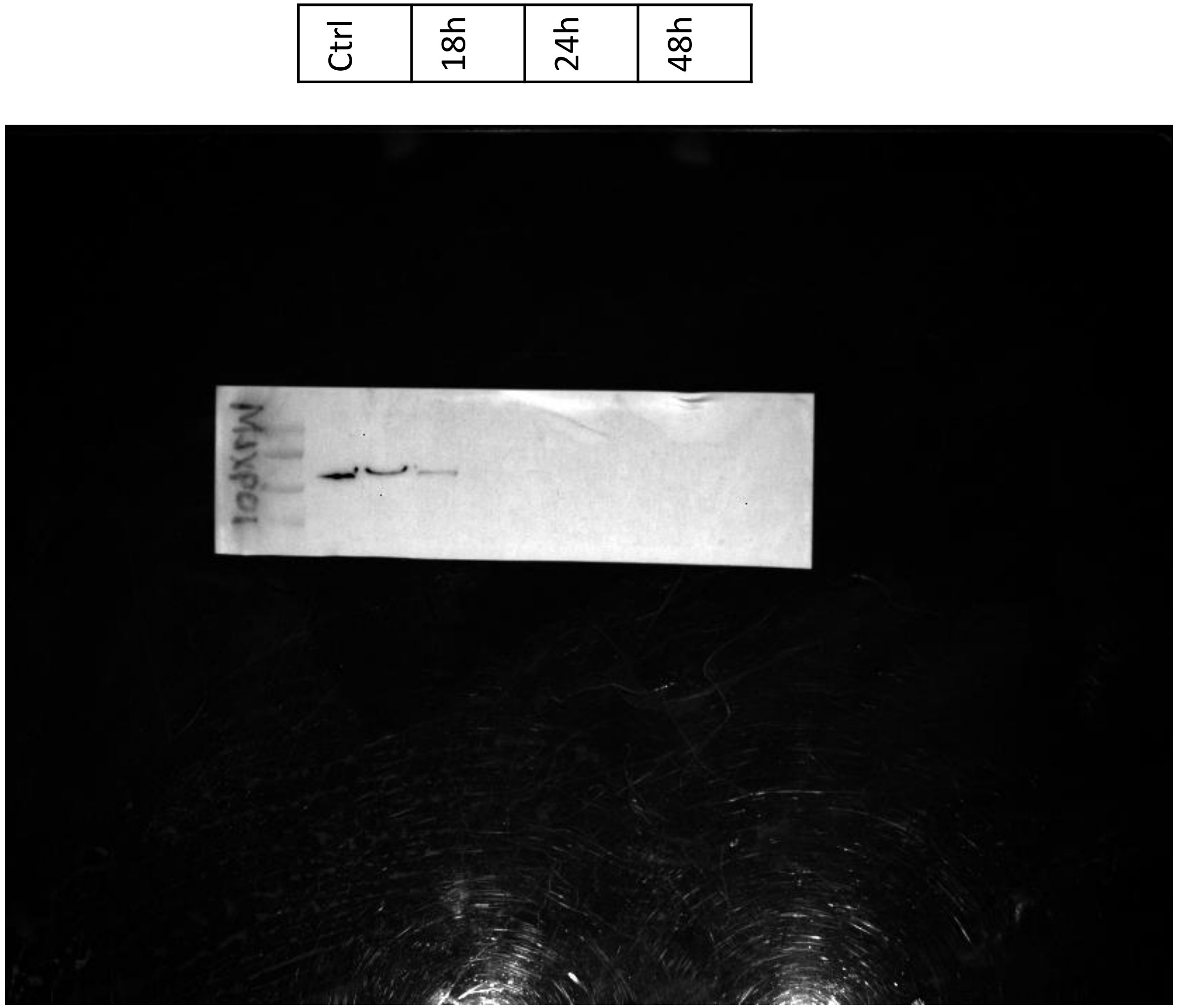

XPO1

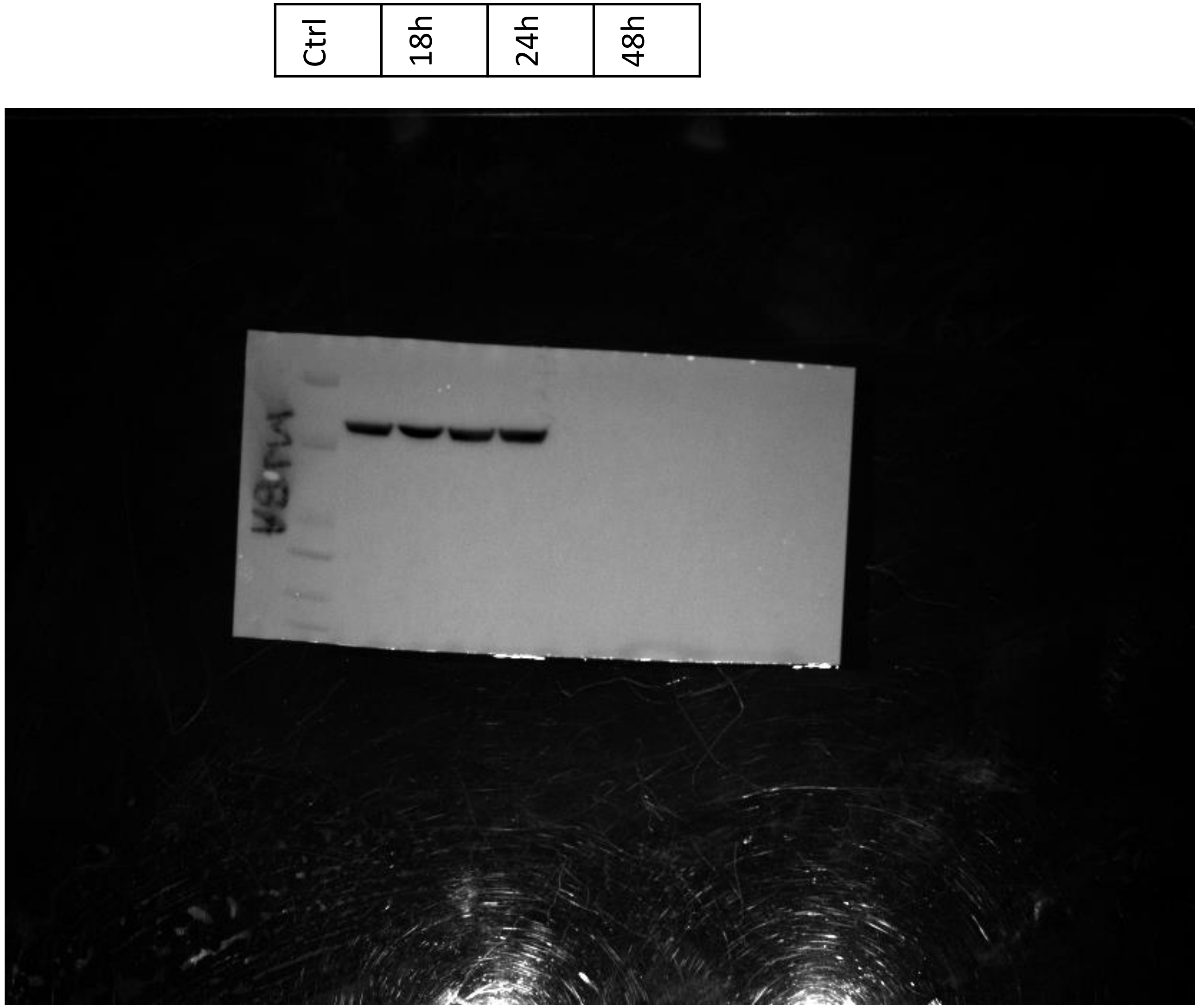

β-actin

Figure 1E –MyLa XPO1

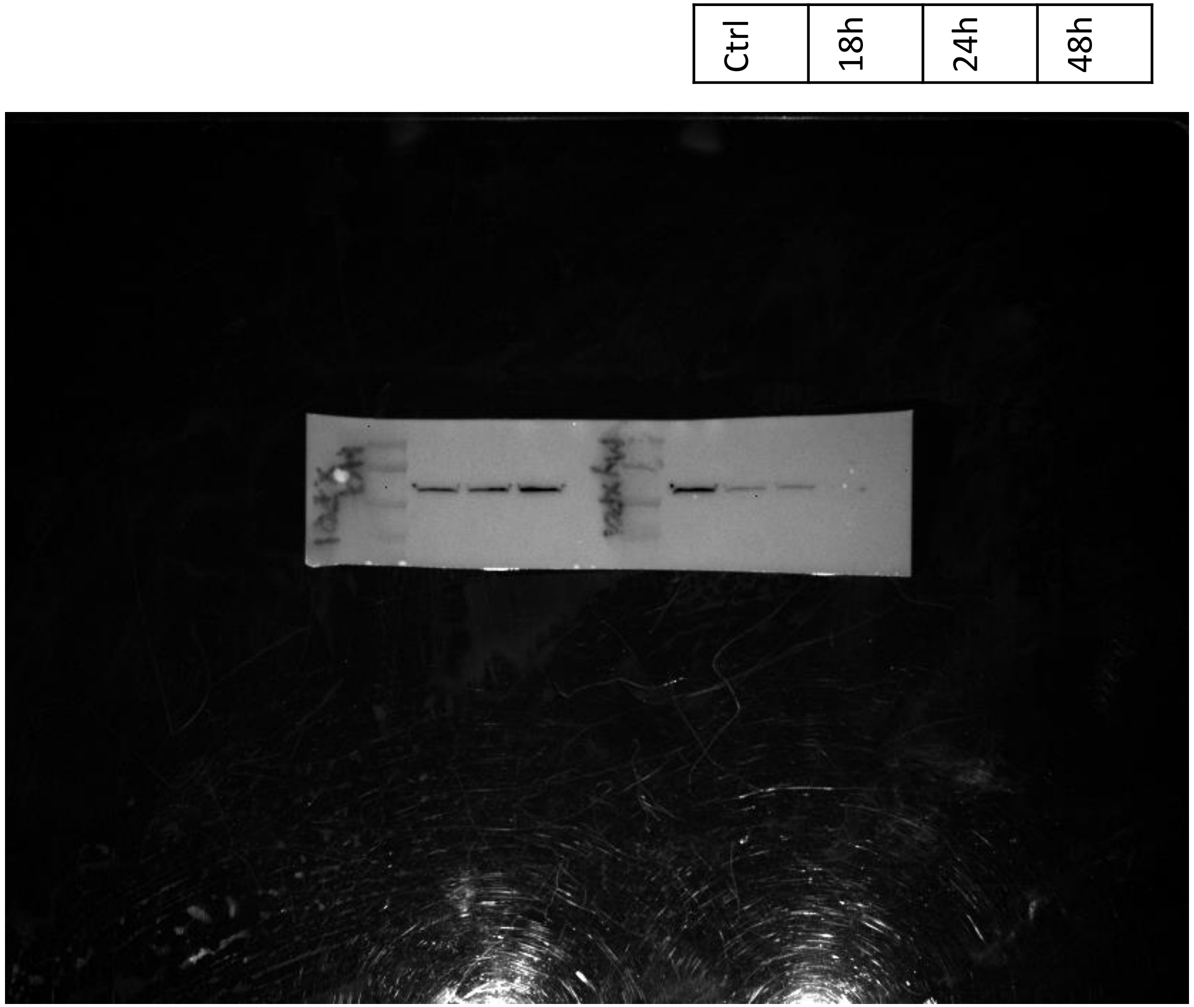

XPO1

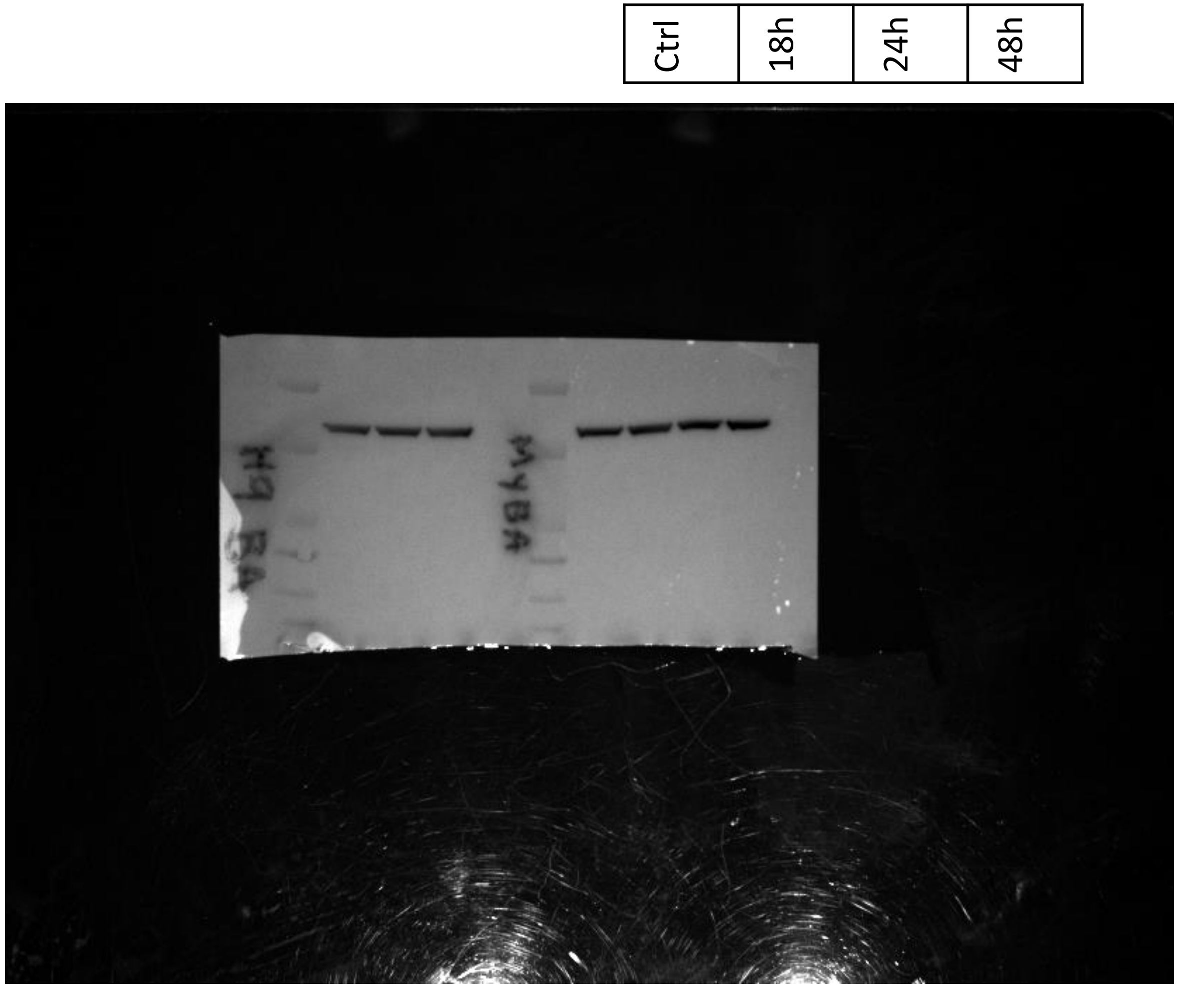

β-actin

Figure 1E –H9 XPO1

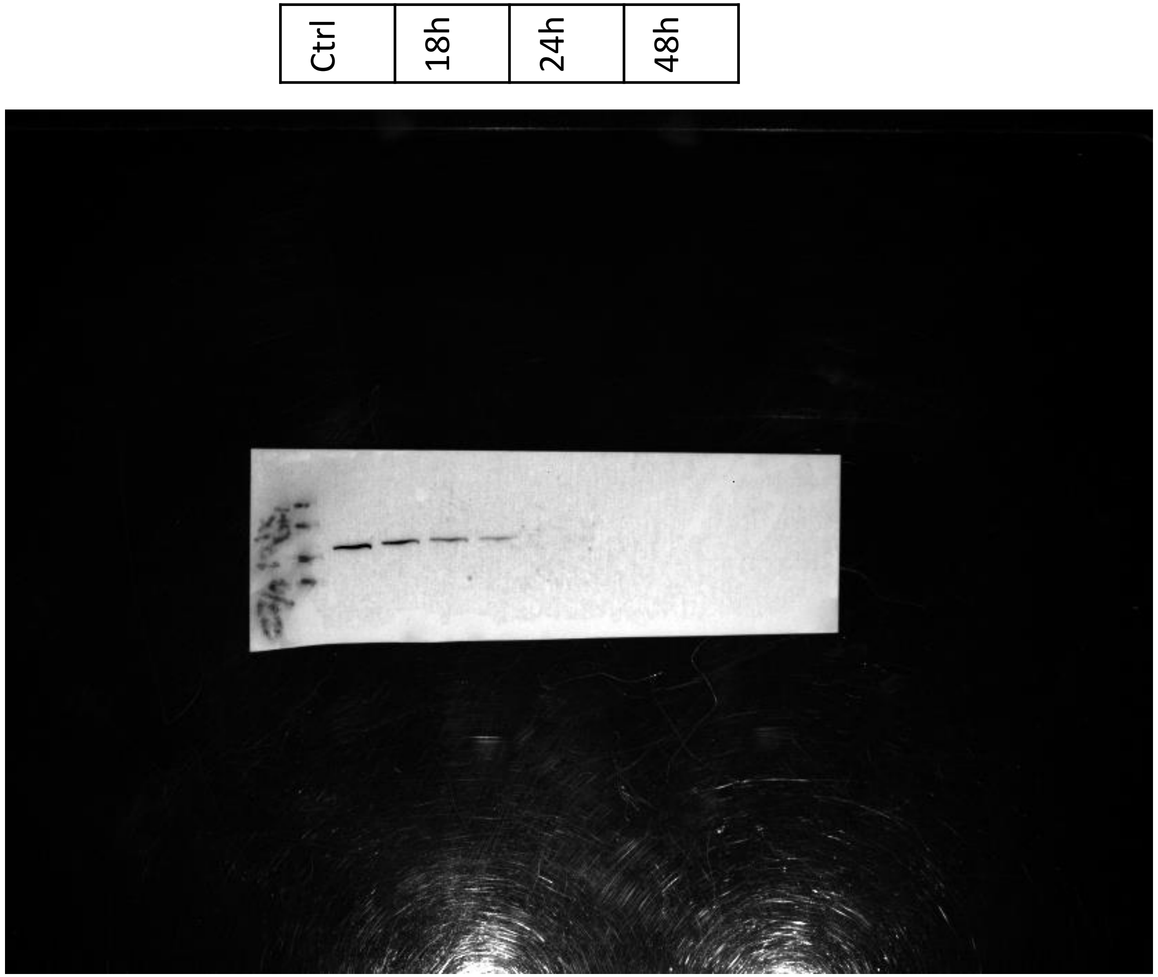

XPO1

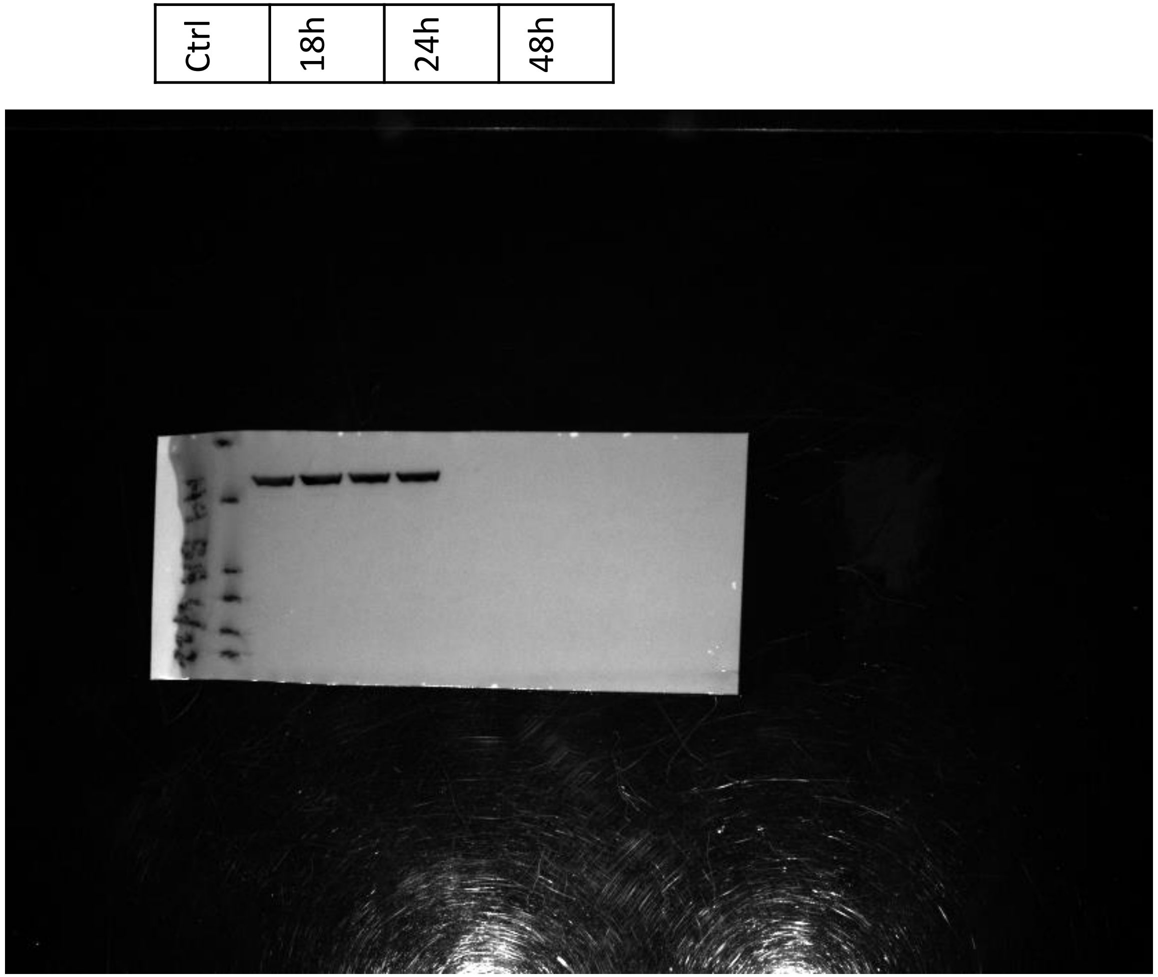

β-actin

Figure 3C –MJ- p53

Ctrl	25 nM	50 nM	100 nM	250 nM	500 nM

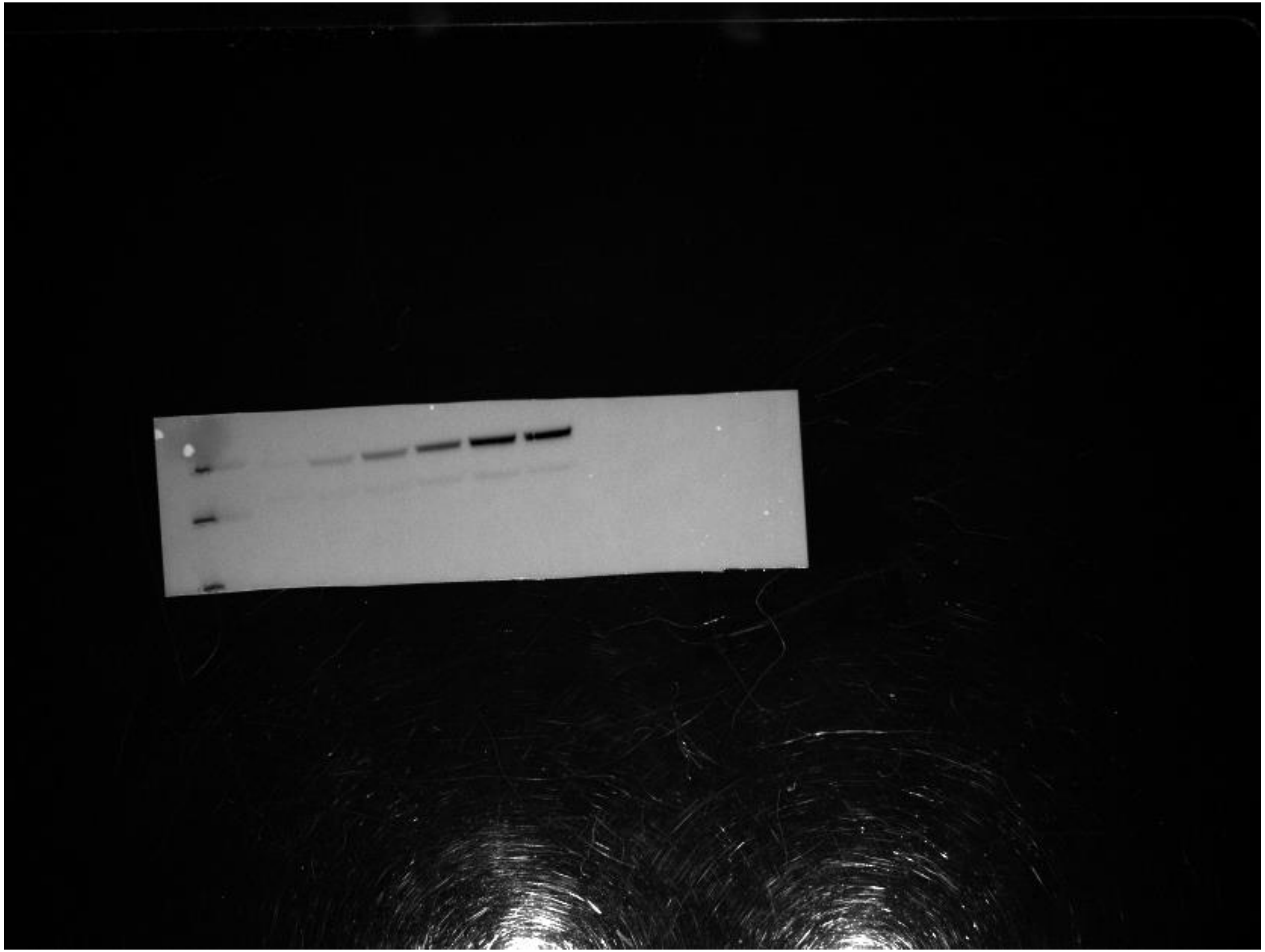

Figure 3C –MJ- p21

Ctrl	25 nM	50 nM	100 nM	250 nM	500 nM

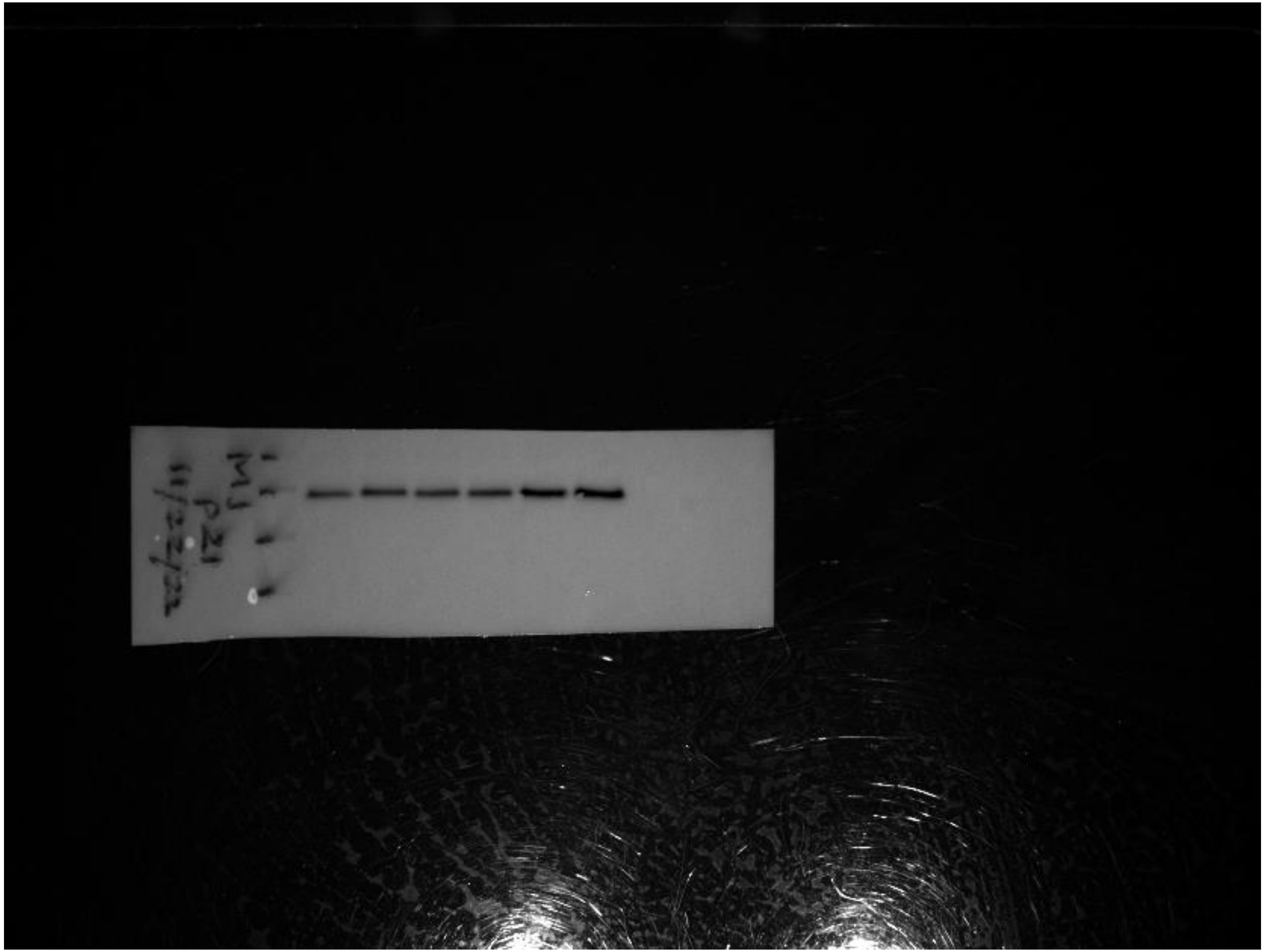

Figure 3C –MJ- p27

Ctrl	25 nM	50 nM	100 nM	250 nM	500 nM

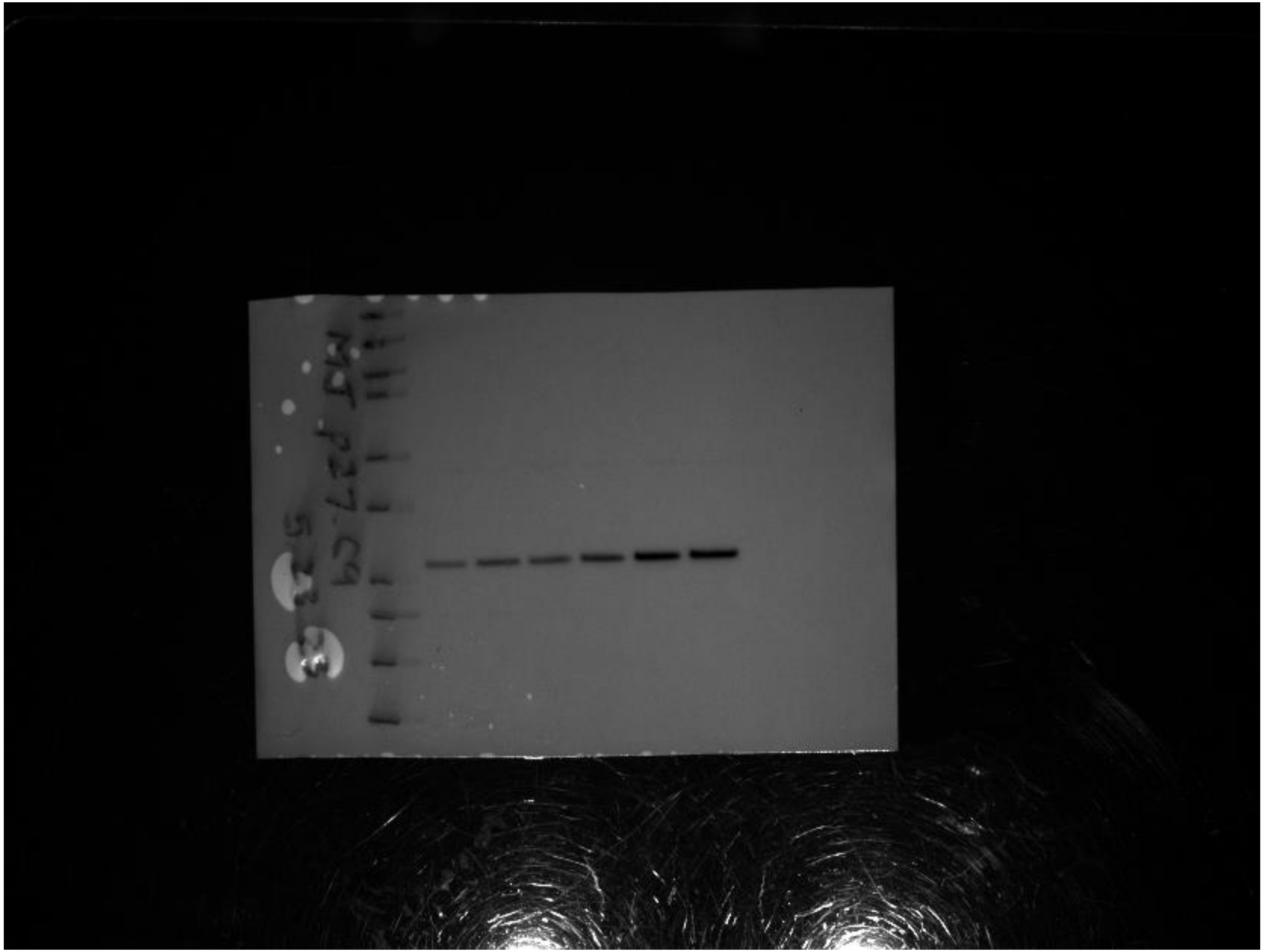

Figure 3C –MJ- Survivin

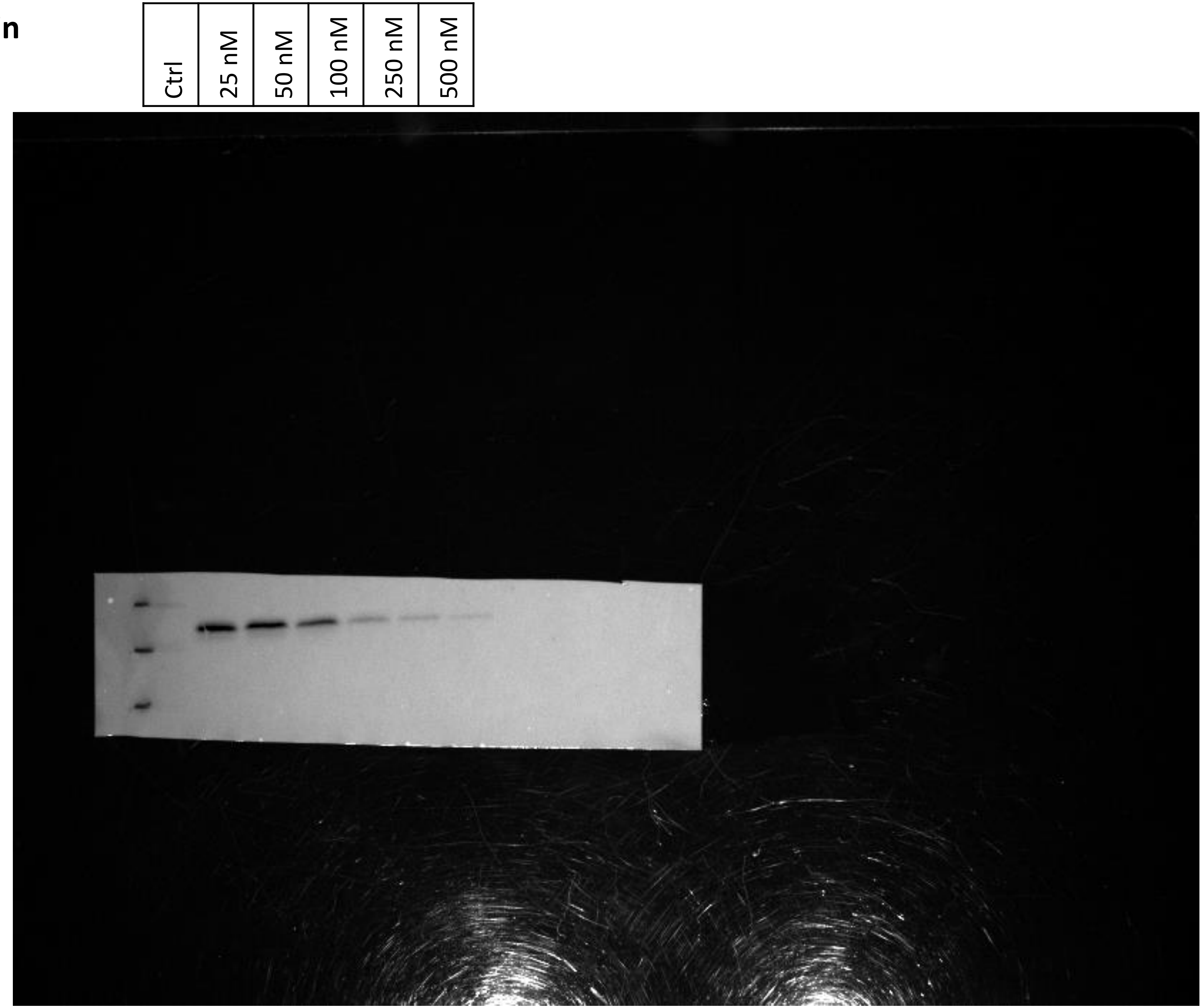

Figure 3C –H9 and MJ- cCASP3

H9 →MJ

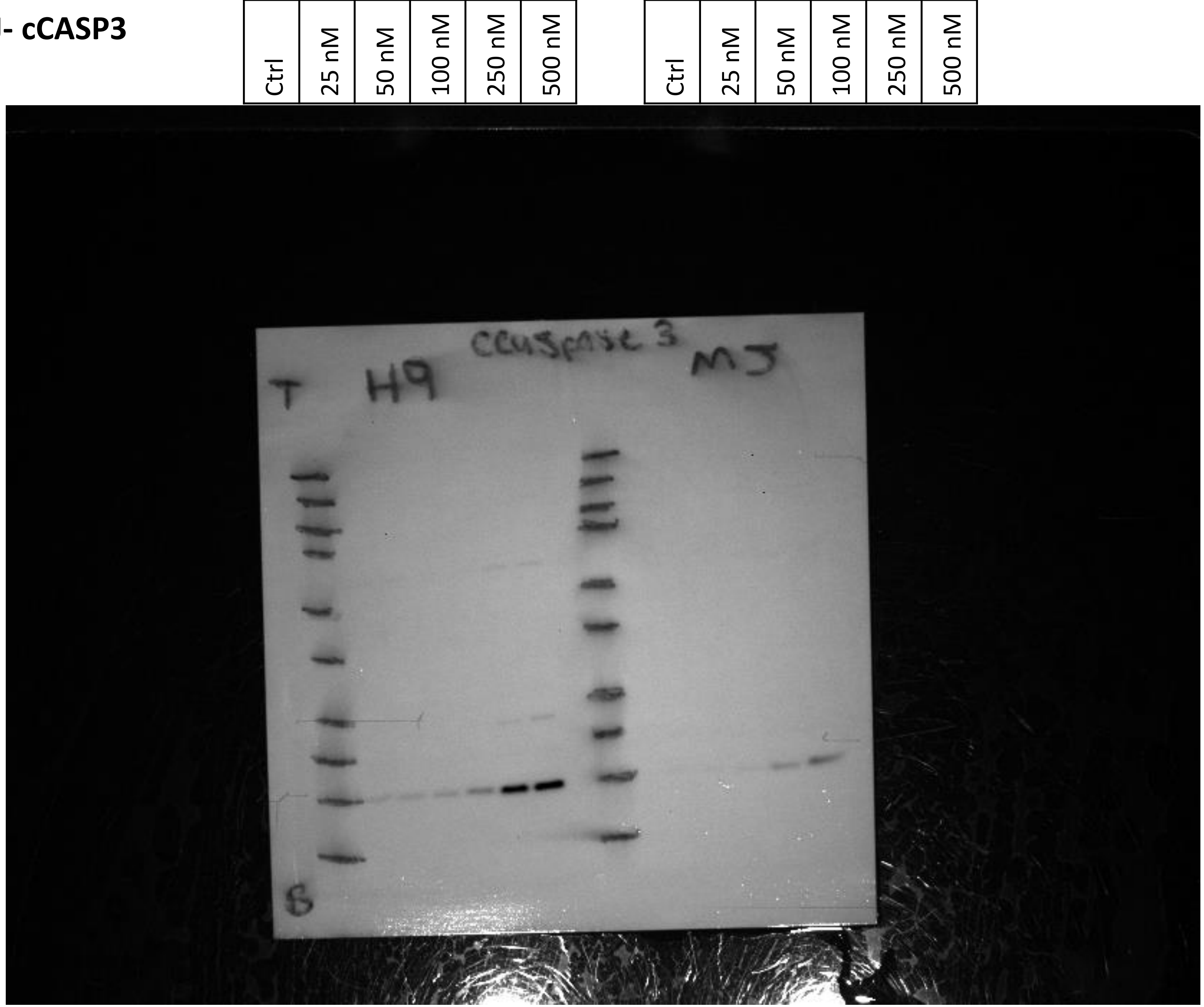

Figure 3C –MJ- PARP

Ctrl	25 nM	50 nM	100 nM	250 nM	500 nM

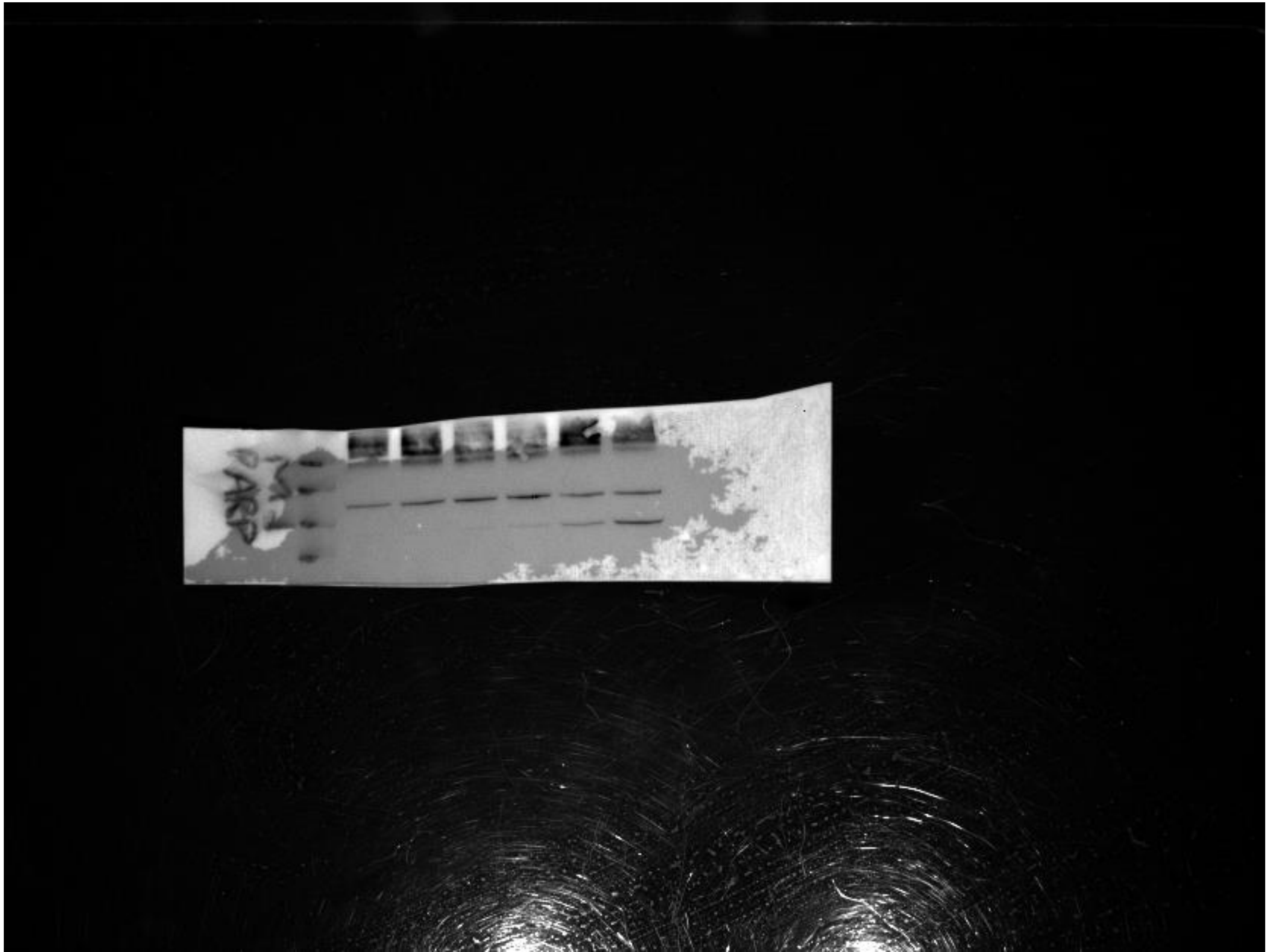

Figure 3C –MJ-B-actin

Ctrl	25 nM	50 nM	100 nM	250 nM	500 nM

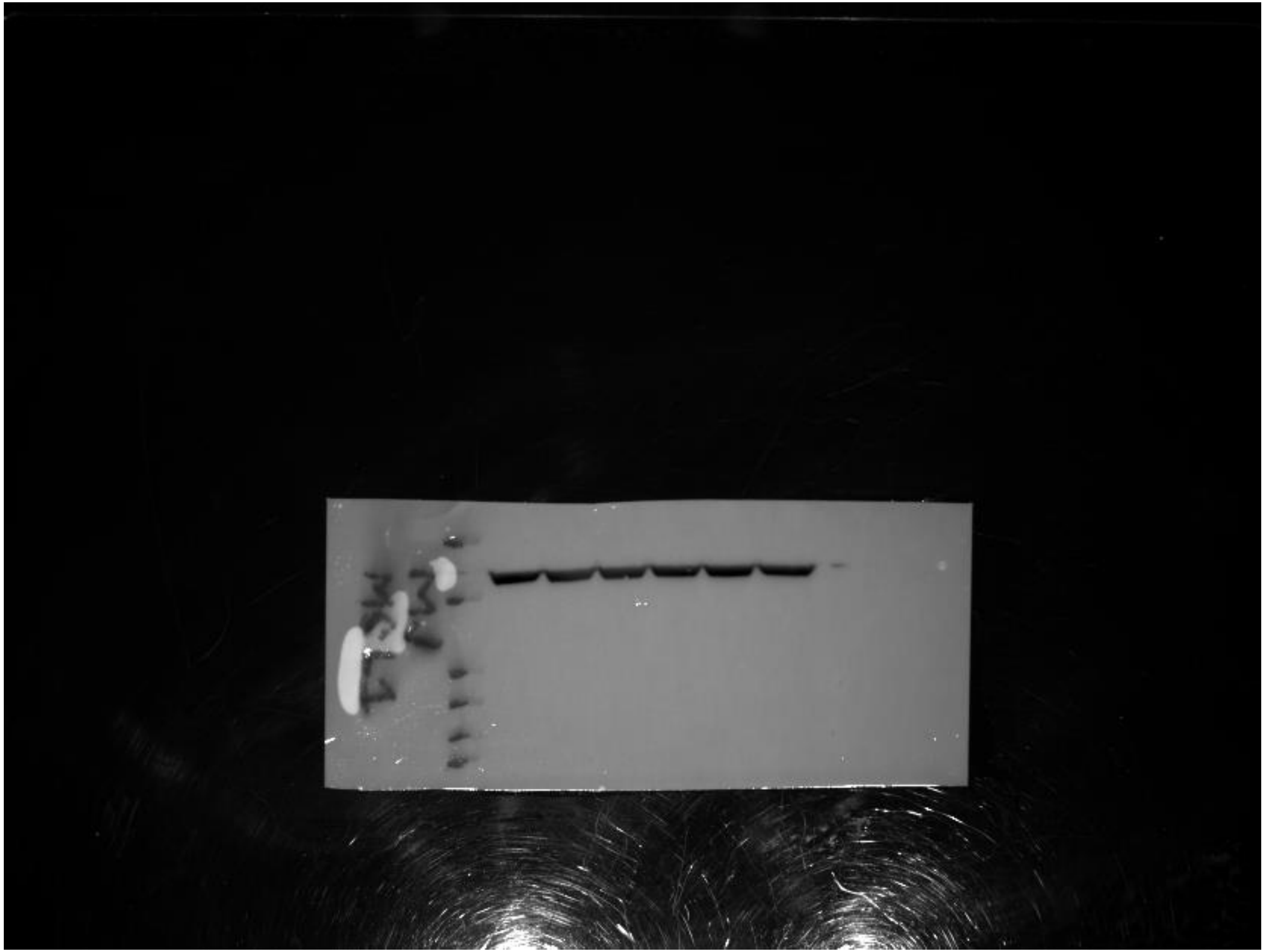

Figure 3C –MyLa and H9- p53

Ctrl	25 nM	50 nM	100 nM	250 nM	500 nM

Ctrl	25 nM	50 nM	100 nM	250 nM	500 nM

H9→MyLa

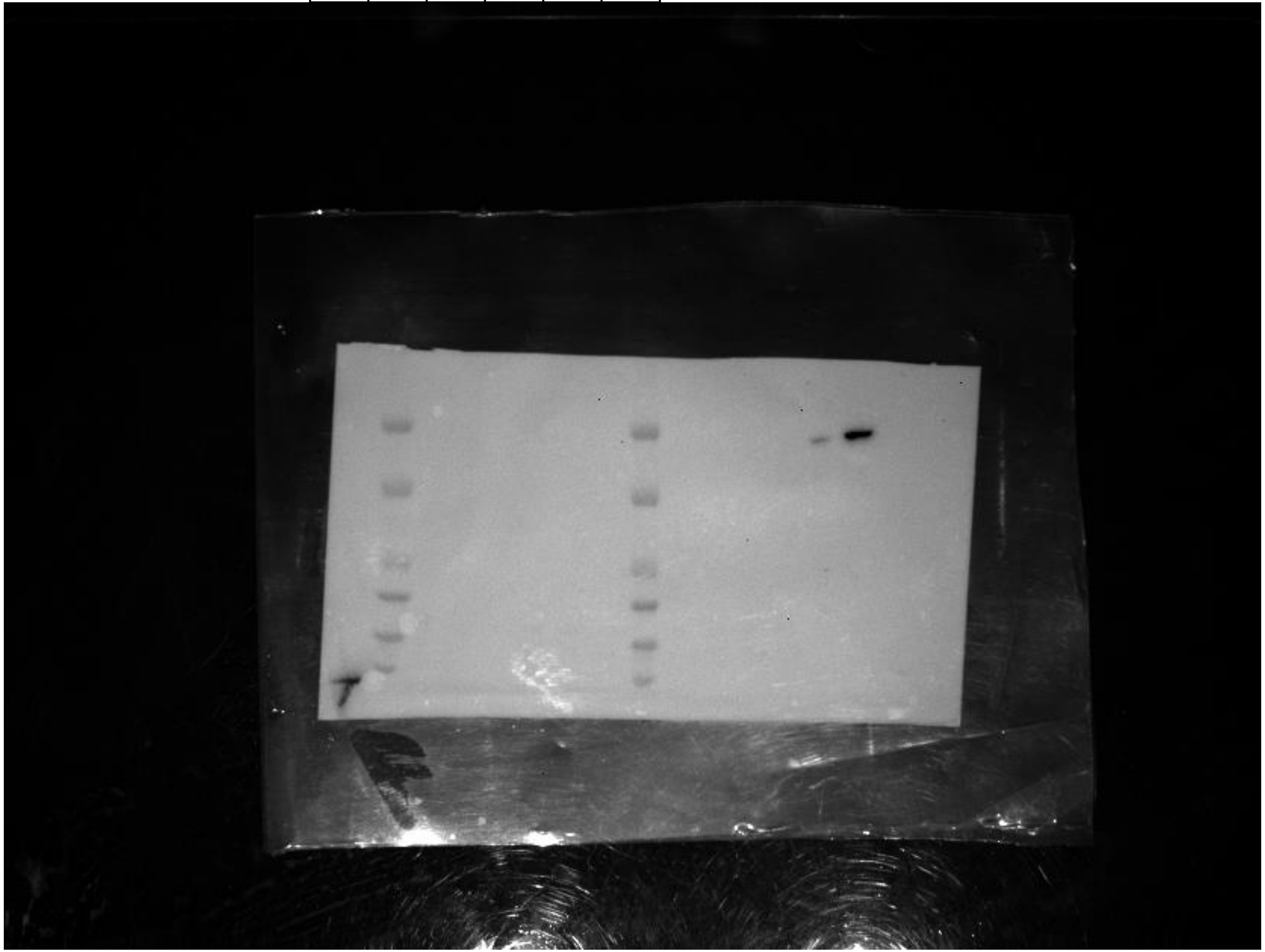

Figure 3C –MyLa- p21

Ctrl	25 nM	50 nM	100 nM	250 nM	500 nM

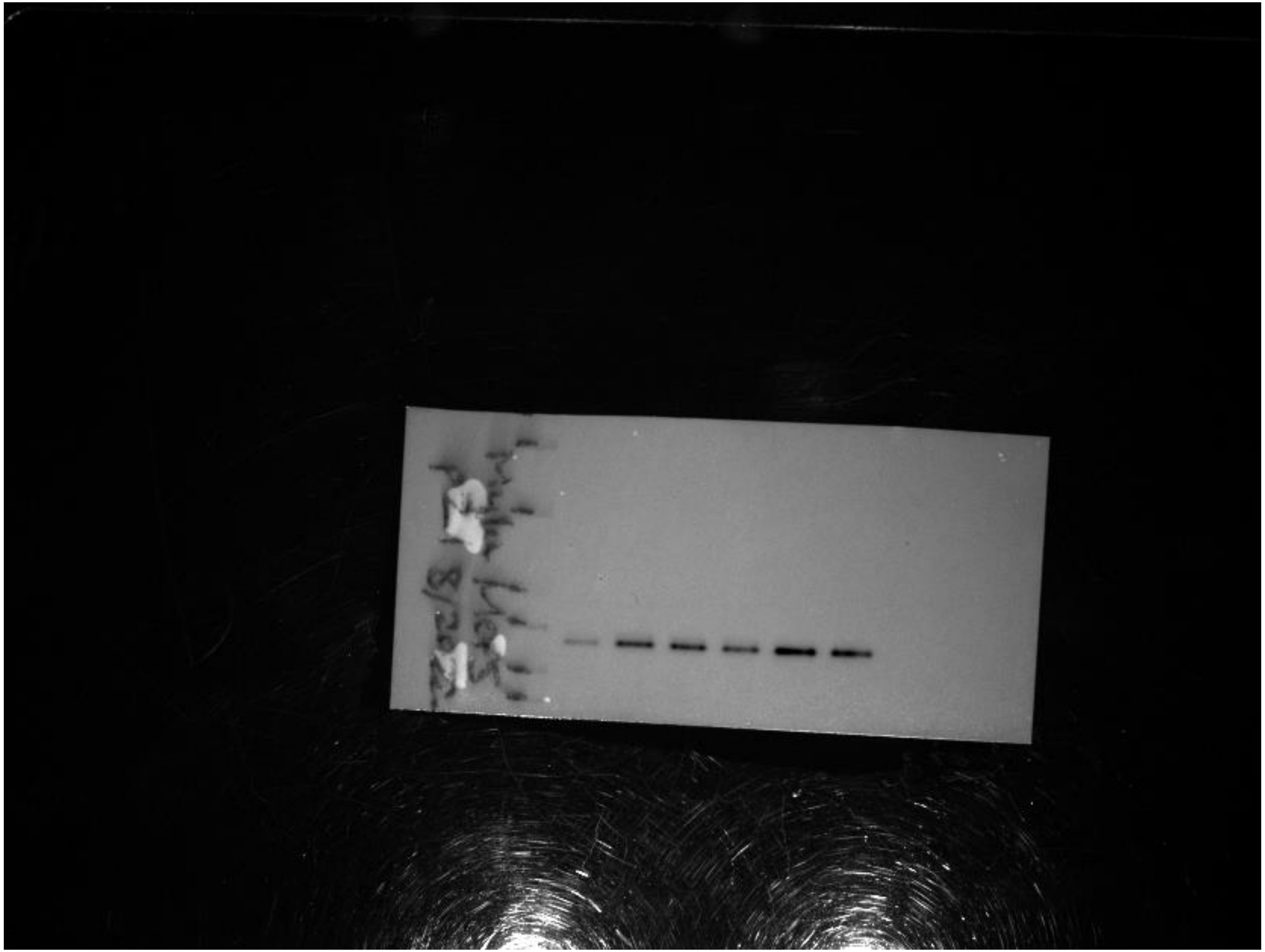

Figure 3C –MyLa- p27

Ctrl	25 nM	50 nM	100 nM	250 nM	500 nM

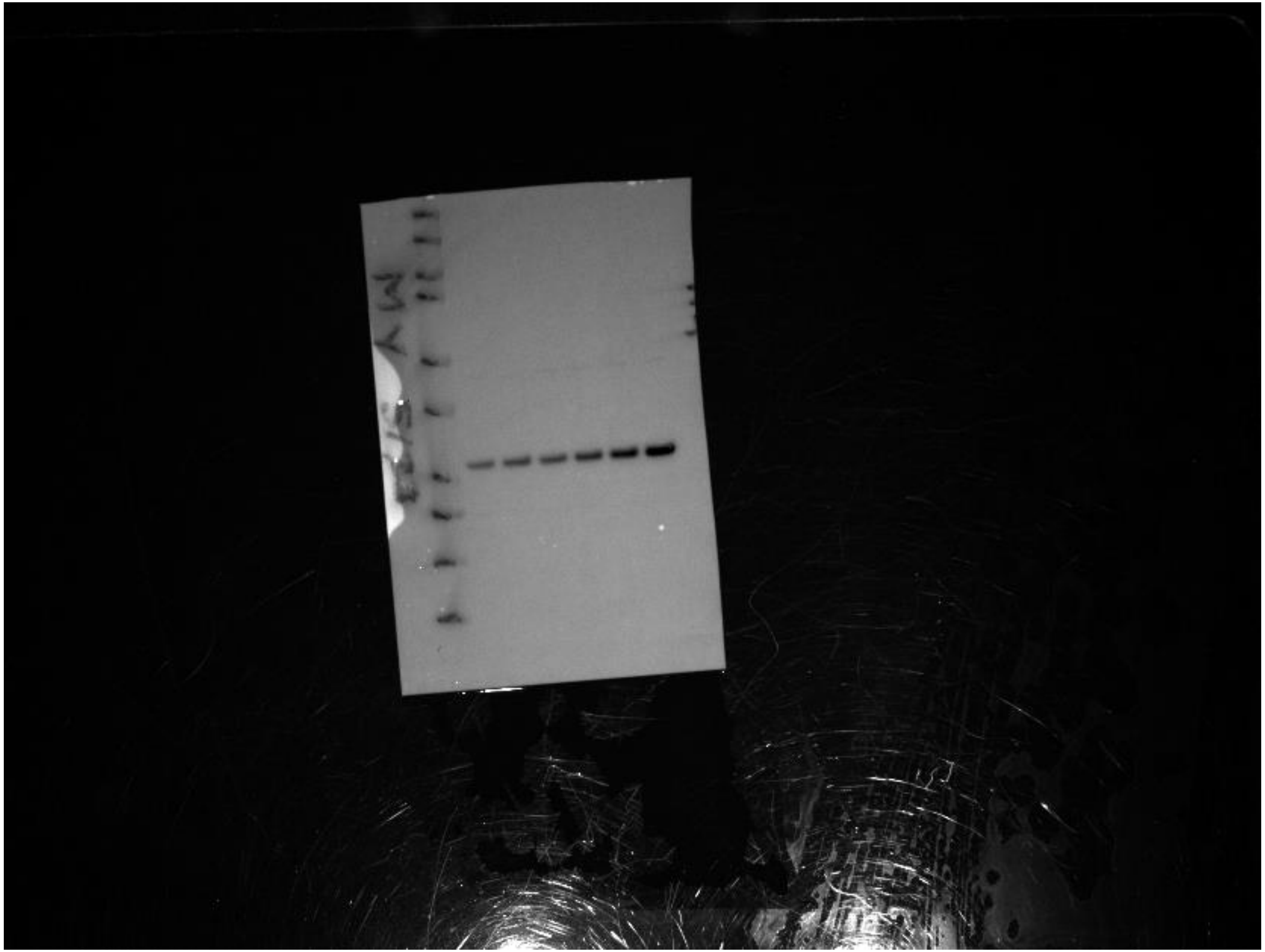

Figure 3C –MyLa- Survivin

Ctrl	25 nM	50 nM	100 nM	250 nM	500 nM

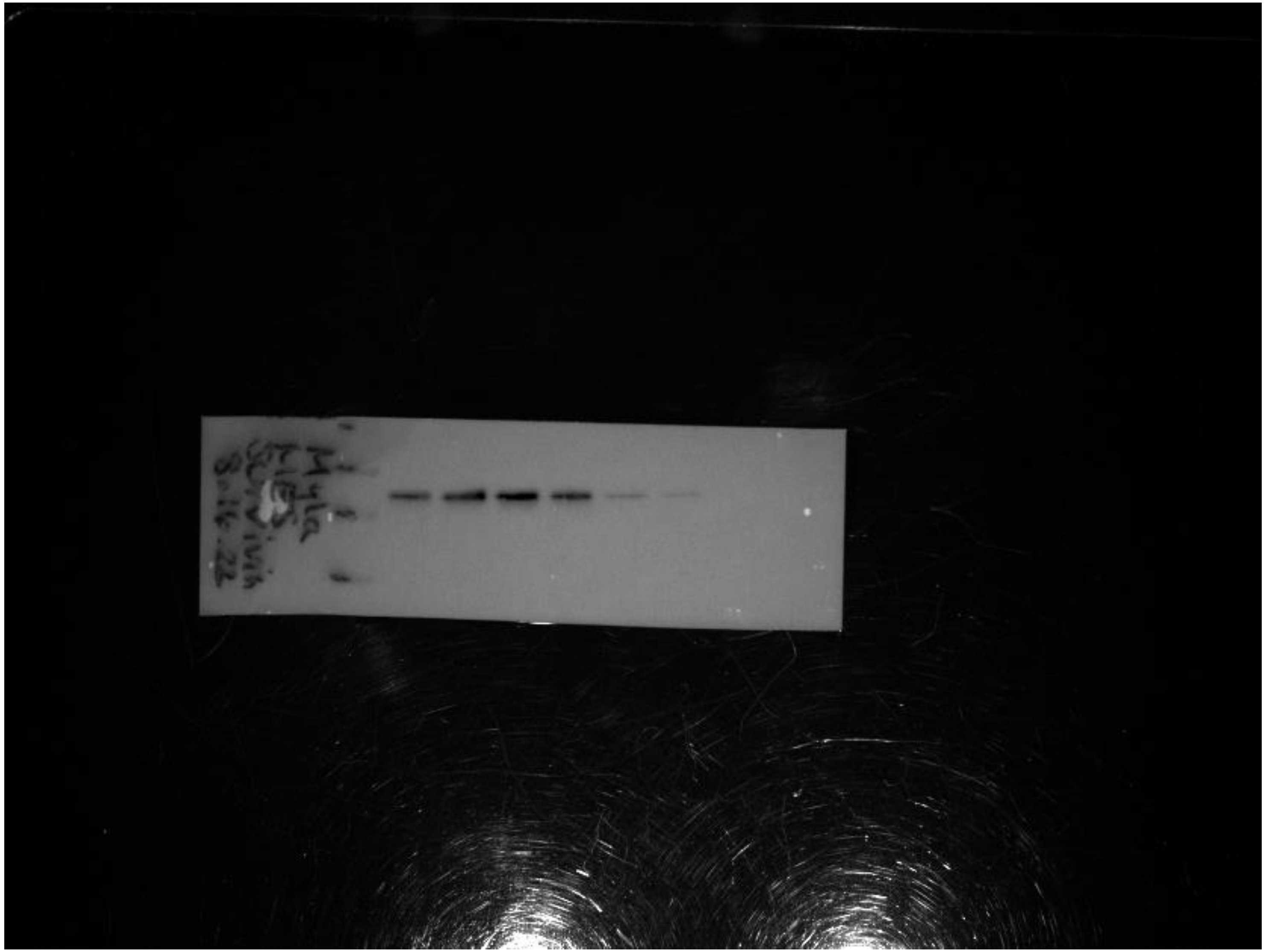

Figure 3C –MyLa- cCaspase3

Ctrl	25 nM	50 nM	100 nM	250 nM	500 nM

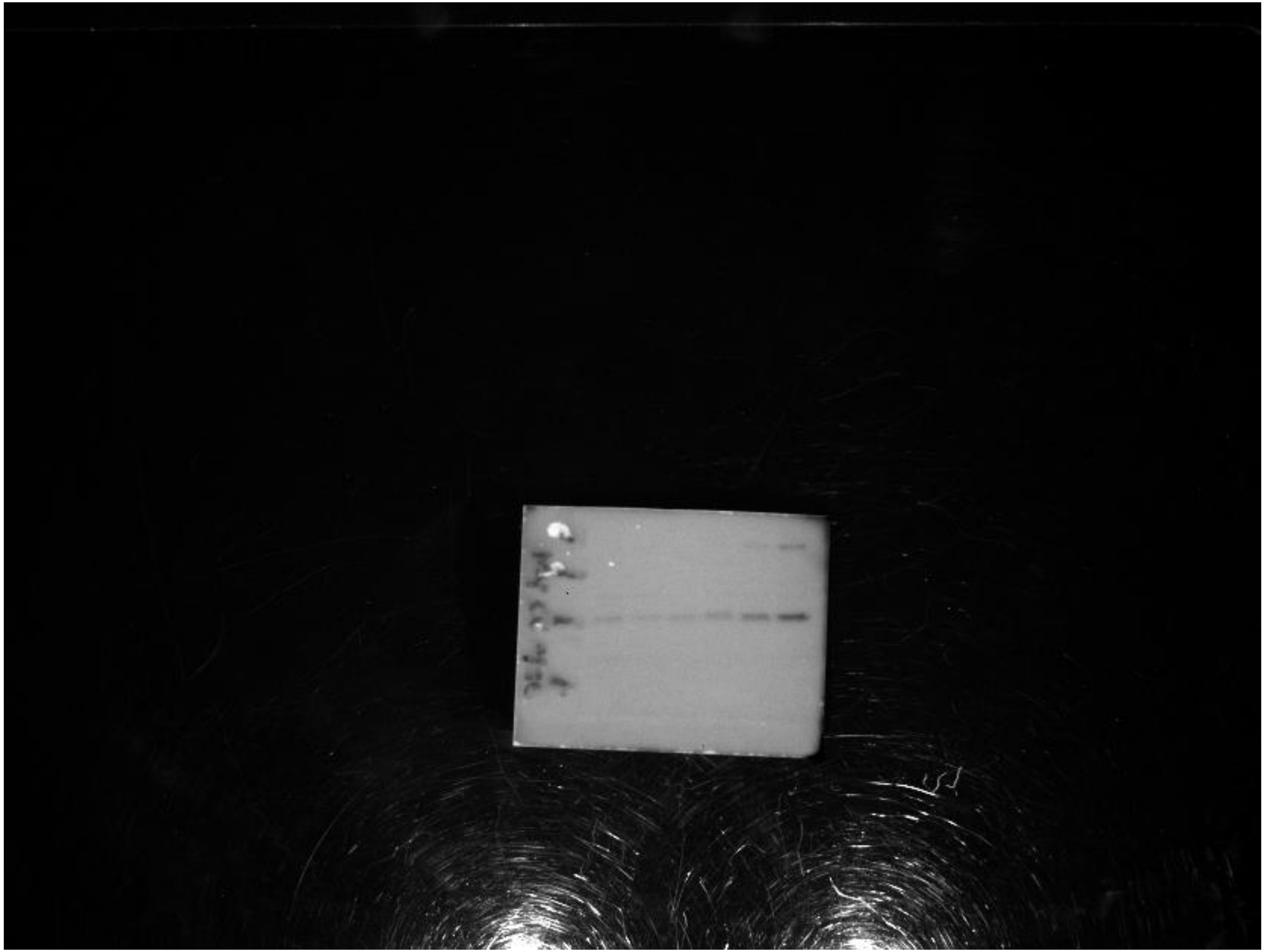

Figure 3C –MyLa- PARP

Ctrl	25 nM	50 nM	100 nM	250 nM	500 nM

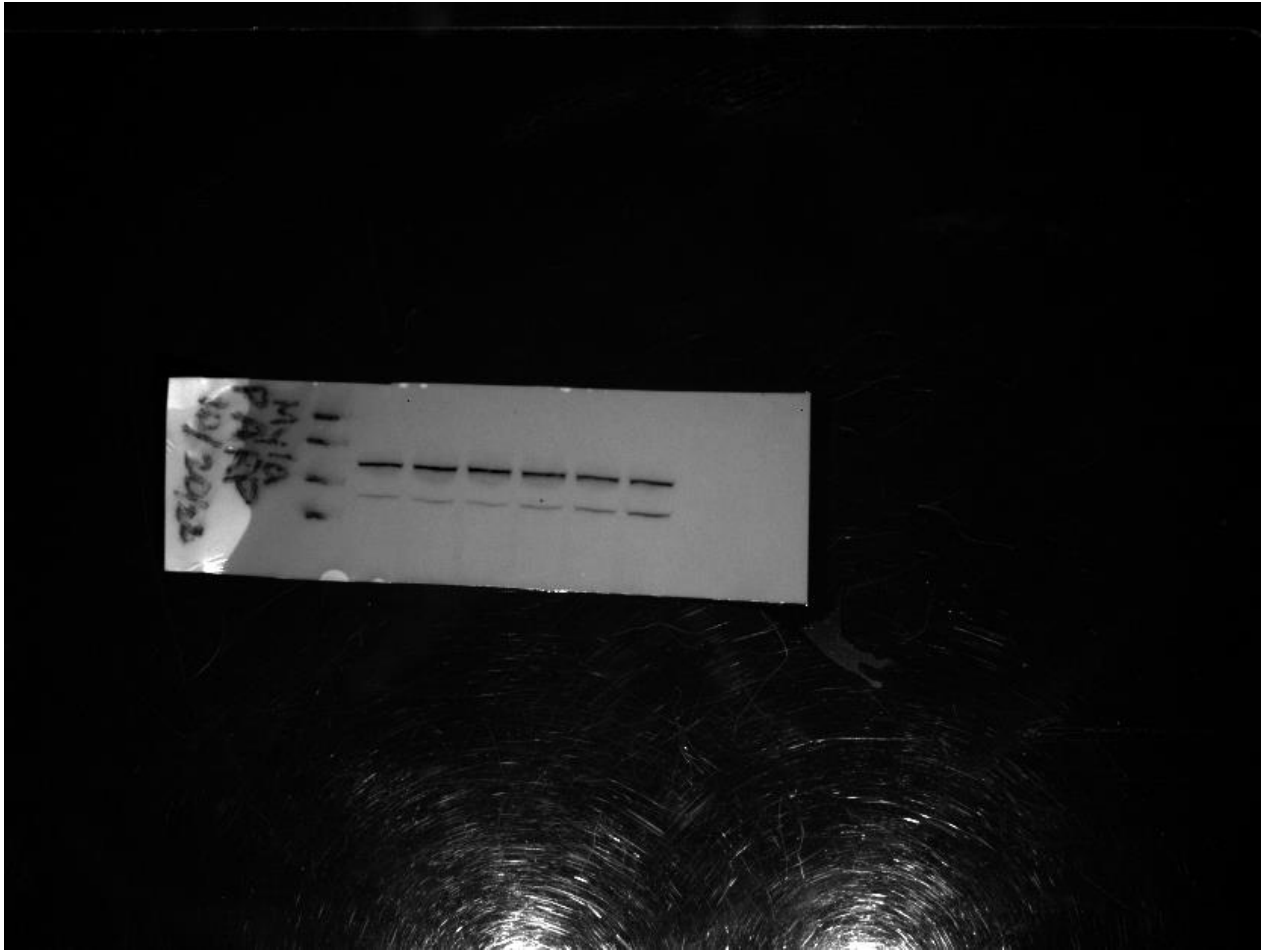

Figure 3C –MyLa- B-actin

Ctrl	25 nM	50 nM	100 nM	250 nM	500 nM

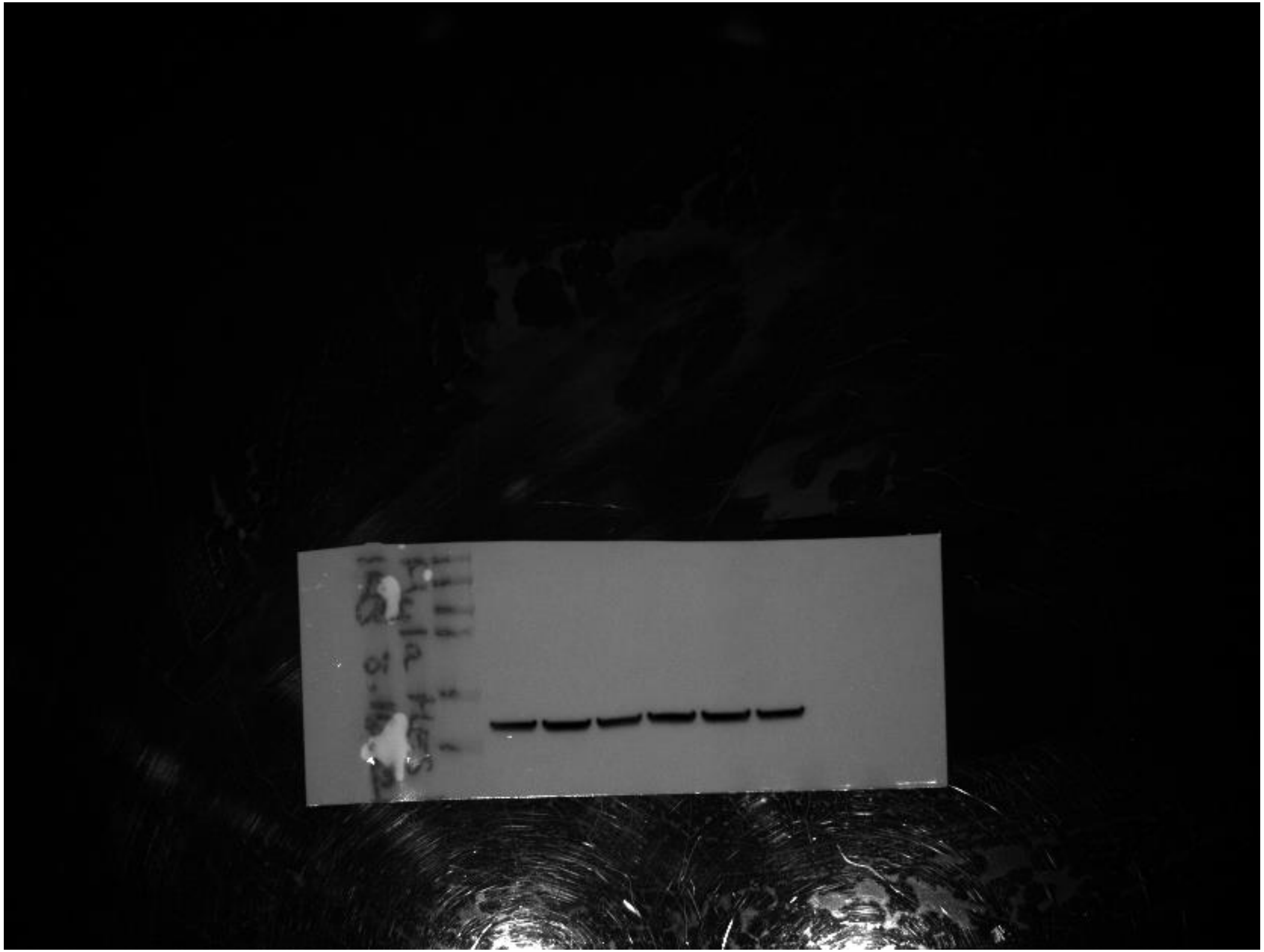

Figure 3C –H9-p21

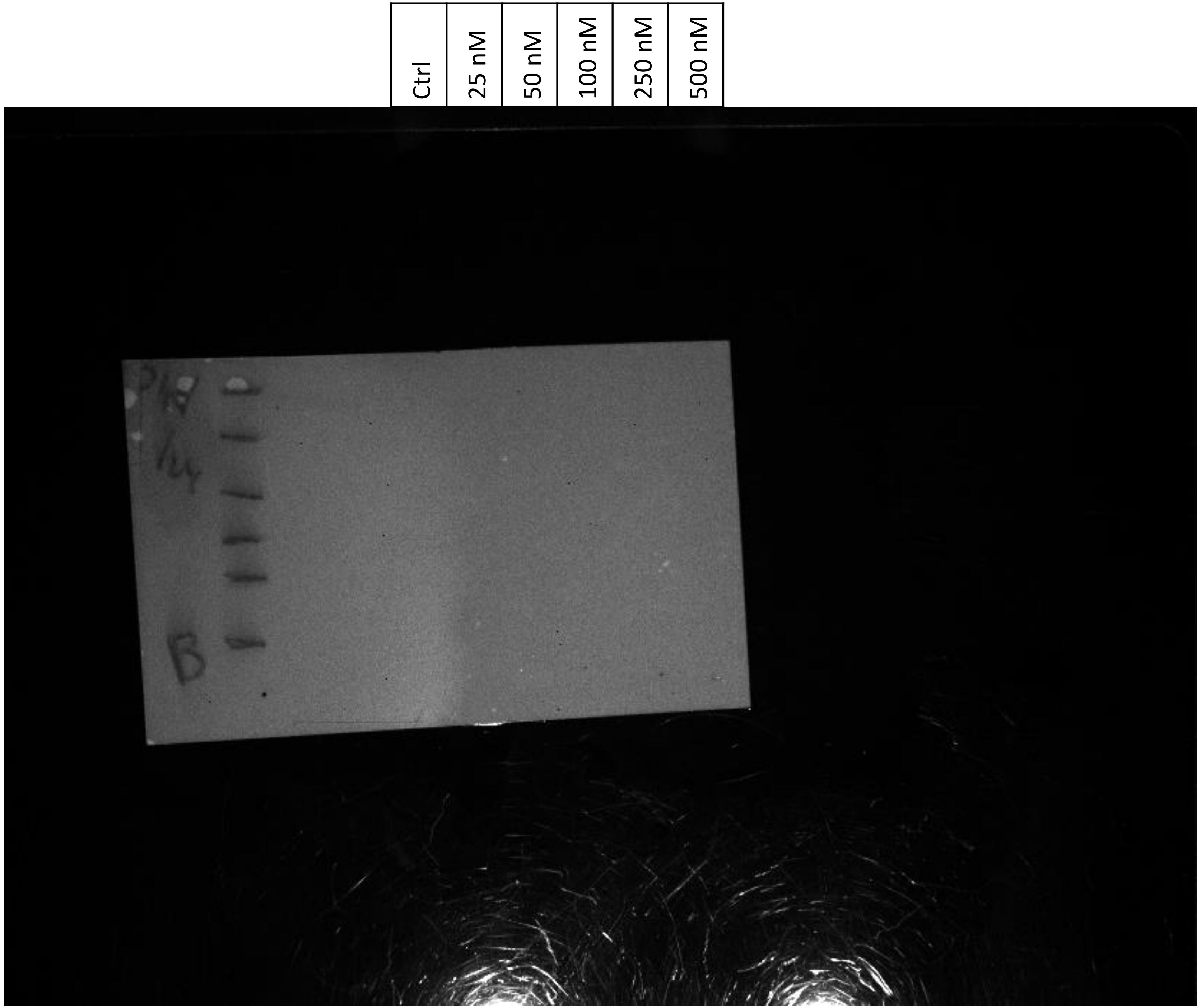

Figure 3C –H9-p27

Ctrl	25 nM	50 nM	100 nM	250 nM	500 nM

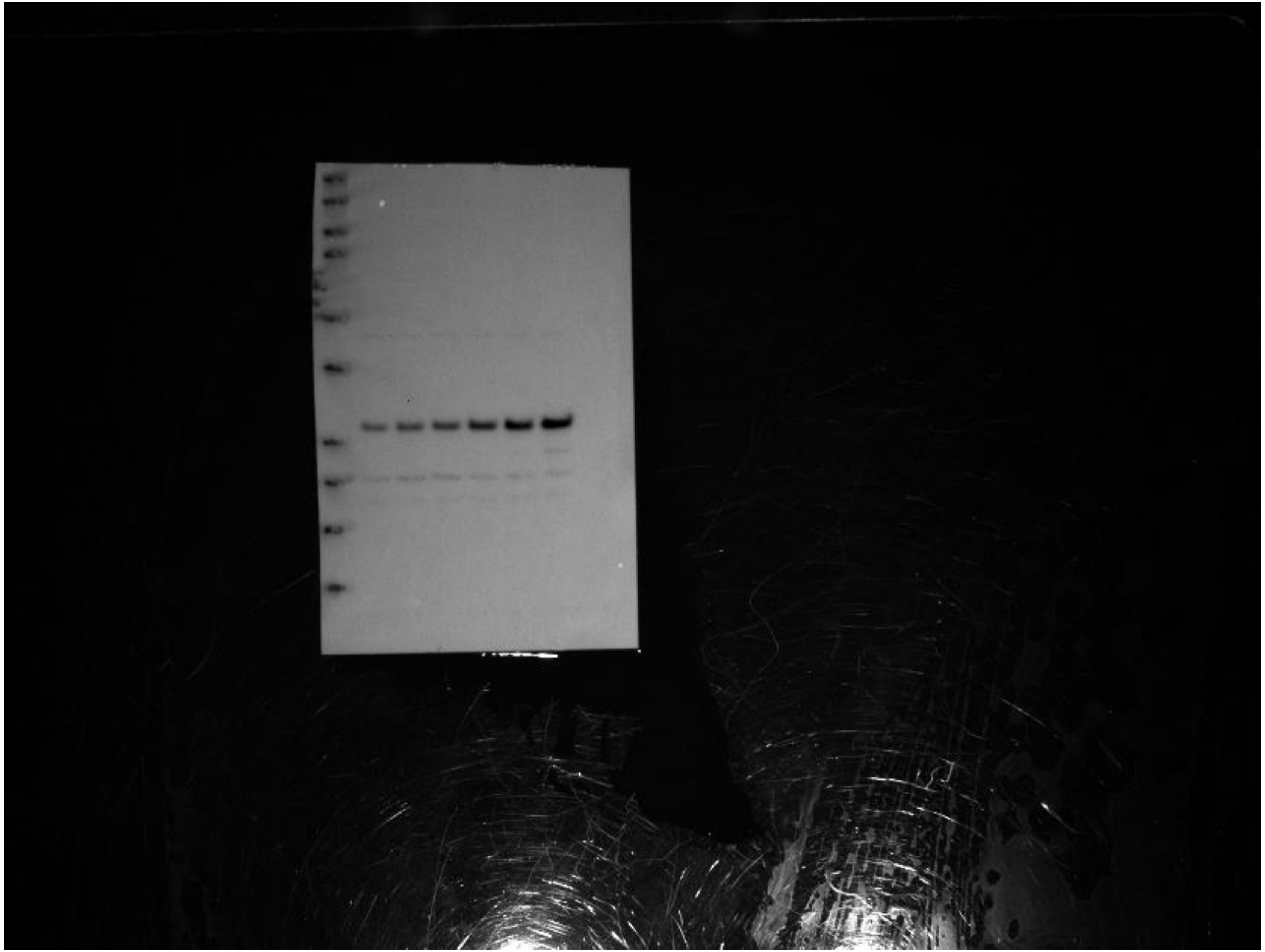

Figure 3C –H9-Survivin

Ctrl	25 nM	50 nM	100 nM	250 nM	500 nM

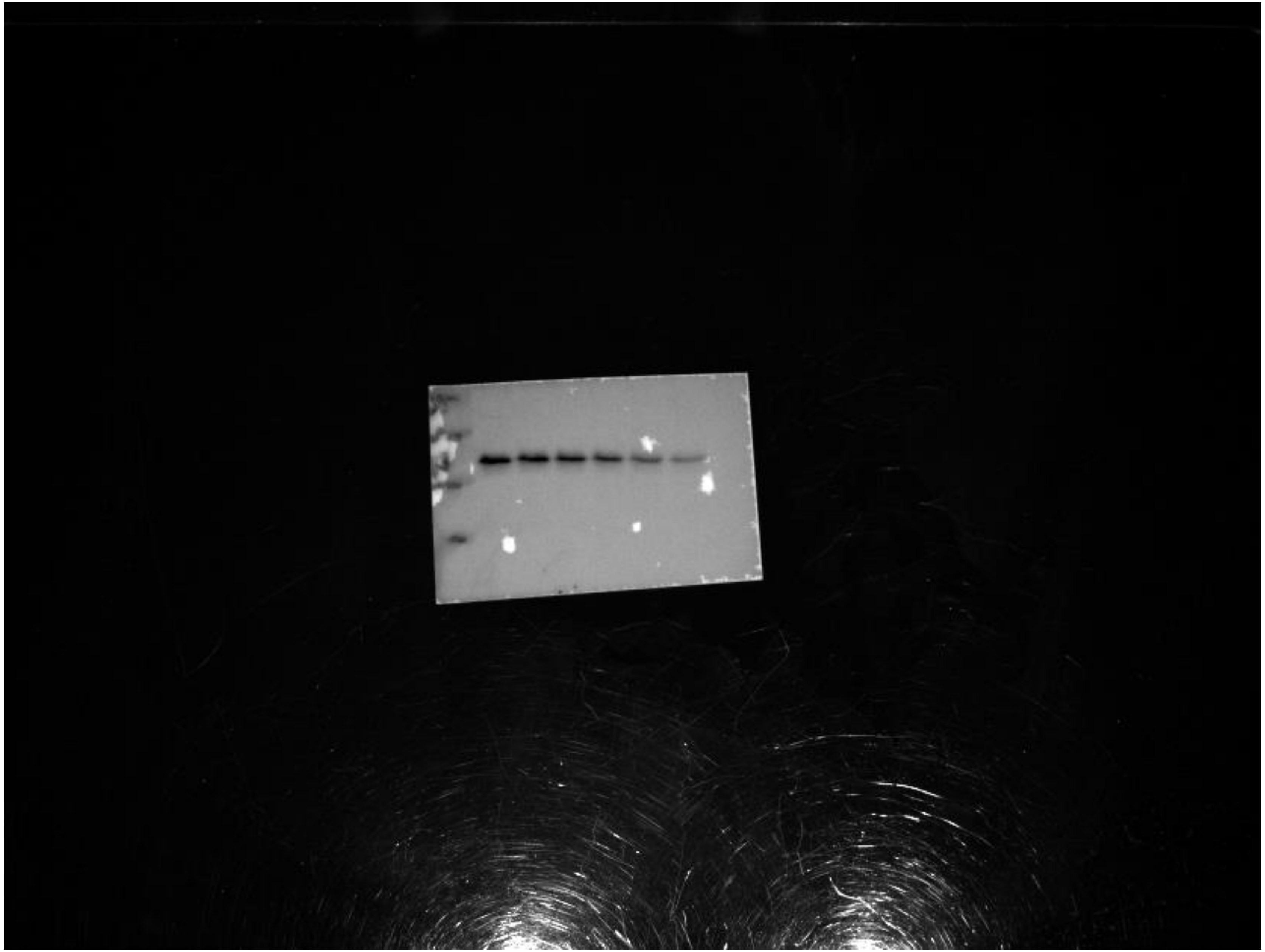

Figure 3C –H9-cCaspase

Ctrl	25 nM	50 nM	100 nM	250 nM	500 nM

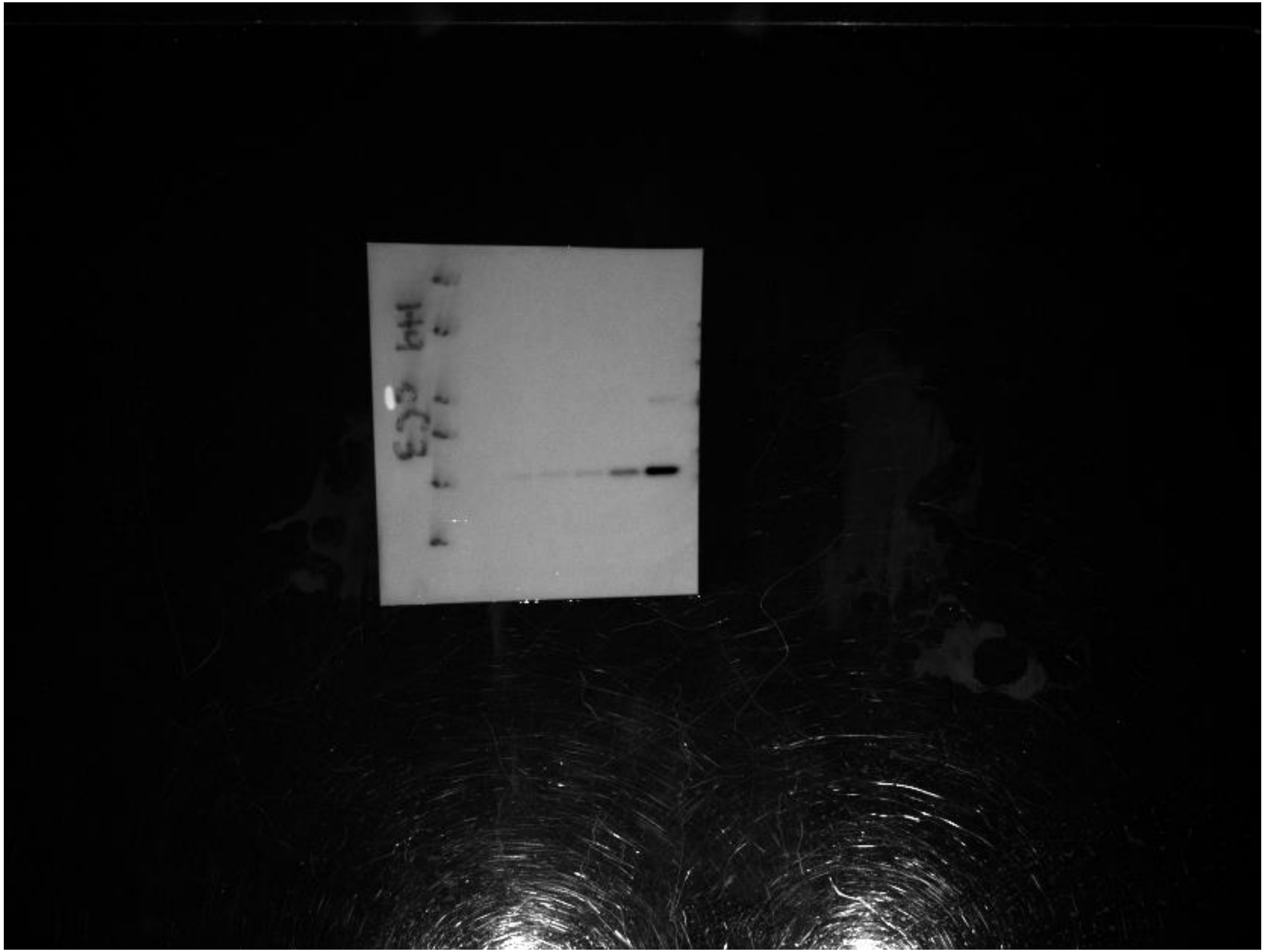

Figure 3C –H9-PARP

Ctrl	25 nM	50 nM	100 nM	250 nM	500 nM

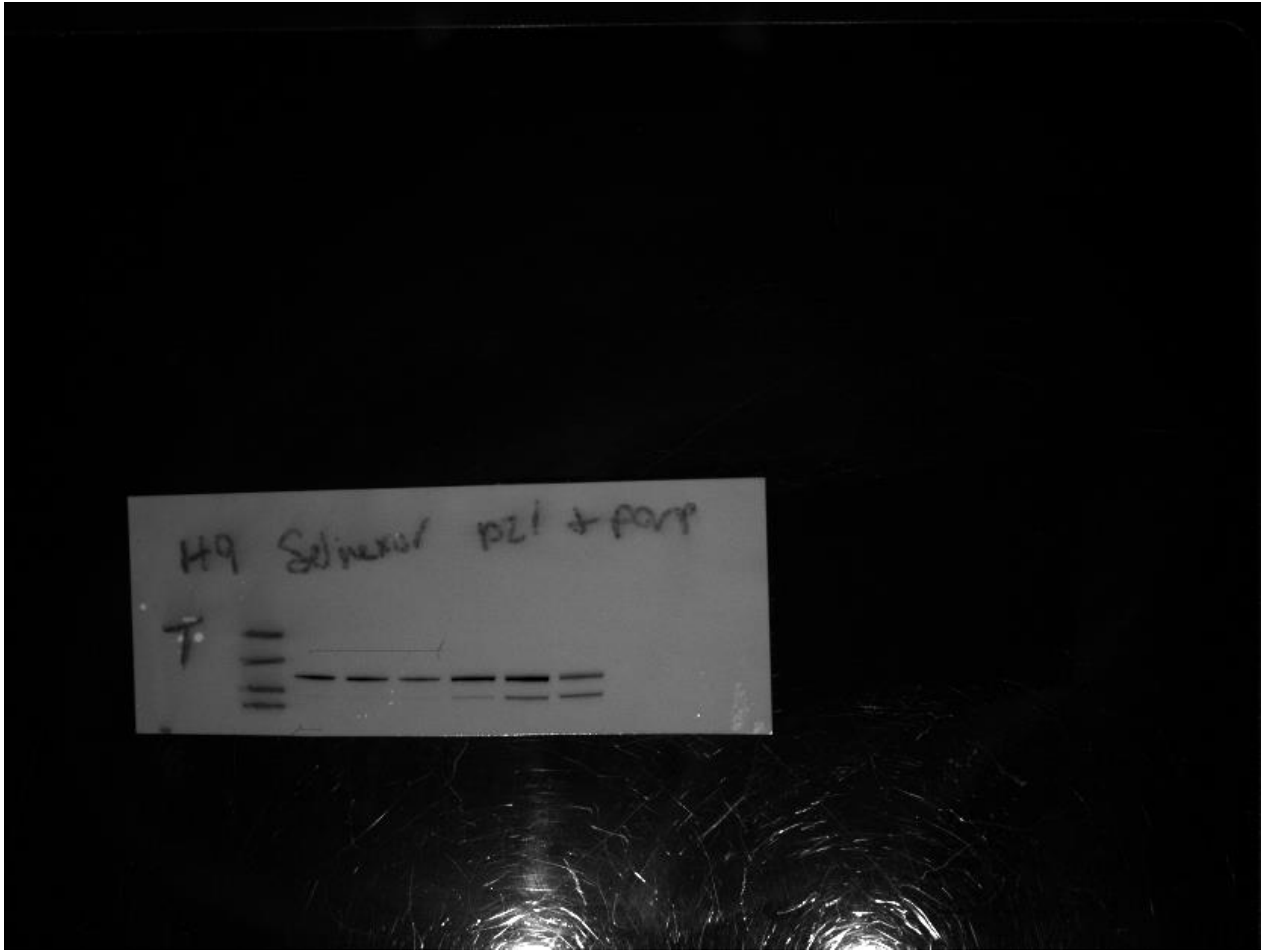

Figure 3C –H9-B-actin

Ctrl	25 nM	50 nM	100 nM	250 nM	500 nM

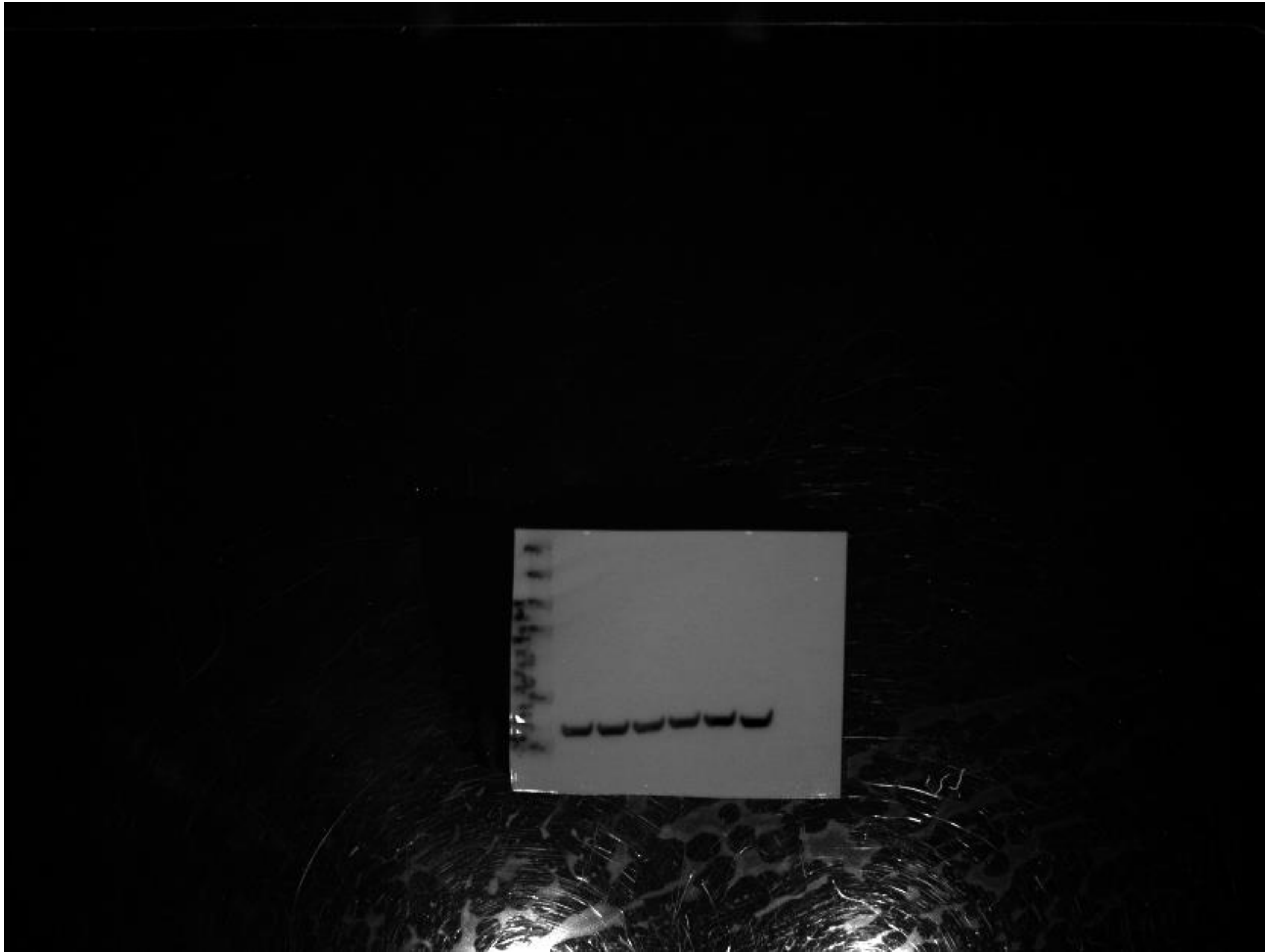

Figure 4B – MJ and H9 – XPO1

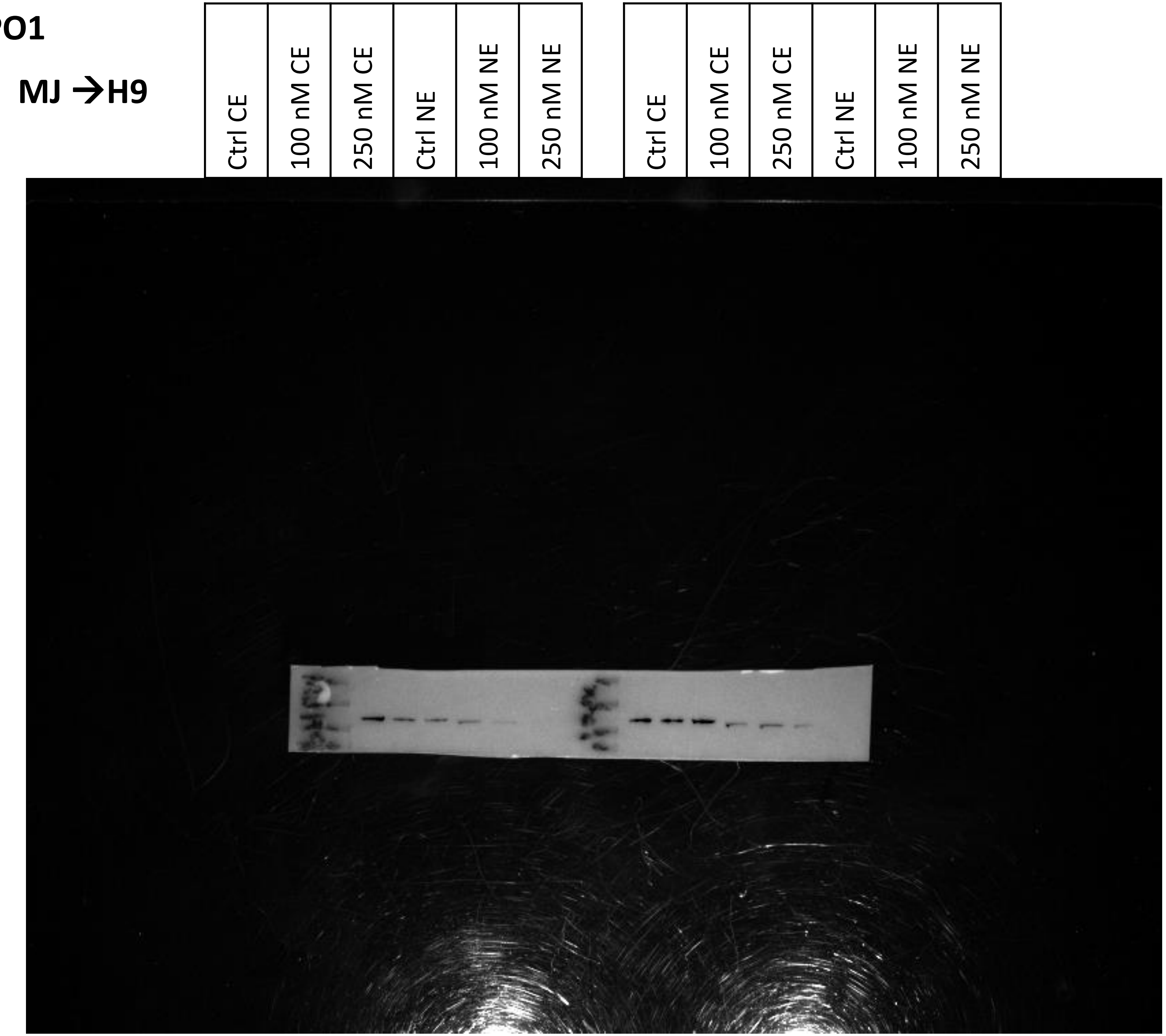

Figure 4B – MJ and H9 – p53

MJ →H9

Ctrl CE	100 nM CE	250 nM CE	Ctrl NE	100 nM NE	250 nM NE

Ctrl CE	100 nM CE	250 nM CE	Ctrl NE	100 nM NE	250 nM NE

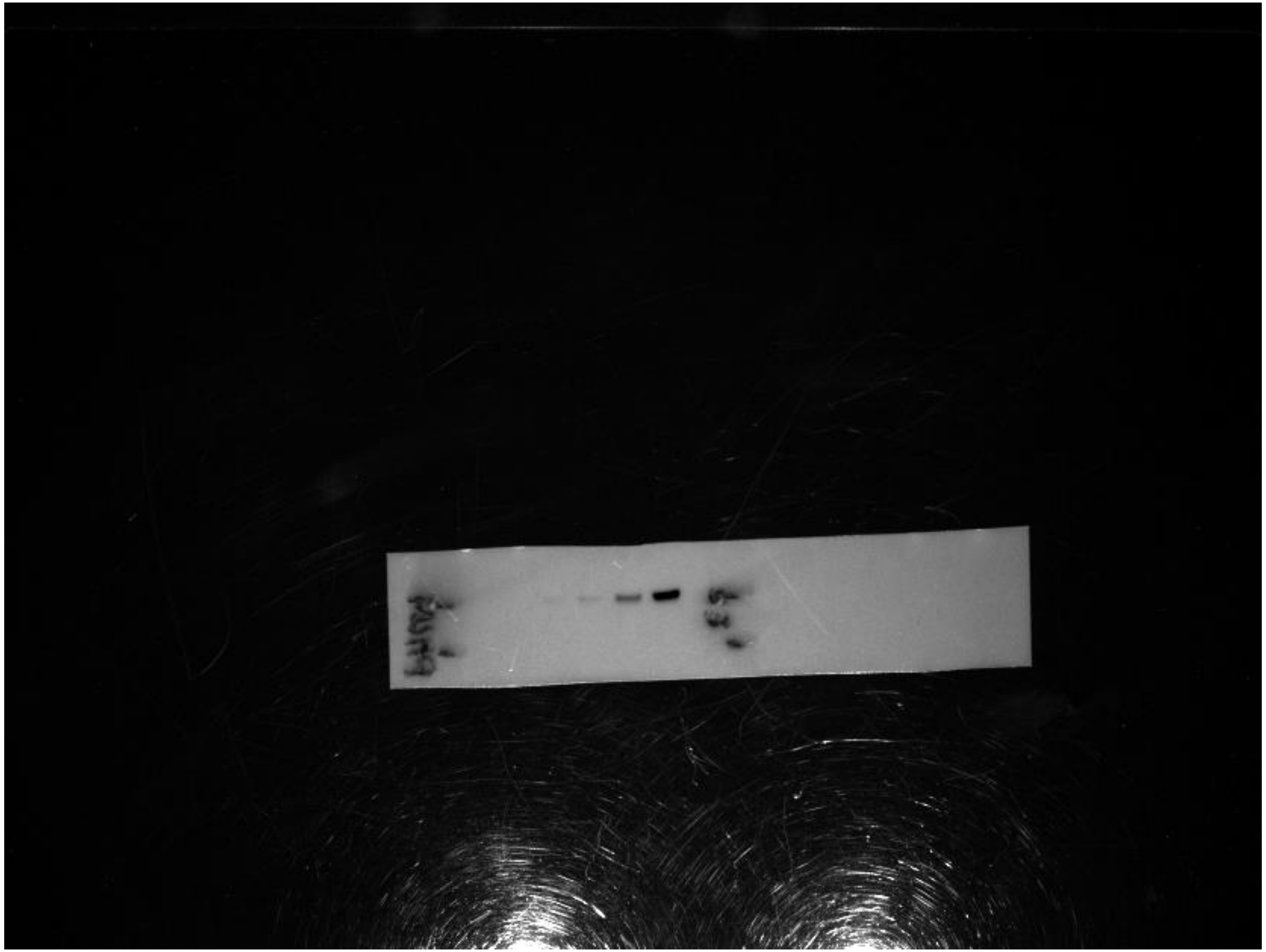

Figure 4B – MJ and H9 – p21

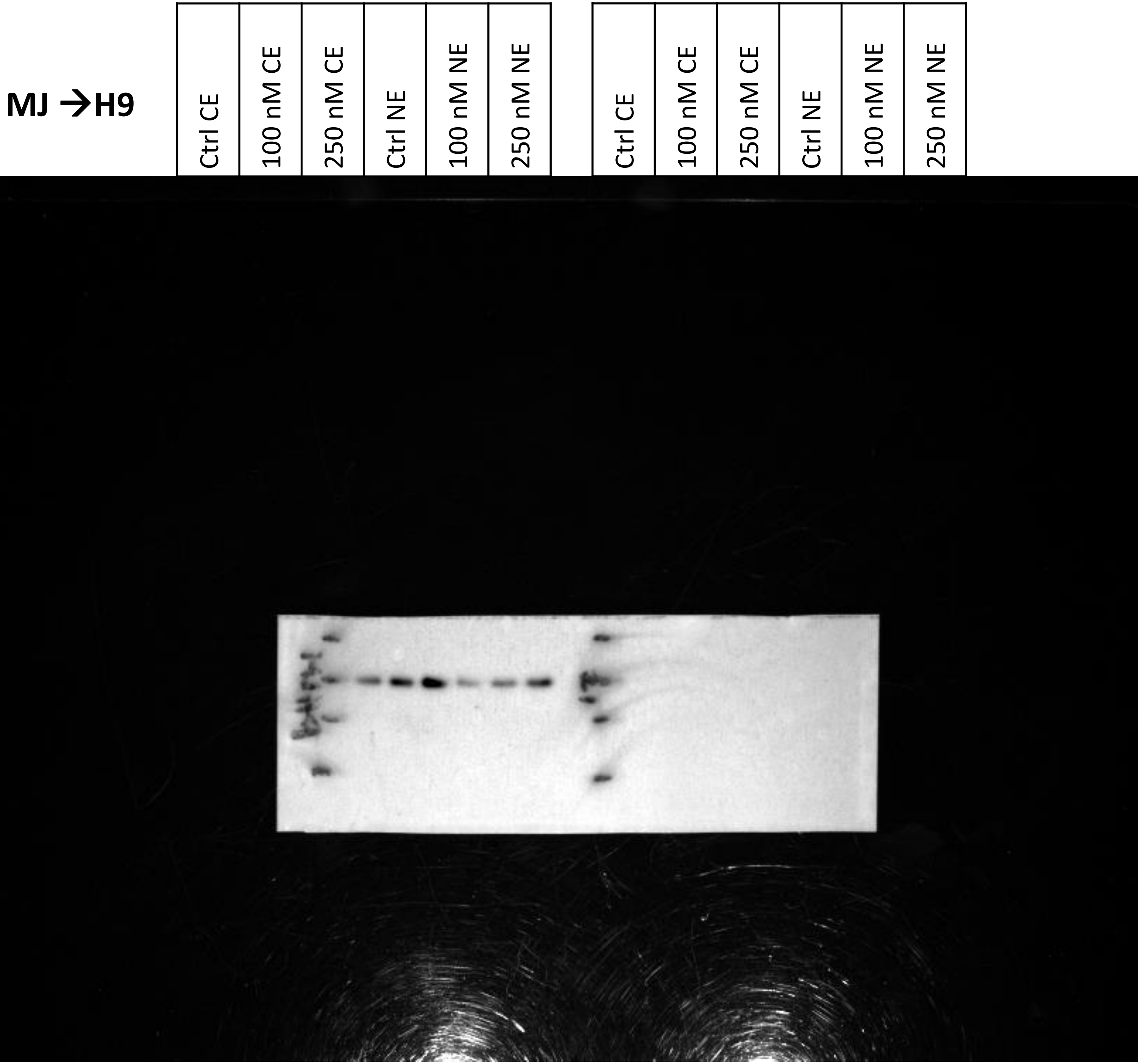

Figure 4B – MJ and H9 – p27

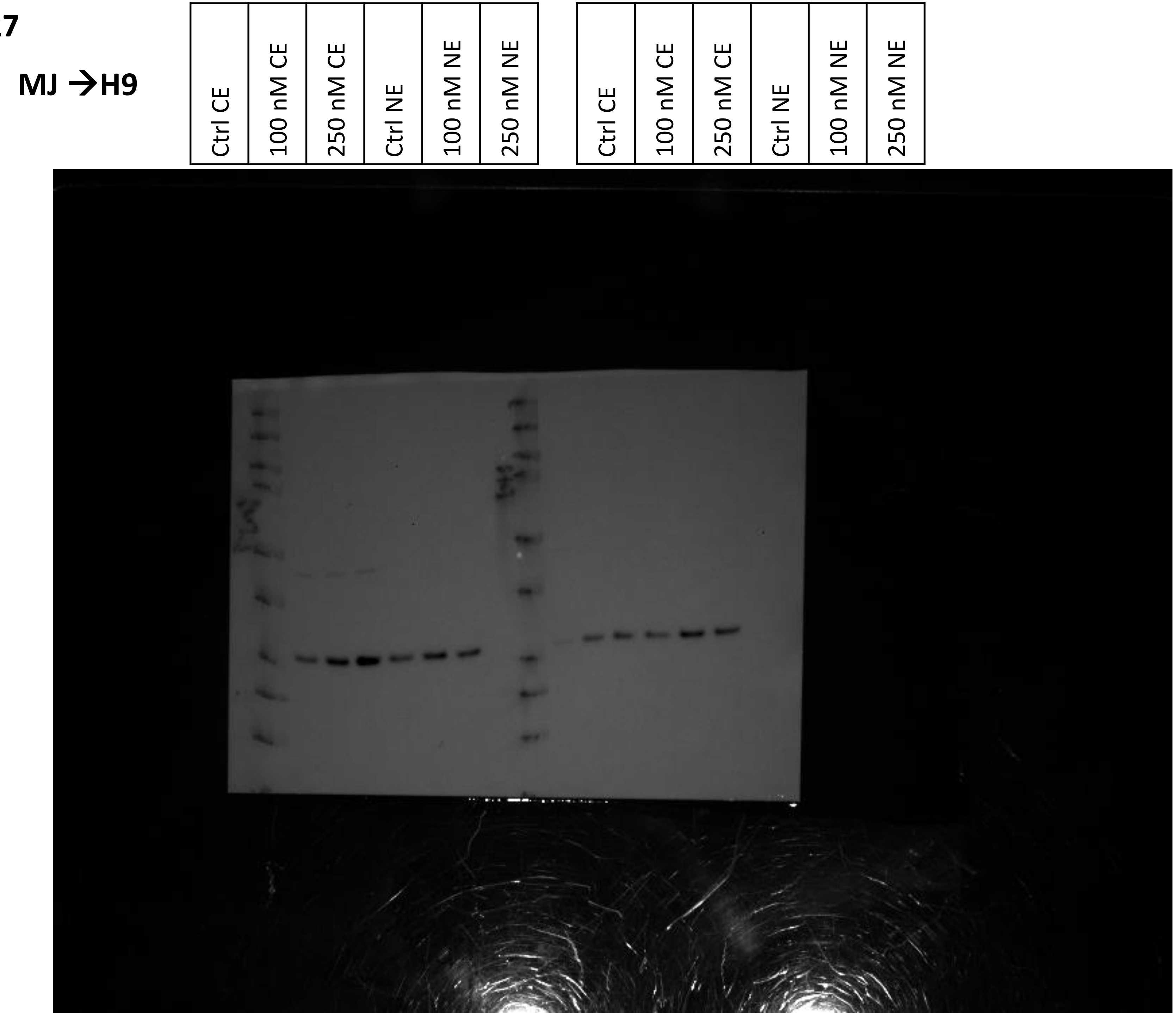

Figure 3 – MJ and H9 – GAPDH

MJ →H9

Ctrl CE	100 nM CE	250 nM CE	Ctrl NE	100 nM NE	250 nM NE	Ctrl CE	100 nM CE	250 nM CE	Ctrl NE	100 nM NE	250 nM NE

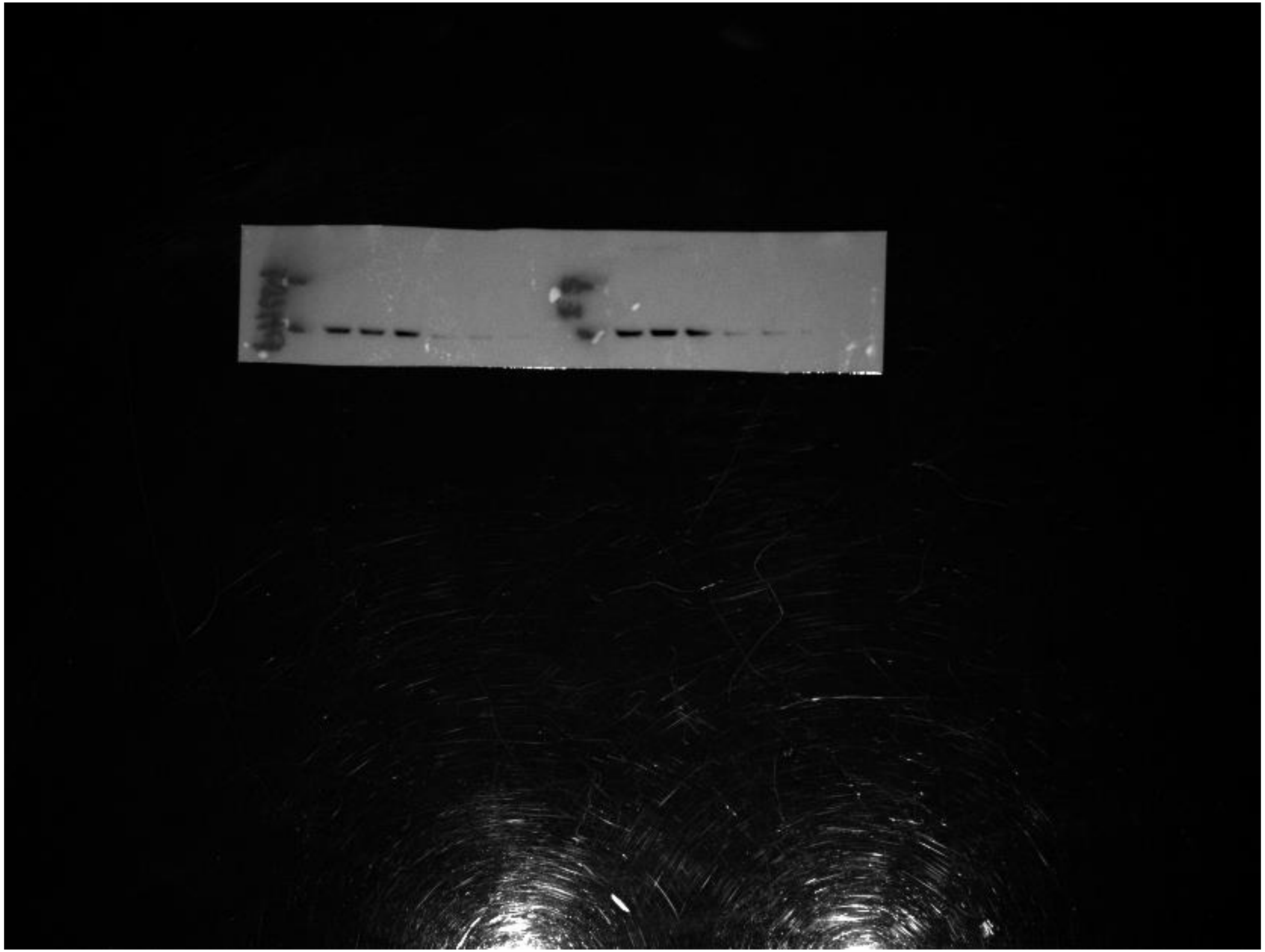

Figure 4B – MJ and H9 – Lamin A/C

MJ →H9

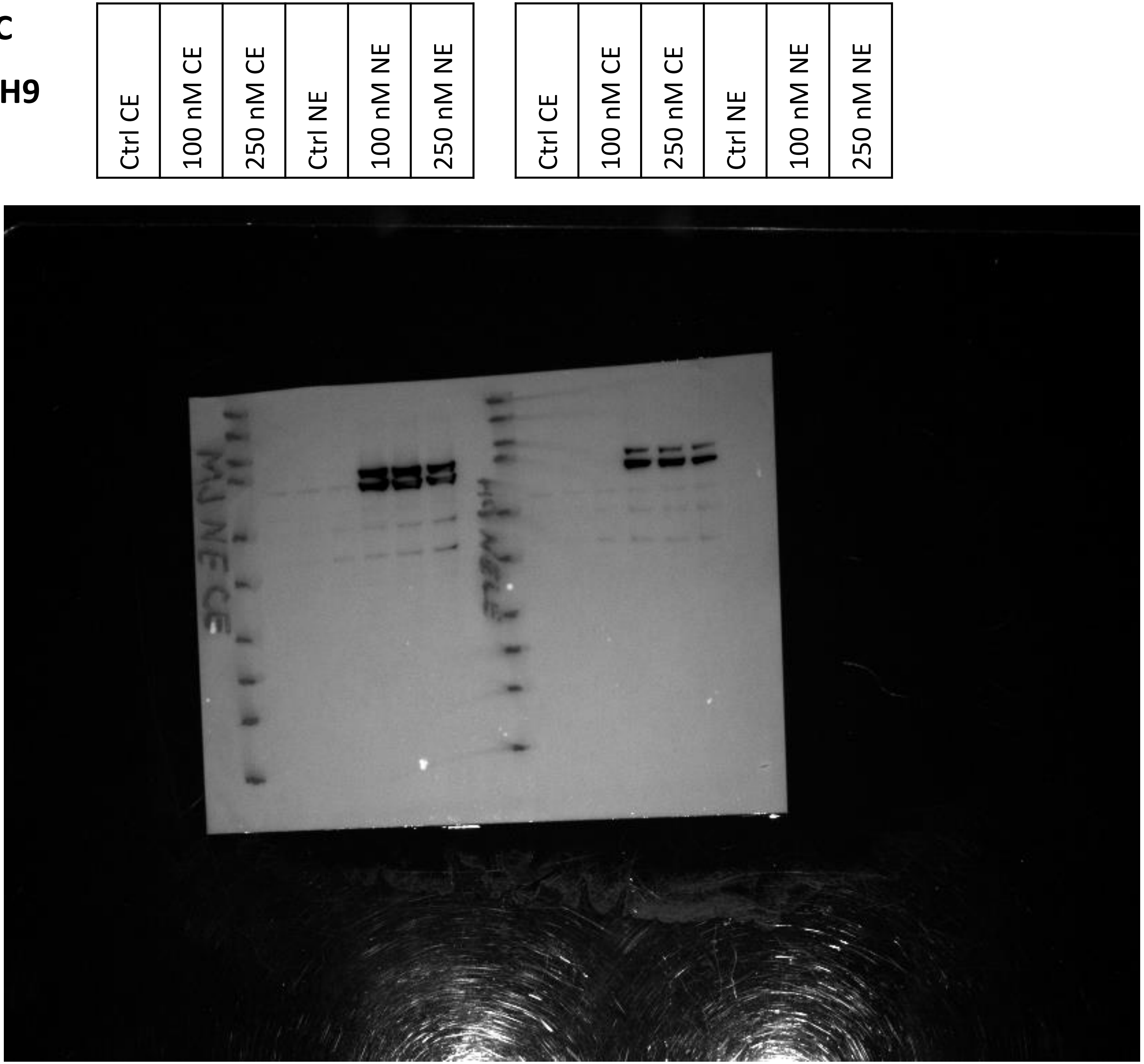

Figure 4B – MyLa– XPO1

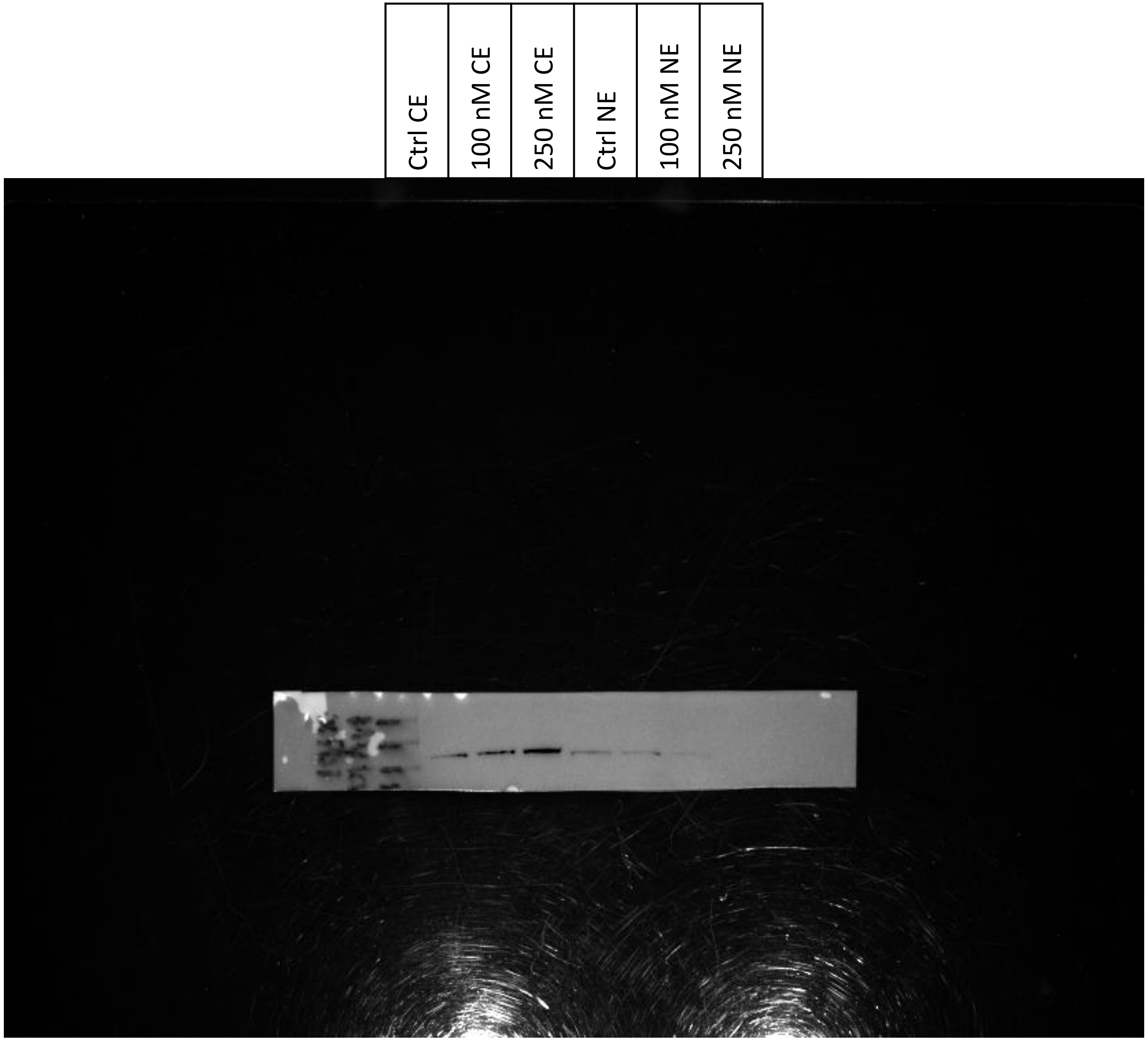

Figure 4B – MyLa– p53

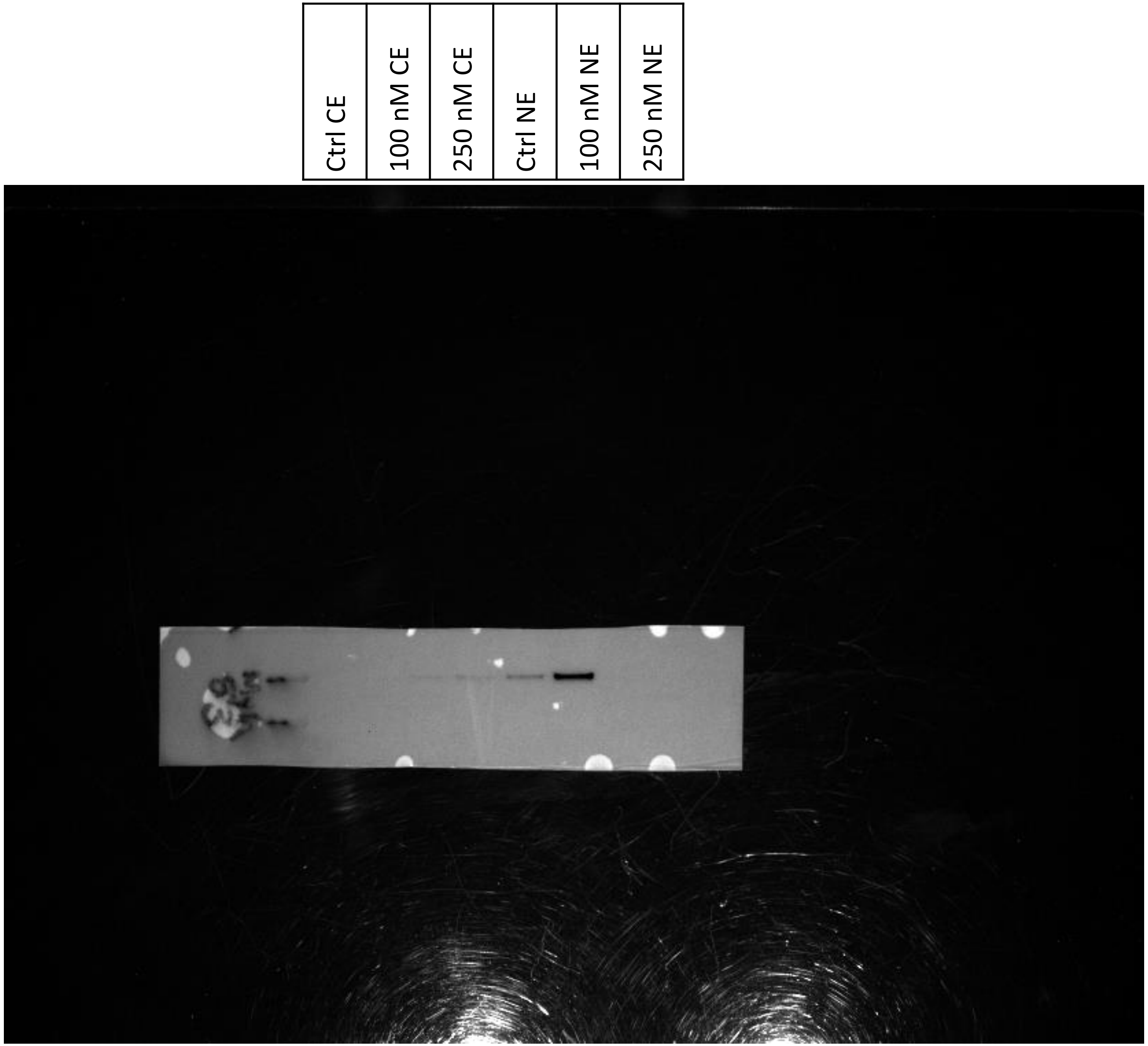

Figure 4B – MyLa– p21

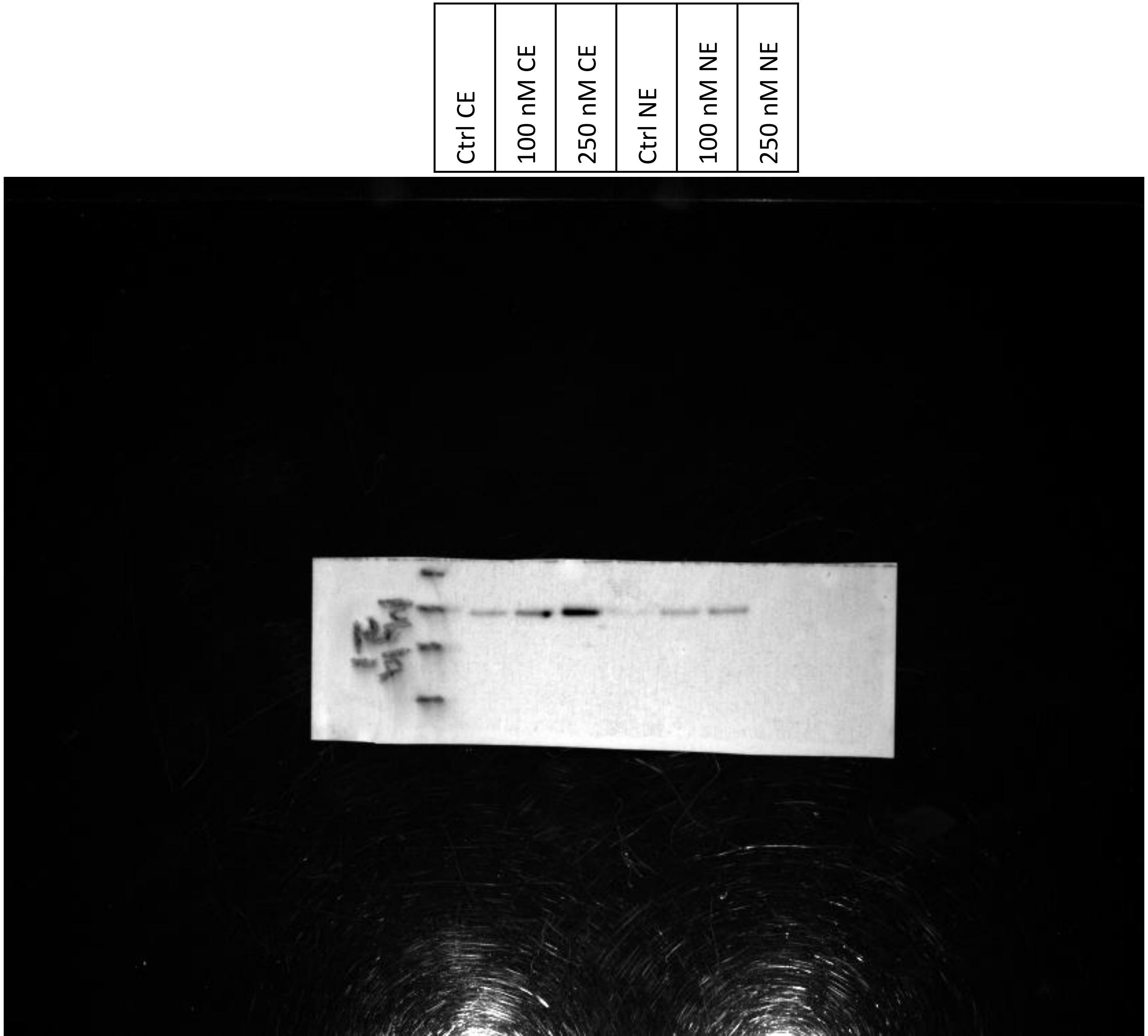

Figure 4B – MyLa– p27

Ctrl CE	100 nM CE	250 nM CE	Ctrl NE	100 nM NE	250 nM NE

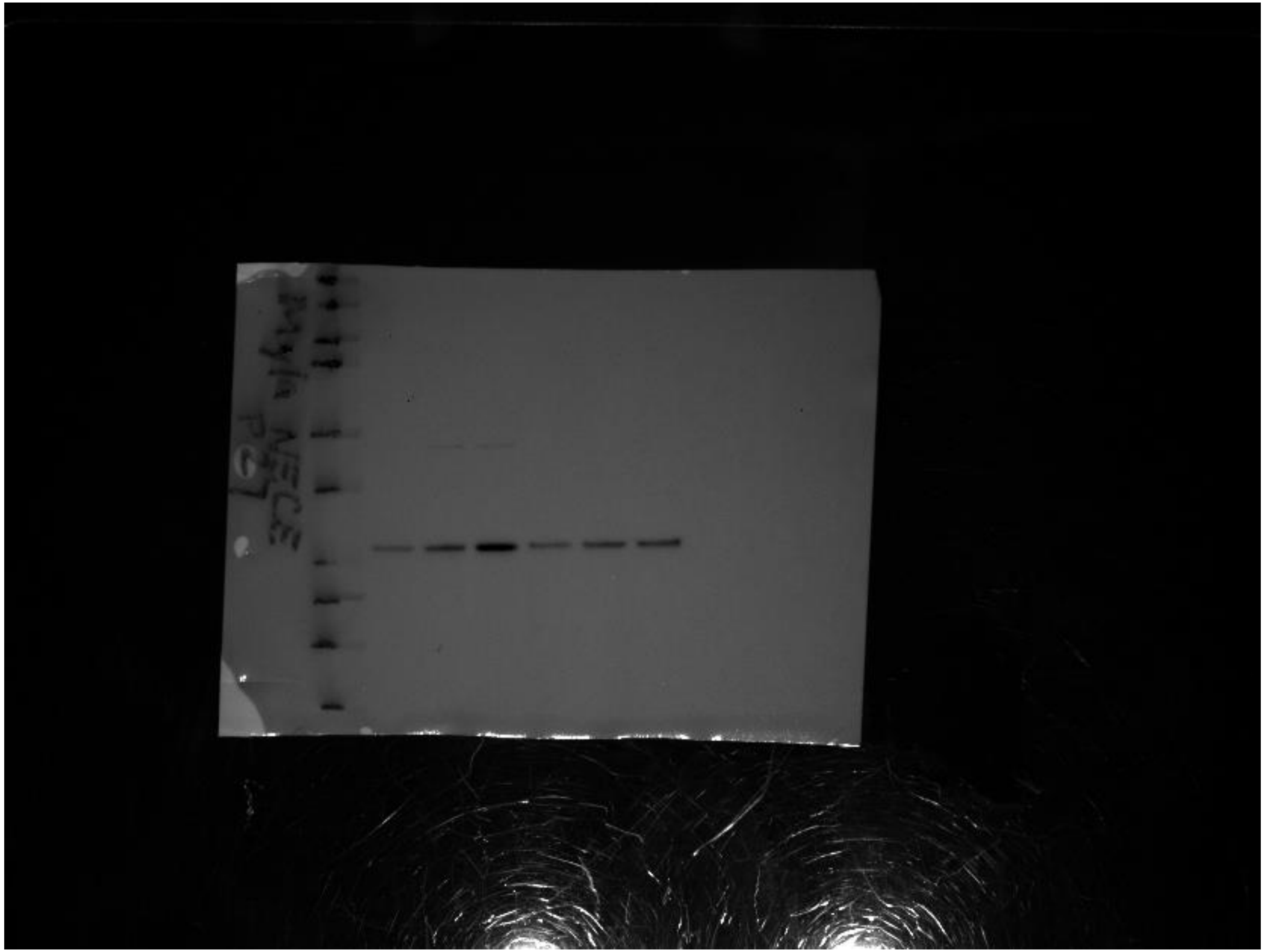

Figure 4B – MyLa– GAPDH

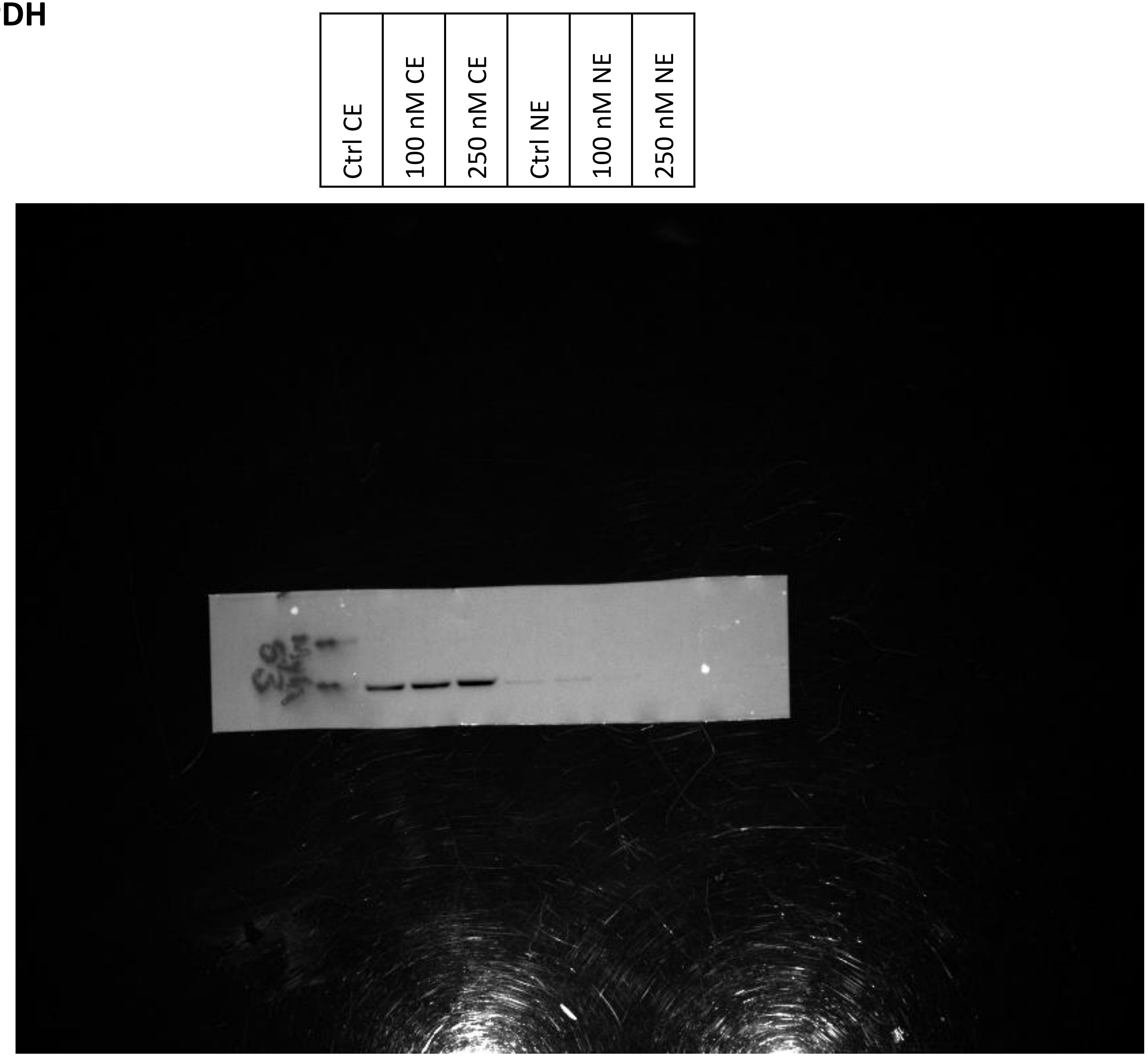

Figure 4B – MyLa– Lamin A/C

Ctrl CE	100 nM CE	250 nM CE	Ctrl NE	100 nM NE	250 nM NE

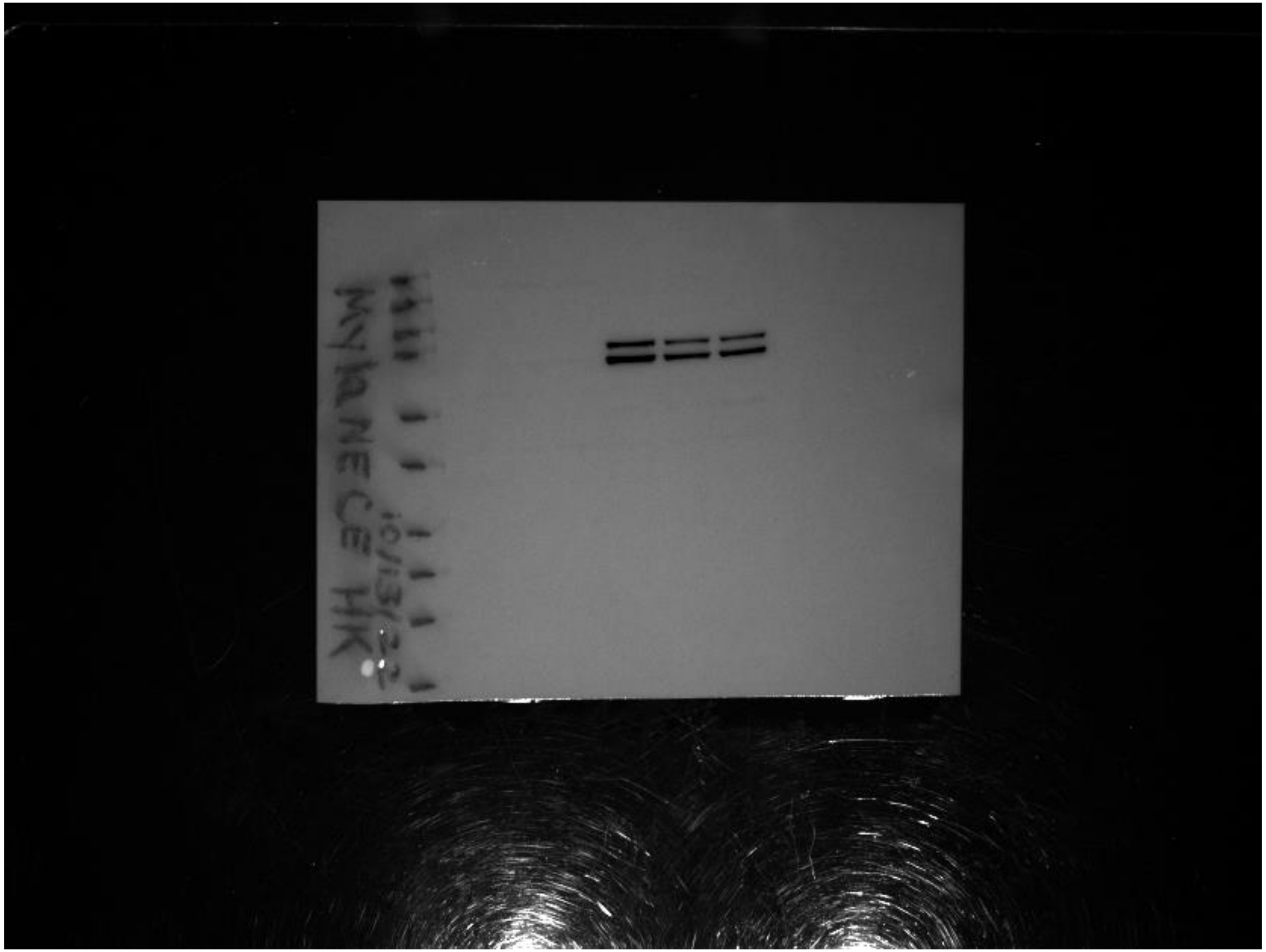

Supplement: Supplementary file 2 — Supplementary Information. [file 41598_2024_59994_MOESM2_ESM.pdf]
